# Bioinformatic mapping of a more precise *Aspergillus niger* degradome

**DOI:** 10.1038/s41598-020-80028-3

**Published:** 2021-01-12

**Authors:** Zixing Dong, Shuangshuang Yang, Byong H. Lee

**Affiliations:** 1 Henan Provincial Engineering Laboratory of Insect Bio-Reactor and Henan Key Laboratory of Ecological Security for Water Region of Mid-Line of South-To-North, Nanyang Normal University, 1638 Wolong Road, Nanyang, 473061 Henan People’s Republic of China; 2 College of Physical Education, Nanyang Normal University, Nanyang, 473061 People’s Republic of China; 3 Department of Microbiology/Immunology, McGill University, Montreal, QC Canada

**Keywords:** Proteases, Genomic analysis, Bioinformatics, Functional genomics, Genomics, Data mining, Protein function predictions, Fungal genomics, Functional genomics, Genome

## Abstract

*Aspergillus niger* has the ability to produce a large variety of proteases, which are of particular importance for protein digestion, intracellular protein turnover, cell signaling, flavour development, extracellular matrix remodeling and microbial defense. However, the *A. niger* degradome (the full repertoire of peptidases encoded by the *A. niger* genome) available is not accurate and comprehensive. Herein, we have utilized annotations of *A. niger* proteases in AspGD, JGI, and version 12.2 MEROPS database to compile an index of at least 232 putative proteases that are distributed into the 71 families/subfamilies and 26 clans of the 6 known catalytic classes, which represents ~ 1.64% of the 14,165 putative *A. niger* protein content. The composition of the *A. niger* degradome comprises ~ 7.3% aspartic, ~ 2.2% glutamic, ~ 6.0% threonine, ~ 17.7% cysteine, ~ 31.0% serine, and ~ 35.8% metallopeptidases. One hundred and two proteases have been reassigned into the above six classes, while the active sites and/or metal-binding residues of 110 proteases were recharacterized. The probable physiological functions and active site architectures of these peptidases were also investigated. This work provides a more precise overview of the complete degradome of *A. niger*, which will no doubt constitute a valuable resource and starting point for further experimental studies on the biochemical characterization and physiological roles of these proteases.

## Introduction

Proteases (also called peptidases, proteinases or proteolytic enzymes), catalyzing the cleavage of peptide bonds within proteins and polypeptides, are crucial for a variety of biological processes in organisms ranging from lower (viruses, bacteria, and fungi) to the higher organisms (mammals). Besides being essential for life, they also find wide applications in food, beverage, leather, pharmaceutical, textile and detergent industries^1,2^, and have been regarded as the most important industrial enzymes accounting for nearly 60% of the total enzyme market^3^. Proteases make up the most complex family of enzymes that possess different catalytic mechanisms with various active sites and divergent substrate specificities^4^. The MEROPS database (https://www.ebi.ac.uk/merops/) provides a structure-based catalogue and classification of peptidases, as well as their substrates and inhibitors^5^. Based on their catalytic sites, cleavage sites and substrate specificities, proteolytic enzymes are mainly divided into 7 classes: aspartic (A), cysteine (C), glutamic (G), metallo (M), serine (S) and threonine (T) peptidases, as well as asparagine peptide lyase (N)^5^. Protease classes are subdivided into clans according to their evolutionary relationship. Clans are further classified into families by common ancestry, while subfamilies have common structure yet unclear ancestry^6^. This classification system has facilitated the comprehensive identification and comparison of the degradomes (the complete repertoire of peptidases expressed in an organism at any particular moment or circumstance) in different organisms, especially mammals^7–13^. Recently, the Degradome database (http://degradome.uniovi.es/), containing the curated sets of known protease genes in human, chimpanzee, mouse and rat, has been developed^14^.

Aside from mammals, commercial proteases can also be produced by plants and microbes. Among them, microorganisms served as a preferred source of proteolytic enzymes for industrial applications owing to their high yield and productivity, broad catalytic and biochemical diversities, as well as susceptibility to genetic manipulation^15^. *Aspergillus niger,* a filamentous fungus with a long tradition of safe use in the production of various metabolites and industrial enzymes^16^, is able to grow on a wide range of substrates under various environmental conditions due to its ability to secrete large amounts of hydrolytic enzymes, including proteases^17,18^. So far, however, a comprehensive and precise view of the *A. niger* degradome is not available, and only approximately 8.2% *A. niger* proteases have been molecularly and biochemically characterized based on literature survey^19^.

Currently, the genome sequencing of 14 *A. niger* strains have been completed, among which the genome sequences of *A. niger* strains CBS 513.88^18^ and ATCC 1015^20^ have been extensively studied. This has opened the opportunity to investigate the complexity of the *A. niger* degradome. Although approximately 307 putative peptidases and 135 non-peptidase homologues have been deposited in version 12.2 MEROPS database (https://www.ebi.ac.uk/merops/cgi-bin/speccards?sp=sp000086;type=peptidase), many putative proteases are either not peptidases or identical to others. Additionally, as the *A. niger* genome is still subject to revisions, the classification, active sites and metal binding residues of these peptidases also need to be revised. In the present study, we utilized annotations of *A. niger* proteases in AspGD, JGI, and version 12.2 MEROPS database to identify putative proteases. MEROPS classification system (version 12.2)^5^ was then employed to reassign these proteases, followed by in silico characterization of their active sites, active site architectures, and metal binding residues using various bioinformatics tools. The degradome presented here provides a global view of the arsenal of proteases harbored by *A. niger*, thereby facilitating in depth studies on their physiological roles, and the molecular and biochemical characterization of this important class of enzymes.

## Results

### Whole-genome analysis of the *A. niger* Degradome

Using the primary information retrieved from the genomes of two *A. niger* strains CBS 513.88 and ATCC 1015^20,21^, we have characterized a total of 323 protease-like genes (Supplementary Table S2), including the 198 putative proteases reported by Pel et al*.*^18^ and 125 extra putative proteases found by homology search, which is similar to the total number of proteases identified by Budak et al*.* in *A. niger* and other *Aspergillus* species^17^. However, only 232 of them were provisionally identified as proteases by further manual annotation steps, which cover 1.64% of the 14,165 protein-coding genes predicted from the *A. niger* genome^18^. Another 91 genes were shown to be non-peptidase homologues, among which 65 genes were previously predicted as proteases by Pel et al*.*^18^. Among the 307 known and putative *A. niger* peptidases deposited in version 12.2 MEROPS database (https://www.ebi.ac.uk/merops/cgi-bin/speccards?sp=sp000086;type=peptidase), only 186 putative peptidases are identified as proteases in this study, while 55 peptidases are identical to others, with other 66 proteins reclassified as non-peptidase homologues (Supplementary Table S3). Interestingly, besides the 186 putative proteases in MEROPS database, another 46 putative peptidase were identified in the present study.

Among the 232 putative proteases identified here, 216 proteases had homologues in the other *A. niger* strain, while eleven and five proteases were only from *A. niger* strains CBS 513.88 and ATCC 1015, respectively, containing only one single member with no homologues in the other strain (Supplementary Table S2), and were therefore considered “orphan genes”^22^. As analyzed by the six subcellular location predictors, 62 out of the 232 proteases (i.e., 26.7% of the whole degradome) were found to be extracellular enzymes, which was similar to the number of extracellular proteases in *A. flavus* NRLL 3357 but more than those in other *Aspergillus* species^17^.

### Reclassification of the proteases identified in the two *A. niger* strains

**Figure 1:**
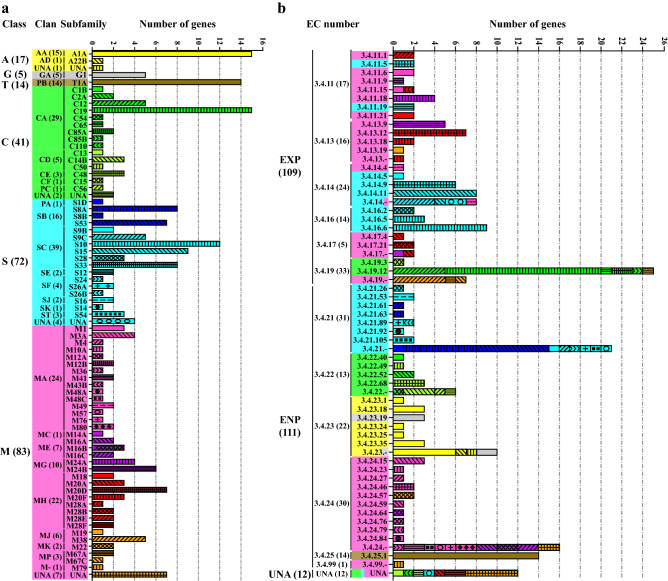
Reclassification of the putative proteases from *A. niger* CBS 513.88 and ATCC 1015. (**a**) Assignment of the proteases according to their substrate specificities, cleavage sites and active sites. (**b**) Classification of the proteases based on their EC numbers; proteases in Fig. 1b are highlighted with the same color and the same types of columns as the corresponding members in Fig. 1a; UNA, unassigned, peptidases that can’t be assigned into any clan or family; EXP, exopeptidases; ENP, endopeptidases; figures in parentheses indicate the number of proteases in each clan or subfamily.

Reassignment of the proteases was performed by the combination of MEROPS database (version 12.2) and manual literature searches. Finally, based on the cleavage sites and active sites, the 232 putative *A. niger* proteases were reassigned into 6 classes, including ~ 7.3% (17/232) aspartic, ~ 2.2% (5/232) glutamic, ~ 6.0% (14/232) threonine, ~ 17.7% (41/232) cysteine, ~ 31.0% (72/232) serine, and ~ 35.8% (83/232) metallopeptidases (Table 1, Fig. 1a and Supplementary Table S2). Thus, metallopeptidases are the most abundant proteolytic enzymes in *A. niger*, inconsistent with the previous finding that serine proteases are the largest group across *Aspergillus* species^17^. Within each catalytic class, the number of members per subfamily is highly variable, ranging from 1 to 15 (Fig. 1a, Supplementary Table S2). Several subfamilies contain a large number of representatives, e.g. the A1A, T1A, C19 and S10 subfamilies comprise of 15, 14, 15 and 12 members, respectively, which totally accounts for 24.1% of the whole *A. niger* degradome and are in disagreement with the major components of the human degradome^6^. In contrast, there are some subfamilies containing one single member in the *A. niger* genome (i.e., A22B, C13, S1D and M4). The overall abundance and diversity may reflect the various highly specialized roles these enzymes play. These proteases were further assigned into 25 annotated and one non-classified clans, and 71 subfamilies, with one aspartic, two cysteine, four serine and seven metallopeptidases unassigned into any clan or family (Table 1 and Fig. 1a).

**Table 1:** Summary of the main characteristics of the putative proteases annotated from the *A. niger* genome.

	Catalytic class						Total
	Aspartic	Glutamic	Threonine	Cysteine	Serine	Metallo	
No. gene models	17	5	14	41	72	83	232
No. protease clans	2	1	1	5	8	9	26
No. protease subfamilies	2	1	1	15	17	35	71
Unassigned proteases ^a^	1	0	0	2	4	7	14
New members ^b^	6	5	6	21	34	30	102
Proteases with new active sites ^c^	4	0	4	15	36	51	110
Putatively inactive proteases ^d^	2	0	4	1	1	4	12
Secreted proteases ^e^	11	4	0	2	32	13	62
Molecularly and/or biochemically characterized proteases	6	1	0	0	11	1	19

^a^Number of peptidases that can not be assigned into any clan or family based on current classification system.

^b^New members are proteases newly assigned into the class or proteases whose catalytic classes are different from those identified in the literature. For more details, see protease classes colored in red in Supplementary Table S2.

^c^Proteases with new active sites represent proteases that have different active sites and/or metal binding residues from those deposited in version 12.2 MEROPS database. For more details, see the active sites and/or metal binding residues colored in red in Supplementary Table S2.

^d^Based on the absence of consensus active site residues in the amino acid sequences of the proteases.

^e^Based on the analyses of the six subcellular location predictors.

A total of 102 new members have been reassigned into the six protease classes, including 6 aspartic, 5 glutamic, 6 threonine, 21 cysteine, 34 serine and 30 metallopeptidases (Table 1). There were also 4, 0, 4, 15, 36 and 51 proteases with new active sites and/or metal binding residues from aspartic, glutamic, threonine, cysteine, serine and metallopeptidase classes, respectively. However, 2 aspartic, 4 threonine, 1 cysteine, 1 seirne and 4 metallopeptidases are found to be putatively inactive enzymes. The 62 putatively secreted proteases belong to all catalytic classes, except threonine proteases, consistent with the observation in *Meloidogyne incognita* genome^7^. Computer-based comprehensive literature searches showed that a total of 19 proteases have been molecularly and/or biochemically characterized, among which there are 6 aspartic, 1 glutamic, 11 serine and 1 metallopeptidases (Supplementary Table S4). Besides, 41 proteases have been characterized by genomic, transcriptomic, proteomic or secretomic techniques.

The proteases identified were also classified based on their EC numbers. As shown in Fig. 1b, the 232 proteases were mainly composed of 109 exopeptidases, 111 endopeptidases, and 12 proteases with unknown EC numbers. The exopeptidases were further assigned into seven sub-subfamilies, including 17 aminopeptidases (EC 3.4.11), 16 dipeptidases (EC 3.4.13), 24 dipeptidyl- and tripeptidyl-peptidases (EC 3.4.14), 14 serine-type carboxypeptidases (EC 3.4.16), 5 metallocarboxypeptidases (EC 3.4.17) and 33 omega peptidases (EC 3.4.19), whereas endopeptidases comprise 31 serine endopeptidases (EC 3.4.21), 13 cysteine endopeptidases (EC 3.4.22), 22 aspartic endopeptidases (EC 3.4.23), 30 metalloendopeptidases (EC 3.4.24), 14 threonine endopeptidases (EC 3.4.25), and 1 endopeptidase with unknown catalytic mechanism (EC 3.4.99).

As can also be seen from Fig. 1, the aspartic, glutamic and threonine peptidases identified in *A. niger* genome were all endopeptidases, while cysteine, serine and metallopeptidase classes contained both exo- and endopeptidases. Cysteine proteases were composed of omega peptidases and cysteine endopeptidases. Inconsistent with the previous observation that most serine peptidases are endopeptidases^6^, serine peptidases identified in *A. niger* were mainly exopeptidases, including aminopeptidases, dipeptidyl- and tripeptidyl-peptidases, and serine-type carboxypeptidases, with only 31 endopeptidases. Metallopeptidases were made up of 13 aminopeptidases, 16 dipeptidases, 5 metallocarboxypeptidases, 8 omega peptidases, 31 metalloendopeptidases, and 2 dipeptidyl peptidases.

## Subdivision, probable physiological functions and active site architectures of the six classes of *A. niger* proteases

### Aspartic proteases

Aspartic proteases, also called aspartate, aspartyl or acid proteases (EC 3.4.23) are distributed across all forms of life, including viruses, bacteria, fungi, plants, protozoa and animals. In microorganisms, they perform important functions related to nutrition and pathogenesis, and have characteristics that make them attractive for industrial applications^1,23,24^.

Aspartic proteases are currently divided into 5 clans and 16 families in MEROPS database^5^. The *A. niger* genome encodes 17 putative aspartic peptidases from 2 clans, clan AA, which contains 15 enzymes from subfamily A1A, and clan AD, which has one single enzyme from subfamily A22B, with one enzyme (An12g02180) unassigned into any clan and subfamily (Table 2, Supplementary Table S2 and Supplementary Fig. S1a). Subfamily A1A is typified by pepsin, a digestive enzyme optimally active at an acidic pH^25^. So far, 6 enzymes from subfamily A1A have been molecularly and/or biochemically characterized, including An15g06280, An01g00370, An12g03300, An04g01440, An14g04710 and An02g07210 (Supplementary Table S3). Among them, An01g00370, An14g04710 and An15g06280 have been identified as aspergillopepsin I^26,27^. Besides, 53364, An13g02130 and An18g01320 are provisionally identified as barrierpepsin, while An02g07210, An04g01440 and An07g00950 are putative saccharopepsin, pepsin A, and candidapepsin, respectively. In non-pathogenic fungi (i.e., *A. niger*), these enzymes are involved in a diverse range of processes like digestion^27,28^. Subfamily A22B is typified by intramembrane protease aspartic protein (IMPAS)-1. An15g02400 in this subfamily is provisionally identified as the signal peptide peptidase which is a multi-pass transmembrane aspartic protease that cleaves type 2 membrane proteins^29^.

**Table 2:** Aspartic proteases encoded by *A. niger* strains CBS 513.88 and ATCC 1015.

Clan	Subfamily	Archetype	Provisional ID ^a^	Old locus tag ^b^	Conserved catalytic motif ^c^
AA	A1A	Pepsin A	Barrierpepsin	53364*	DTG, DSG
				An13g02130*	DTG, DTG
				An18g01320*	DTG, DSG
			Aspergillopepsin I	**An01g00370**	DTG, DTG
				An14g04710*	DTG, DTG
				**An15g06280**	DTG, DTG
			Saccharopepsin	An02g07210*	DTG, DTG
			Pepsin A	An04g01440*	DTG, DTG
			Candidapepsin	An07g00950*	DTG, DTG
			/	An11g00310*	DWT, DHA
			/	An11g09170*	DLT
			/	An12g03300*	DTG, DSG
			/	An15g07770	DRG
			/	An11g09240*	
			/	An11g10270	
AD	A22B	Intramembrane protease aspartic protein-1	Signal peptide peptidase	An15g02400	YD, GLGD

^a^ /, not provisionally identified.

^b^ Proteases that have been molecularly and/or biochemically characterized are in bold.

^c^ The putative catalytic residues are colored in pink.

* Secreted proteases that have been predicted by the six predictors.

Eleven enzymes from subfamily A1A contain the hallmark active site motif Asp-Thr-Gly (I) and its accompanying motif, Gly-hydrophobic-hydrophobic-Gly (II), to form the first psi loop. The sequences Asp-Thr/Ser-Gly (III) and Ile-hydrophobic-Gly-Asp-/Gln/Asn (IV) form the second psi loop (Table 2, Supplementary Fig. S2a). Their N-terminal domains also possess the strictly conserved Tyr residues which are located in a β-hairpin loop overhanging the active sites^28^. An11g09170 and An15g07770 have only one conserved catalytic Asp residue (Asp81 and Asp45, respectively), whereas An11g09240 and An11g10270 don’t contain the conserved active sites of the aspartic proteases family, and may therefore not be enzymatically active. As the typical subfamily A22B signal peptide peptidase, An15g02400 contains two conserved active site motifs YD and GXGD (X represents any amino acid) in adjacent membrane-spanning domain, and a conserved PAL motif of unknown function near its C terminus (Supplementary Fig. S2a)^29^.

### Glutamic proteases

Glutamic protease (EC 3.4.23.19), previously known as the A4 family of aspartic endopeptidases, has been reclassified as a new catalytic type of peptidase (family G1) in the version 12.2 MEROPS database^5^. It is also called the Eqolisin, a name derived from the active-site residues, glutamic acid (E) and glutamine (Q), which activate the nucleophilic water and stabilize the tetrahedral intermediate on the hydrolytic pathway, respectively^30^. Glutamic proteases are quite distinct from previously characterized proteases due to the fact that their distribution is limited to filamentous fungi^30^, because they are essential for fungal growth in protein medium at low pH^31^.

The MEROPS database (version 12.2) lists 2 glutamic protease families, which are assigned to 2 clans, and one unassigned family^5^. Examination of the genomes of *A. niger* CBS 513.88 and ATCC 1015 showed that they contained 5 glutamic proteases belonging to clan GA and family G1 (Table 3, Supplementary Fig. S1b), which was more than that was previously reported^30^. Of these 5 glutamic proteases, An01g00530 has been identified as aspergillopepsin II^32–34^ which can decolorize hemoglobin and improve animal performance^35,36^. An07g00320 and An15g07700 show high similarity to An01g00530 and are also provisionally identified as aspergillopepsin II. Multiple sequence alignment analysis shows a high level of conservation of the active site residues Gln and Glu in these 5 glutamic proteases (Supplementary Fig. S2b). In addition, An01g00530 and An15g07700 are seen to specify the conserved cysteine residues that form a disulphide bridge (Supplementary Fig. S2b), which surrounds an aspartic acid residue that is conserved in all members of this family^30^.

**Table 3:** Glutamic proteases encoded by *A. niger* strains CBS 513.88 and ATCC 1015.

Clan	Subfamily	Archetype	Provisional ID ^a^	Old locus tag ^b^	Catalytic residues
GA	G1	Scytalidoglutamic peptidase	Aspergillopepsin II	An01g00530*	Gln, Glu
				An07g00320	
				An15g07700*	
			/	126,639*	
			/	An14g03250*	

^a^ /, not provisionally identified;

^b^ Proteases that have been molecularly and/or biochemically characterized are in bold;

* Secreted proteases that have been predicted by the six predictors.

### Threonine proteases

As a new class of endopeptidases, threonine proteases, which are contained within clan PB, are so called because they utilize the N-terminal threonine as the active site. They are mostly known for their roles as the major components of the catalytic subunits of eukaryotic proteasomes, which are part of the protein turnover system^37^. Threonine proteases are currently classified into 6 families, T1, T2, T3, T5, T7 and T8^5^. The *A. niger* genome encodes 10 putatively active and 4 putatively inactive threonine peptidases from subfamily T1A for which the type example is beta component of archaean proteasome (Supplementary Table S2 and Supplementary Fig. S1c). These proteases are provisionally identified as 5 proteasome α subunits and 6 proteasome β subunits (Table 4), enzymes that are involved in the protein turnover housekeeping mechanism in the cell^37^. Besides, there are also 3 subfamily T1A peptidases that can not be currently identified.

**Table 4:** Threonine proteases encoded by *A. niger* strains CBS 513.88 and ATCC 1015.

Clan	Subfamily	Archetype	Provisional ID ^a^	Old locus tag	Catalytic residue
PB	T1A	Archaean proteasome, beta component	Proteasome subunit alpha 5	An02g03400	Thr
			Proteasome subunit alpha 6	An02g07040	
			Proteasome subunit alpha 2	An07g02010	
			Proteasome subunit alpha 4	An11g06720	
			Proteasome subunit beta 5c	An11g01760	
			Proteasome subunit beta 2c	An11g04620	
			Proteasome subunit beta 1c	An13g01210	
			/	An02g10790	
			/	An18g06680	
			/	An18g06800	
			Proteasome subunit beta 3	An04g01800	
			Proteasome subunit beta 2	An04g01870	
			Proteasome subunit alpha 1	An15g00510	
			20S proteasome component beta 6	An18g06700	

^a^ /, not provisionally identified.

All these 14 threonine proteases from *A. niger* have not been previously characterized by molecular, biochemical and/or omics techniques. They contain the active-site nucleophile Thr (Supplementary Fig. S2c)^38,39^, with four exceptions (An15g00510, An18g06700, An04g01800 and An04g01870) which are therefore unlikely to possess proteolytic activity. These threonine proteases possess the conserved motifs GXXXD and GXD (X represents any amino acid). Besides, within the amino acid sequences of An13g01210 and An11g01760, the two glycine-rich sequences (GSG and SGG) are also conserved and in direct proximity to their active-site residues (Supplementary Fig. S2c). Except An02g07040, An18g06800 and An18g06680, all other threonine proteases contain the proton acceptor lysine residues (Supplementary Fig. S2c).

### Cysteine proteases

Cysteine proteases, also called cysteine peptidases, depend on a cysteine residue for activity^40^. They regulate multiple biological functions, including protein turnover, protein quality control, cell death, proliferation, extracellular matrix turnover and surface proteins processing^41–43^. The version 12.2 MEROPS database lists 97 families of cysteine peptidases, among which 81 families are assigned into 15 annotated clans, while the rest 12 families belonging to unassigned clans^5^. The *A. niger* genome putatively encodes at least 38 active and 1 inactive (39581) cysteine proteases from 15 subfamilies clustered in 5 clans: CA, CD, CE, CF and PC, with two proteins unassigned into any clan and family (An07g03830 and An08g09840, Table 5, Supplementary Table S2 and Supplementary Fig. S1d). However, none of these cysteine proteases has been molecularly and/or biochemically characterized. Consistent with the previous finding that CA is the largest cysteine peptidases clan^40^, 28 *A. niger* cysteine proteases are from clan CA, comprising families/subfamilies C1B, C2A, C12, C19, C110, C54, C65, C85A and C85B (Table 5, Supplementary Fig. S1d). Clan CD consists of 5 sequences from subfamilies C13, C14B and C50, while clans CE, CF and PC contain 3, 1 and 1 members from families C48, C15 and C56, respectively. Given that the large number of *A. niger* cysteine proteases, we have split discussions below based on clans for simplicity and clarity.

**Table 5:** Cysteine proteases encoded by *A. niger* strains CBS 513.88 and ATCC 1015.

Clan	Subfamily	Archetype	Provisional ID ^a^	Old locus tag	Conserved cysteine motif ^b^	Arrangement of catalytic residues
CA	C1B	Bleomycin hydrolase	Bleomycin hydrolase	An01g01720	QXXXXXC	Cys, His, Asn
	C2A	Calpain-2	Calpain	An01g04680	QXXXXXC	
			Calpain	An11g02950	QXXXXXXC	
	C12	Ubiquitinyl hydrolase-L1	Uch2 peptidase	An01g11160	QXXXXXC	Cys, His, Asp
			Ubiquitinyl hydrolase-YUH1	An02g13920		
			/	An12g01820		
			/	An08g11630		
			Ubiquitinyl hydrolase-YUH1	An11g11130		Cys, His, Glu
	C19	Ubiquitin-specific peptidase 14	/	An02g07490	NXXXXC	Cys, His, Ser
			Ubp14 peptidase	An02g14990		Cys, His, Asn
			Ubp8 peptidase	An06g01920		
			/	An02g01420		
			/	An06g01380		
			/	An12g03700		
			/	An12g08370		
			Ubp3 peptidase	An07g09730		Cys, His, Asp
			Ubp15 peptidase	An09g05480		
			/	An01g08470		
			/	An07g10130		
			/	An11g04380		
			/	An14g05150		
			/	An17g01260	NXXXXXC	
			/	39,581		His, Asp
	C110	Kyphoscoliosis peptidase	Kyphoscoliosis peptidase	An11g01080		Cys, His, Asp
	C54	Autophagin-1	ATG4 peptidase	An11g11320	DXXXXC	Cys, Asp, His
	C65	Otubain-1	Otubain-1	39,420	DXXC	Asp, Cys, His
	C85A	OTLD1 deubiquitinylating enzyme	OTU2 peptidase	An01g02440		
				An01g09320		
	C85B	OTU1 peptidase	YOD1 peptidase	An11g11090		
CD	C13	Legumain	Glycosylphosphatidylinositol: protein transamidase	An01g13530*	HGX_39_TC	His, Cys
	C14B	Metacaspase Yca1	Metacaspase Yca1	An09g04470	HGX_53_SC	
				An18g05760		
			/	An11g05400	HGX_41_AC	
	C50	Separase	Separase	An07g03090	HGX_23_GC	
CE	C48	Ulp1 peptidase	Ulp1 peptidase	An09g05400	QXXXXXC	His, Asp, Cys
				An13g01190		
				An14g05500		
CF	C15	Pyroglutamyl-peptidase I	Pyroglutamyl-peptidase I	An11g01970	DXXXXXC	Glu, Cys, His
PC	C56	PfpI peptidase	/	An16g00930		Cys, His, Glu

^a^ /, not provisionally identified;

^b^ The putative catalytic residues are colored in pink. X represents any amino acid;

* Secreted proteases that have been predicted by the six predictors, the unassigned extracellular cysteine protease An08g09840 is not presented in this table.

### Clan CA

Within clan CA, C19 is the largest family with 15 members followed by family C12, which contains 5 enzymes (Fig. 1a, Table 5 and Supplementary Table S2). Both subfamilies C2A and C85A possess 2 enzymes, while subfamilies C1B, C54, C65, C85B and C110 contain only one member (Table 5). Most of these cysteine peptidases are putative housekeeping enzymes involved in the autophagy^44^ and ubiquitin cellular homeostasis regulatory mechanisms which regulates protein turnover^45,46^. The only sequence in family C54, An11g11320, is putatively identified as the autophagy related protein 4 which is part of the core molecular machinery of the autophagy system^44,47^. Protein ubiquitination is a reversible process which starts with ubiquitin being attached to target proteins by ubiquitination enzymes and ends with deubiquitinating enzymes (DUBs) releasing ubiquitin to be cycled^46^. In the *A. niger* genome, three different families of DUBs have been identified: 4 out of the 15 cysteine peptidases from family C19 (An02g14990, An06g01920, An07g09730 and An09g05480) have been provisionally identified as ubiquitin-specific proteases (USPs/UBPs), while An01g11160, An02g13920 and An12g01820 from family C12 are putative ubiquitin C-terminal hydrolases (UCHs). Moreover, the 3 sequences in family C85 (An01g02440, An01g09320 and An11g11090) and the lone enzyme sequence in family C65 (39420) are ovarian-tumor (OTU) domain DUBs. Other 2 enzymes from family C12 (An08g11630 and An11g11130) and 10 proteases from family C19 have not been provisionally identified. Besides, 39581 from family C19 doesn’t contain the catalytic cysteine residue and may be enzymatically inactive.

The single enzyme in subfamily C1B (An01g01720) is provisionally identified as bleomycin hydrolase, an enzyme participated in antigen presentation and bleomycin chemotherapy resistance^48^. The two enzymes in subfamily C2A, An01g04680 and An11g02950, are calcium dependent intracellular cysteine peptidases (also called calpain) which serve as modulator proteases of the pH adaption system in *A. nidulans*^49,50^. An11g01080 from family C110 is a putative kyphoscoliosis peptidase that plays an important role in muscle growth in mammals^51^, but its physiological function in microorganisms remains to be elucidated.

Cysteine proteases usually contain a Cys/His/Asn triad at the active site, where the nucleophilic cysteine residue attacks the carbon of the reactive peptide bond and histidine residue acts as a proton donor and enhances the nucleophilicity of the cysteine residue^41^. Apart from Asn, the third residue can also be Asp, Ser, or Glu (Table 5), the side chain of which seems to orient the side chain of the histidine favourably for catalysis^40^. Most clan CA cysteine peptidases have similar arrangement of catalytic residues: Cys-His-(Asn/Asp/Glu/Ser), except those from subfamilies C54, C65, C85A and C85B which contain Asp in different orders (Table 5). In cysteine peptidases from clan CA, the catalytic cysteine residue often occurs in the following conserved motifs: DXXC, (N/D)XXXXC, (N/Q)XXXXXC or QXXXXXXC (X represents any amino acid; Table 5, Supplementary Fig. S2d), where N or Q is the oxyanion hole residue^52^.

### Clan CD

The *A. niger* genome encodes 5 putatively active clan CD enzyme sequences from families/subfamilies C13, C14B and C50 (Table 5, Supplementary Table S2 and Supplementary Fig. S1d). An01g13530 in family C13 is provisionally identified as glycosylphosphatidylinositol (GPI) protein transamidase, an enzyme attaching GPI anchors to proteins as they enter the lumen of the endoplasmic reticulum^53^. Of the 3 subfamily C14B cysteine peptidases, An09g04470 and An18g05760 are putative metacaspases which play vital roles in cell death, stress and cell proliferation^42,43^, whereas An11g05400 has not been provisionally identified. In family C50, An07g03090 is provisionally identified as separase/separin, an enzyme involved in the cleavage of cohesion and hydrolysis of endrin (also called pericentrin)^54,55^, which plays important roles in cell replication.

Like caspases^40^, the catalytic cysteine of clan CD cysteine proteases from *A. niger* usually present in the motif: HisGly-[spacer]-(Ala/Gly/Ser/Thr)Cys, where the spacer is composed of 23–53 amino acids as shown in Table 5 and Supplementary Fig. S2d. A putative His/Cys dyad is suggested to participate in the catalytic mechanism of these enzymes, in the reverse order to those in clan CA.

### Clan CE

The MEROPS database (version 12.2) lists 7 families in this clan, C5, C48, C55, C57, C63, C79 and C122^5^. There are 3 putatively active clan CE cysteine peptidases from family C48 in the *A. niger* genome (Table 5, Supplementary Table S2), An09g05400, An13g01190 and An14g05500, which are all provisionally identified as ubiquitin-like protein 1 (Ulp1) peptidases, also known as sentrin-specific proteases (SENP) or small ubiquitin-related modifier (SUMO) endopeptidases^56^. Like ubiquitinylation, SUMOylation, the covalent attachment of the SUMO proteins to target proteins, regulates the function of a large variety of cellular proteins via post-translational modification and plays a vital role in numerous biological processes, such as intracellular transport, gene expression, protein stability, and genomic and chromosomal integrity^57^.

Clan CE is another example using the His/Cys order in the catalytic residues, but it also contains Asp between His and Cys as the third catalytic residue (Table 5, Supplementary Fig. S2d). In clan CE cysteine peptidases, the catalytic cysteine residues occur in the motif QXXXXXC, where X denotes any amino acid.

### Clan CF

The *A. niger* encodes one putatively active clan CF cysteine protease from family C15 (An11g01970, Table 5 and Supplementary Table S2), which is a putative pyroglutamyl peptidase I (also known as pyrrolidone carboxyl peptidase, EC 3.4.19.3). Although its exact function in *A. niger* remains to be elucidated, this enzyme has been proposed to reduce toxicity of N-terminally blocked peptides or play a role in nutrient assimilation in *Thermococcus litoralis*^58^. An11g01970 contains the catalytic triad Glu-Cys-His, with the catalytic cysteine occurring in the motif DXXXXXC (Supplementary Fig. S2d).

### Clan PC

The *A. niger* genome contains only one enzyme (An16g00930, Table 5) from family C56 in clan PC, which is typified by *Pyrococcus furiosus* protease I (PfpI). PfpI has homologues in nearly every organism and cell, ranging from *Escherichia coli* to *Homo sapiens*. Although its function remains unclear, the ubiquity and evolutionary conservation of PfpI suggest that it may play a fundamental physiological role^59^. Although An16g00930 has not been provisionally identified, it is shown to use the catalytic triad Cys/His/Glu.

### Serine proteases

Serine proteases are characterized by the presence of three critical amino acids Ser/His/Asp in their active sites, where serine and histidine are the nucleophile, and general base and acid, respectively, while the aspartate helps orient the histidine residue and neutralize the charge that develops on the histidine during the transition states^6,60^. These proteases are widely distributed in nature and found in all kingdoms of cellular life as well as many viral genomes. They play crucial roles in a wide variety of physiological functions, including protein digestion and processing, cell signaling, blood clotting, and inflammation^60,61^.

At the time of writing this manuscript, the version 12.2 MEROPS database has listed 54 serine protease families, of which 46 families are from 15 defined clans and the rest from unassigned clans. The *A. niger* genome encodes 72 putatively active serine proteases from 8 clans and 17 subfamilies, with 4 serine proteases from unassigned clans and subfamilies (Table 6, Supplementary Table S2 and Supplementary Fig. S1e). These 72 putatively active serine peptidases represent ~ 31.0% (72/232) of the *A. niger* degradome, in line with the previous findings in other organisms that serine proteases comprise nearly one-third of the degradome^62,63^. Among them, 16 subfamilies are from 7 clans (SB, SC, SE, SF, SJ, SK and ST) that contain serine peptidases only, while only one subfamily is from the mixed clan PA. Intriguingly, the 5 serine peptidase clans (PA, SC, SJ, SK and ST) which are usually present in nearly all forms of life^6^ have also been identified in *A. niger*. Given the huge expansion of serine peptidase families, we have developed narratives on these enzymes based on specific clans encoded by the *A. niger* genome.

**Table 6:** Serine proteases encoded by *A. niger* strains CBS 513.88 and ATCC 1015.

Clan	Subfamily	Archetype	Provisional ID ^a^	Old locus tag ^b^	Conserved serine motif ^c^	Arrangement of catalytic residues
PA	S1D	Lysyl endopeptidase	Nma111 peptidase	An08g08670	GXSXS	His, Asp, Ser
SB	S8A	Subtilisin Carlsberg	/	An02g02850	D(D/T)G、GXSXX(T/S/G)	Asp, His, Ser
			/	An07g03880*		
			Oryzin	An09g03780*		
			/	An14g01380		
			/	An14g01530		
			/	An16g06260*		
			/	An18g02630		
			/	An18g04970		
	S8B	Kexin	Kexin	An01g08530*		
	S53	Sedolisin	Aorsin	An01g01750*	EXXXD、GXSXXXP	Glu, Asp, Ser
				An03g01010*		
				An11g01110*		
			Grifolisin	An06g00190*		
				An08g04640*		
				An14g02470*		
				An16g02250*		
SC	S9B	Dipeptidyl-peptidase IV	/	An01g01210	GXSXG	Ser, Asp, His
			Dipeptidyl-peptidase IV	**An02g11420**		
	S9C	Acylaminoacyl-peptidase	Aminopeptidase C	**An04g02850**		
			/	An09g02830		
			/	An13g03240		
			Dipeptidyl-peptidase 5	An12g04700		
				An16g08150		
	S10	Carboxypeptidase Y	Carboxypeptidase C	An05g01870*	GESYA	
				An08g08750*		
				An11g06350*		
			Carboxypeptidase D	An02g04690*		
				An05g02170*		
				An07g08030*		
				An08g00430*		
				An14g02150*		
				An03g05200*	TESTG	
				An16g09010*		
				An17g00760*		
				An06g00310*	AESYG	
	S15	Xaa-Pro dipeptidyl-peptidase	/	An03g03810	GXSXXA	
			Xaa-Pro dipeptidyl-peptidase	131,499		
				An02g01000		
				An04g00980		
				An04g03100		
				An07g01430		
				An13g02260	GXSXXG	
				An16g06560	GXSXXS	
				An16g07710	DXSXXG	
	S28	Lysosomal Pro-Xaa carboxypeptidase	Acid prolyl endopeptidase	An08g04490*	GXSX(A/S)	
			Lysosomal Pro-Xaa carboxypeptidase	An12g05960*		
				An14g01120*		
	S33	Prolyl aminopeptidase	Tripeptidyl-peptidase I	An03g02530	GXSXG	
				An09g02370*		
				An09g03800		
				An12g08560		
				An13g02620*		
				An13g02790*		
			Prolyl aminopeptidase	**An11g04730**		
				An16g06070		
SE	S12	D-Ala-D-Ala carboxypeptidase B	D-stereospecific aminopeptidase	An09g00950	SXXK	Ser, Lys, Tyr
				An16g06750		
SF	S24	Repressor LexA	/	An02g06070	GXSXE	Ser, Lys
	S26A	Signal peptidase I	Signal peptidase I	An09g02730	GXSX(T/Y)	
			/	An14g06320		
	S26B	Signalase 21 kDa component	Signal peptidase I	An01g00560		Ser, His
SJ	S16	Lon-A peptidase	Endopeptidase La	An02g03760	GXSXG	Ser, Lys
				An18g02980		
SK	S14	Peptidase Clp (type 1)	Endoeptidase Clp	An02g11960	AXSXG	Ser, His, Asp
ST	S54	Rhomboid-1	Rhomboid protease	An08g00670	GXSG、AHXXGXXXG	Ser, His
				An08g10730		
			/	An15g06920		

^a^ /, not provisionally identified;

^b^ Proteases that have been molecularly and/or biochemically characterized are in bold;

^c^ The putative catalytic residues are colored in pink; X represents any amino acid;

* Secreted proteases that have been predicted by the six predictors, the unassigned extracellular serine proteases An02g01550 and An07g00580 are not listed in the present table.

### Clan PA

The MEROPS database (version 12.2) lists a mixture of 9 cysteine and 14 serine peptidase families in clan PA^5^. Within this clan, *A. niger* genome encodes one putatively active enzyme (An08g08670) from subfamily S1D which is typified by lysyl endopeptidase, inconsistent with the previous observation that expansion of the clan PA peptidases occurs only in the higher organisms^6^. An08g08670 has been provisionally identified as Nma111 peptidase which mediates apoptosis through proteolysis of the apoptotic inhibitor BIR1^64^.

Serine peptidases from clans PA, SB, SC and SK use a Ser/His/Asp catalytic triad, where these three residues are ordered differently in the polypeptide sequences, and the Ser/Lys dyad falls within four clans (SE, SF, SJ and SR), while Ser/His proteases fall within clan ST (Table 6, Supplementary Table S2). The catalytic serine residue of serine peptidases usually present in the motif GXSXG, where X denotes any amino acid. Like other serine proteases in clan PA^61^, An08g08670 also utilizes a catalytic triad in the order of His/Asp/Ser, with the catalytic serine contained in the motif GXSXS (Table 6, Supplementary Fig. S2e).

### Clan SB

Consistent with the observation that clans SB and SC are the dominant components of serine peptidases in archaea, prokaryotes, fungi and plants, *A. niger* genome encodes 16 and 39 serine peptidases from clans SB and SC, respectively, which cover ~ 76.4% (55/72) of all the *A. niger* serine peptidases. Besides, most clan SB peptidases in *A. niger* genome are extracellular enzymes, in line with the previous finding that most clan SB peptidases are localized to the cell membrane or secreted outside of the cell^6^. Clan SB contains serine peptidases from 2 families: S8 typified by subtilisin in subfamily S8A or kexin in subfamily S8B, and the S53 family typified by sedolisin^5^. The *A. niger* genome respectively contains 9 and 7 putatively active endopeptidases from families S8 and 53 (Table 6). Of the enzymes from family S8, An09g03780 and An01g08530 have been molecularly and biochemically identified as oryzin^65^ and kexin^66,67^, respectively. Oryzin is an alkaline protease that hydrolyzes proteins with broad specificity, while kexin is a calcium-dependent, neutral serine peptidase involved in proprotein-processing along the secretion pathway^66^. Among the enzymes from family S53, An01g01750, An03g01010 and An11g01110 are provisionally identified as aorsins which have trypsin-like specificity at acidic pH^68^, while An06g00190, An08g04640, An14g02470 and An16g02250 are putative grifolisins, the pepstatin-insensitive carboxyl proteases^69^.

Most clan SB peptidases use residues serine, aspirate, and histidine ordered differently in the polypeptide sequences as the catalytic triads (Table 6, Supplementary Fig. S2e). Serine peptidases from family S8 use a Asp/His/Ser catalytic triad mechanism, where Asp and Ser occur in motifs D(D/T)G and GXSXX(T/S/G), respectively. But members of family S53 utilize a novel Glu/Asp/Ser triad, where Glu and Ser are located in the conserved motifs EXXXD and GXSXXXP, respectively.

### Clan SC

Clan SC, which is particularly important in cell signaling mechanisms, is composed of 7 families, S9, S10, S15, S28, S33, S37 and S82^5^. As the largest serine peptidase clan in *A. niger*, clan SC comprises 39 putatively active enzymes, including 7, 12, 9, 3 and 8 peptidases from families S9, S10, S15, S28 and S33, respectively (Table 6, Supplementary Table S2). Clan SC serine peptidases in *A. niger* posses both endoproteolytic and exoproteolytic activities, which contrasts the trend in other serine peptidase clans in which members have predominantly one or the other activity. One serine peptidase (An02g11420) from subfamily S9B has been identified as dipeptidyl-peptidase IV, an enzyme involved in the proteolytic maturation of enzymes produced by *A. niger*^70^. An04g02850 from subfamily S9C, a previously identified phenylalanine aminopeptidase^71^, is predicted to be dipeptidyl aminopeptidase in the present study, and further studies are therefore needed to identify its exact function. Other two enzymes in subfamily S9C, An12g04700 and An16g08150, are provisionally identified as dipeptidyl-peptidase 5 which releases dipeptides Ala-Ala, Lys-Ala, His-Ser, Ser-Tyr, and Gly-Phe from chromogenic peptide substrates^72^. Of the 12 serine peptidases from family S10, An05g01870, An08g08750 and An11g06350 are provisionally identified as carboxypeptidase C, whereas other 9 enzymes are predicted to be carboxypeptidase D. These serine-type carboxypeptidases degrade exogenous proteins as a nitrogen source^73^ and have wide applications in peptide synthesis and amino acid sequencing^74^. Except for An03g03810, other 8 enzymes from family S15 are provisionally identified as Xaa-Pro dipeptidyl-peptidases which probably participate in the degradation of caseins^75,76^. In family S28, An08g04490 has been identified as prolyl oligopeptidase (also called prolyl endopeptidase) which cleaves the internal proline residues of proline-rich oligopeptides or proteins^77,78^, and has been used in the debittering of protein hydrolysates^79^ as well as food protein hydrolysis^80^. An12g05960 and An14g01120 are putative lysosomal Pro-Xaa carboxypeptidases which function in blood pressure regulation, tissue proliferation and smooth-muscle growth in human^81^. Among the 8 putatively active enzymes from family S33, An11g04730 and An16g06070 are identified as prolyl aminopeptidases which have been extensively used in flavour development of food products^82^, while other 6 enzymes are predicted to be tripeptidyl-peptidase I that cleaves peptide hormones and processes specific peptides^83^.

Clan SC peptidases are α/β hydrolase-fold enzymes consisting of parallel β-strands surrounded by α-helices^6^. All the clan SC peptidases carry out catalysis using the Ser/Asp/His triad, with the catalytic serine residues of enzymes from families S9, S10, S28 and S33 present in the motif (T/A/G)XSX(G/A/S) (Table 6, Supplementary Fig. S2e). Although serine proteases from family S15 contain the conserved motif GXSXG, their catalytic serine residues occur in the motif (G/D)XSXX(G/A/S)^75,76^.

### Clan SE

Clan SE peptidases play important roles in bacterial cell wall metabolism with a minimal distribution in higher microorganisms. The MEROPS database lists 3 families in clan SE, S11, S12 and S13^5^. The *A. niger* genome encodes 2 putatively active enzymes (An09g00950 and An16g06750) in family S12 (Table 6), and they have been provisionally identified as D-stereospecific aminopeptidases which hydrolyze a wide range of D-alanine derivatives^84^. Like other clan SE peptidases, family S12 peptidases from *A. niger* use a triad mechanism generated from the pairing of Ser and Lys separated by only two residues, and the third Tyr residue assisting abstraction of the proton from the nucleophilic Ser (Table 6, Supplementary Fig. S2e)^6^.

### Clan SF

Clan SF comprises 2 families, S24 and S26, which are typified by *E. coli* LexA and signal peptidase I, respectively^5^. There are two (An09g02730 and An14g06320) and one (An01g00560) putatively active enzymes in subfamilies S26A and S26B in the *A. niger* genome, respectivley. Among them, An09g02730 and An01g00560 are putative signal peptidase I enzymes which cleave secretory signal sequences from exported and periplasmic proteins^85^. These clan SF serine peptidases are the prototypic proteases that use a Ser/Lys dyad, with an exception of An01g00560 which utilizes a dyad of Ser/His (Table 6, Supplementary Fig. S2e). The catalytic serine residues of clan SF peptidases usually occur in the motif GXSX(T/Y).

### Clan SJ

This clan possesses unique ATP-dependent proteases from 3 families, S16, S50 and S69. The *A. niger* genome encodes 2 putatively active Lon-A peptidases (An02g03760 and An18g02980), which are involved in intracellular protein turnover of transient regulatory proteins and misfolded proteins, thus contributing to stress tolerance and biofilm formation^86^. Like clan SF peptidases, enzymes in this clan also employ the Ser/Lys dyad to broker catalysis, where the catalytic serine presents in the conserved motif GXSXG (Table 6, Supplementary Fig. S2e), but these two catalytic residues are far away from each other.

### Clan SK

This clan is composed of 3 families, S14, S41 and S49. The *A. niger* genome encodes a putatively active ATP-dependent caseinolytic protease (Clp, An02g11960) from family S14, which participates in protein quality control by removing damaged, misfolded and regulatory proteins^87^. An01g06080, which shares 24.12% identity with the ClpR variant of ClpP, however, lacks the catalytic triad of ClpP protease and may be proteolytically inactive^87,88^. ClpP peptidases use a conventional catalytic triad of residues but in a novel arrangement of Ser, His and then Asp in the polypeptide sequence, with the catalytic Ser contained in the motif AXSXG (Table 6, Supplementary Fig. S2e).

### Clan ST

This clan contains a single family of enzymes, S54 typified by rhomboid proteases, which are widely conserved in species ranging from bacteria to human^89^, and have diverse functions, such as intracellular signaling, mitochondrial morphology and dynamics, as well as quorum sensing^90^. The *A. niger* genome encodes 3 putatively active enzymes from family S54, among which An08g00670 and An08g10730 are provisionally identified as rhomboid proteases (Table 6). These enzymes use a catalytic dyad of Ser/His for proteolysis, with the catalytic residues serine and histidine present in the conserved motifs GXSG and AHXXGXXXG, respectively (Table 6, Supplementary Fig. S2e).

### Metallopeptidases

Metalloproteases contain both endo- and exopeptidases which are characterized by catalytic mechanisms that require a divalent metal ion at the active site to hydrolyze the peptide bond^91^. In microorganisms, metallopeptidases are involved in regulating a diverse of biological processes ranging from nutrient absorption, extracellular matrix remodeling to microbial defense^92^. The MEROPS database (version 12.2) lists 76 families, among which 70 families are from 15 annotated clans and the rest have not been assigned into any clan. There are 77 putatively active and 6 inactive metallopeptidases in the *A. niger* genome. They belong to 8 clans and 34 subfamilies, and family M79 is not assigned to any clan, with the clans and families of 7 enzymes remain unknown (Table 7, Fig. 1a and Supplementary Table S2). It is interesting to note that in contrast to observations in other *Aspergillus* species^17^ where serine proteases are the largest group, metallopeptidases are the most abundant proteolytic enzymes in *A. niger*. According to the previously described method^93^, these metallopeptidases are also reclassified based on their active site architectures and overall fold similarities (Supplementary Table S5).

**Table 7:** Metallopeptidases encoded by *A. niger* strains CBS 513.88 and ATCC 1015.

Clan	Subfamily	Archetype	Provisional ID ^a^	Old locus tag ^b^	Conserved catalytic motif ^c^
MA	M1	Aminopeptidase N	Aminopeptidase Y	**An04g03930**	HEXXH + NEXXT/A + GXMEN + Y
			Leukotriene A-4 hydrolase	An05g00070	
			Aminopeptidase B	An09g06800	
	M3A	Thimet oligopeptidase	Thimet oligopeptidase	An07g00470	HEXXH + EXXS + H + Y/R + Y
				An15g02290	
			Oligopeptidase MepB	An07g01970	
			Mitochondrial intermediate peptidase	An11g05710	
	M4	Thermolysin	Thermolysin	An12g05900	HEXXH + NEXXS + Y + H
	M10A	Matrix metallopeptidase-1	Matrilysin	An12g02780	HQXXHXXGXXH + M
	M12A	Astacin	Flavastacin	An15g00830*	HEXXHXXGXXH + M
	M12B	Adamalysin	Adamalysin	An04g05530*	
				An15g03750*	
	M36	Fungalysin	Fungalysin	An01g02070*	HEYTH + YALESGGMGEGWSD + TYTSVNSLNAVHAIGTVWASILY
	M41	FtsH peptidase	*i*-AAA peptidase	An04g04970	HEXXH + E + ND + H_2_O
			*m*-AAA peptidase	An07g07000	
	M43B	Pappalysin-1	Pappalysin-1	An07g10410*	HEXXHXXGXXH + M
	M48A	Ste24 peptidase	Ste24 peptidase	An04g01950	HEXXH + EXXA + N + H
	M48C	Oma1 peptidase	/	An04g07380	
	M49	Dipeptidyl-peptidase III	/	An01g02980	
			Dipeptidyl-peptidase III	An04g00410	HEXXXH + EECRAE
	M57	prtB g.p	/	An14g01410	HEXXHXXGXXH + M
	M76	ATP23 peptidase	ATP23 peptidase	An05g00110	HEXXH
	M80	Wss1 peptidase	/	An01g05470	HEXXHXXXXXH
			/	An08g05390	
MC	M14A	Carboxypeptidase A1	/	An12g04170*	HXXE + R + NR + H + Y + E
ME	M16A	Pitrilysin	Ste23 peptidase	An07g06490	HXXEHX_76_EXXV/H + E
				An16g01860	
	M16B	Mitochondrial processing peptidase beta-subunit	Mitochondrial processing peptidase beta-unit	An01g12210	
			Mitochondrial processing peptidase alpha unit 2	An08g04080	
			/	An09g06650	
	M16C	Eupitrilysin	/	An04g01980	HXXEHX_76_EXXV/H + E
			Presequence protease (Prep)	An04g02320	
MG	M24A	Methionyl aminopeptidase 1	Methionyl aminopeptidase	An01g11340	
				An01g11360	
				An04g01330	
				An07g09120	
	M24B	Aminopeptidase P	Xaa-Pro dipeptidase	An01g13040	HXXGHXXGX_3-8_H
				An01g14920	
				An05g00050	
				An11g06960	
				An09g00700	
			Xaa-Pro aminopeptidase	An03g04230	
MH	M18	Aminopeptidase I	Aspartyl aminopeptidase	An02g11940	(S/G/A)HXDXV + P/GXXD + XEE + D/E + H
				An09g06250	
	M20A	Glutamate carboxypeptidase	Gly-Xaa carboxypeptidase	An02g13740*	
			/	An02g12680	
			/	An18g06210*	
	M20D	Carboxypeptidase Ss1	Met-Xaa dipeptidase	An01g11610	EE
				An02g00990	
				An08g07280	
				An11g07760	
				An11g08890	
				An12g02360	
				An15g01800	
	M20F	Carnosine dipeptidase II	Cytosol nonspecific dipeptidase	An04g10270	(S/G/A)HXDXV + P/GXXD + XEE + D/E + H
				An11g03000	
			/	An11g11180	
	M28A	Aminopeptidase S	Aminopeptidase Y	An03g01660*	
	M28B	Glutamate carboxypeptidase II	Glutamate carboxypeptidase II	An02g06300	
				An18g03980	
	M28E	Aminopeptidase Ap1	Leucyl aminopeptidase	An14g00620*	
				An17g00390*	
	M28F	YwaD peptidase	*i*-AAA peptidase	An04g02880*	
			*m*-AAA peptidase	An18g03780	
MJ	M19	Membrane dipeptidase	Membrane dipeptidase	An01g11740	
	M38	Isoaspartyl dipeptidase	/	An02g00090	HXH + K + H + H + D
				An11g05920	
				An14g02080	
				An14g03560	
				An15g04370	
MK	M22	O-sialoglycoprotein endopeptidase	Kinase-associated endopeptidase 1	An07g03020	HX(E/Q)XH + D + H
				An15g00900	
MP	M67A	RPN11 peptidase	RPN11 peptidase	An07g07860	EX_n_HXHX_10_D
			26S proteasome regulatory subunit RPN8	An07g10110	
	M67C	STAMBP isopeptidase	Endosome-associated ubiquitin isopeptidase	An02g12490	
M-	M79	CE1 peptidase	CAAX prenyl proteinase Rce1	An14g03420	EE

^a^ /, not provisionally identified;

^b^ Proteases that have been molecularly and/or biochemically characterized are in bold;

^c^ Putative catalytic residues, metal-binding residues and residues occupying the position of the Met-turn or Ser/Gly-turn beneath the metal sites are colored in pink, green and orange, respectively. Other residues involved in stabilization of the reaction intermediate, substrate binding, and/or catalysis are shown in black, except for X, which denotes any amino acid and is only used as a spacer within motifs;

* Secreted proteases that have been predicted by the six predictors, the unassigned extracellular metallopeptidase An06g00780 is not listed in this table.

### Clan MA

As observed in animals that clan MA is the largest among all metallopeptidases^91^, 24 of 83 putative metalloproteases in *A. niger* are from this clan, and they are further assigned into 13 families, M1, M3, M4, M10, M12, M36, M41, M43, M48, M49, M57, M76 and M80 (Table 7, Supplementary Table S2 and Supplementary Fig. S1f). In family M1, which is typified by aminopeptidase N from *H. sapiens*, one active enzyme (An04g03930) has been identified as lysyl aminopeptidase (also called aminopeptidase Y)^94^, which together with An09g06800 (a putative aminopeptidase B) are intracellular zinc aminopeptidases involved in the degradation of imported peptides^94^. An05g00070 is provisionally identified as leukotriene A-4 hydrolase which is a bifunctional zinc metalloenzyme with an anion dependent aminopeptidase activity and catalyzing the biosynthesis of leukotriene B4, a potent lipid chemoattractant engaged in inflammation, immune responses, and host defense against infection^95^. The *A. niger* genome also encodes 4 putatively active metallopeptidases in subfamily M3A. Among them, An07g00470, An07g01970 and An15g02290 are putative thimet oligopeptidases which are cytosolic zinc metallopeptidases probably participating in the intracellular digestion of small peptides^96^, whereas An11g05710 is a putative mitochondrial intermediate peptidase, a house keeping molecule of the mitochondrial protein import system required for maturation of nuclear proteins targeted to the mitochondria^97^. The *A. niger* genome putatively encodes a thermolysin (An12g05900) in family M4, which plays a significant role in microbial nutrition and acts as virulence factors^98^. In subfamily M10A, the single enzyme, An12g02780, is a putative matrilysin (also called matrixin or matrix metalloproteinase) which is widely involved in metabolism regulation via both selective peptide-bond hydrolysis and extensive protein degradation^99^. Family M12 in the *A. niger* genome contains 3 putatively active enzymes, including a flavastacin (An15g00830) and 2 adamalysins (An04g05530 and An15g03750) in subfamilies M12A and M12B, respectively (Table 7). Flavastacin is an O-glycosylated zinc metallopeptidase that cleaves peptides from the N-terminal side of aspartic acid^100^, while adamalysins are implicated in the processing of extracellular and cell surface matrix proteins^101^. In family M36, the *A. niger* genome encodes a putative fungalysin (An01g02070) which is the secreted fungal peptidase capable of degrading the extracellular matrix proteins collagen and elastin, and acting as virulence factors in diseases caused by fungi^11^. An04g01950 from subfamily M48A is provisionally identified as ste24 peptidase, a zinc metalloprotease catalyzing two proteolytic steps in the maturation of the yeast mating pheromone α-factor^102^. In family M49, An04g00410 is putatively identified as dipeptidyl-peptidase III which participates in intracellular peptide metabolism^103^, whereas An01g02980 doesn’t contain the unique hexapeptide linear motif HEXXXH (X represents any amino acid) and may thus unlikely to carry out proteolysis. The single enzyme sequence in family M76 (An05g00110) encodes the mitochondrial inner membrane protease ATP23 with dual function in the processing and assembly of subunit 6 of mitochondrial ATPase^104^. The rest 6 enzymes from families/subfamilies M41, M43B, M48C, M57 and M80 have not been provisionally identified (Table 7). Most clan MA metallopeptidases are endopeptidases, except aminopeptidases from family M1 which release N-terminal lysine and/or arginine from oligopeptides^94^ and dipeptidyl aminopeptidases of family M49 that cleave an N-terminal dipeptide from an oligopeptide comprising four or more residues, with broad specificity.

As shown in Supplementary Table S5, clan MA peptidases are all zinc metallopeptidases that have been grouped into the zincin tribe based on their active sites architecture and overall fold similarities. Fourteen enzymes from families/subfamilies M1, M3A, M4, M36, M41, M48A, M48C and M76 share the canonical zinc binding motif HEXXH (Table 7, Supplementary Fig. S2f.)^93,105^, and An04g00410 in family M49 contains the exceptional zinc ion ligand motif HEXXXH^103^. Most of these enzymes belong to the gluzincins clan which uses a glutamate as the third metal-binding residue^93^, except those from family M41 which are FtsH-like AAA metallopeptidases and An05g00110 from family M76 that can not be assigned into any clan (Supplementary Table S4). Six metallopeptidases from subfamilies M10A, M12A, M12B, M43B and M57 bear an extended motif H(Q/E)XXHXXGXXH/D, while An01g05470 and An08g05390 from family M80 possess the extended motif HEXXHXX(H/F)XXH. According to their active site architectures and overall fold similarities, these eight enzymes are reassigned into clan metzincins (Supplementary Table S5). The histidines in these conserved motifs are involved in zinc-binding, while the glutamates or gultamines act as general bases/acids during catalysis^93,99^.

### Clan MC

This clan contains 3 families: M14, M86 and M99^5^. Among them, family M14 is composed of carboxypeptidases, which is further divided into 4 subfamilies: M14A (digestive carboxypeptidases), M14B (regulatory carboxypeptidases), M14C (bacterial peptidoglyan hydrolyzing enzymes) and M14D (cytosolic carboxypeptidases)^5^. The *A. niger* genome encodes only 1 putatively active metallocarboxypeptidase (An12g04170) from subfamily M14A which has not been provisionally identified (Table 7, Supplementary Fig. S1f.). Based on the active site architecture and overall fold similarity, An12g04170 is reassigned into funnelins subfamily A of αβα-exopeptidases which contains the conserved motif HXXE (Supplementary Table S5, Supplementary Fig. S2f.).

### Clan ME

MEROPS database (version 12.2) lists 2 families in clan ME: family M16, subdivided into pitrilysin-like enzymes in subfamily M16A, the mitochondrial processing peptidase in subfamily M16B, and eupitrilysin-like enzymes in subfamily M16C as well as lastly family M44 typified by pox virus metallopeptidase from *Vaccinia virus*^5^. The *A. niger* genome encodes 7 metalloendopeptidases from family M16 in this clan, among which An07g06490 and An16g01860 in subfamily M16A are putative Ste23 peptidases (Table 7), and their only known function is α-factor processing in *Saccharomyces cerevisiae*^106^. Of the 3 enzymes in subfamily M16B, An01g12210 and An08g04080 are provisionally identified as mitochondrial processing peptidase beta unit and alpha unit 2, respectively, which are involved in the processing of signal peptides of mitochondrial protein imports^107^. In subfamily M16C, An04g02320 is a putative metallopeptidase 1 (also known as presequence protease, Prep) which cleaves off presequence of nuclear encoded mitochondrial precursor proteins^108^, whereas An04g01980 has not been provisionally identified. Based on their active site architectures and overall fold similarities, these family M16 metallopeptidases have been reassigned into tribe inverzincins (Supplementary Table S4), most of which contain the characteristic inverted HXXEHX_76_EXXV/H zinc-binding motif (X represents any amino acid) and glycine-rich region (Table 7, Supplementary Fig. S2f)^107^. The EXXV motif encompasses the third glutamate metal-binding residue and a valine as the Ser/Ala-turn residue, as well as a mixed β-sheet of at least three strands equivalent to zincins^93^. However, An08g04080 and An09g06650 from subfamily M16B lack this motif and the R/Y pairs found in the C-terminal half of family M16 enzymes^109^, and are thus unlikely to possess proteolytic activities.

### Clan MG

This clan contains one single family, M24, which is subdivided into subfamilies M24A and M24B typified by methionyl aminopeptidase 1 and aminopeptidase P, respectively^5^. The *A. niger* genome encodes 10 putatively active enzymes in family M24 (Table 7), and they have been provisionally identified as methionyl aminopeptidaes (EC 3.4.11.18; An01g11340, An01g11360, An04g01330 and An07g09120), Xaa-proline dipeptidases (also called prolidase, EC 3.4.13.9; An01g13040, An01g14920, An05g00050, An11g06960 and An09g00700) and Xaa-proline aminopeptidase (EC 3.4.11.9; An03g04230). In microorganisms, methionyl aminopeptidases remove the N-terminal initiator methionine from nascent polypeptides in a non-processive manner^110^, and Xaa-proline dipeptidases cleave dipeptides with proline or hydroxyproline at the N-terminal position and are involved in collagen turnover^111–113^, while Xaa-Pro aminopeptidase releases any N-terminal amino acid, including proline, that is linked to proline, even from a dipeptide or tripeptide^113^. As compared with Xaa-proline dipeptidase, Xaa-Pro aminopeptidase plays a more important role in nitrogen nutrition^114^.

Subfamily M24B enzymes contain the conserved HXXGHXXGX_3-8_H motif, where the N- and C-terminal histidines are the nucleophiles and the middle histidine is involved in metal ion binding, while methionyl aminopeptidases from subfamily M24A have bidentate ligands which bind metal ions and a metal-bridging water or hydroxide ion that acts as the nucleophile during catalysis (Supplementary Table S2 and Supplementary Fig. S2f.)^110^. However, An09g00700 from subfamily M24B doesn’t contain the conserved motif and may be proteolytically inactive.

### Clan MH

This clan is composed of 4 families, M18, M20, M28 and M42^5^. As the second largest clan in *A. niger*, it contains 2, 13 and 7 putatively active enzymes from families M18, M20 and M28, respectively (Table 7 and Supplementary Table S2). Clan MH contains aminopeptidases, dipeptidases and carboxypeptidases. Among these enzymes, members of families/subfamilies M18, M28A and M28E are aminopeptidases. In family M18, An02g11940 and An09g06250 have been provisionally identified as aspartyl aminopeptidases which have implicated roles in peptide and protein metabolisms, and the renin-angiotensin system in blood pressure regulation^115^. Besides An04g03930 in family M1 from clan MA, the single enzyme (An03g01660) of subfamily M28A is also provisionally identified as aminopeptidase Y, while An14g00620 and An17g00390 from subfamily M28E are putative leucyl aminopeptidases, the housekeeping enzymes necessary for protein turnover^116^. Ten enzymes from subfamilies M20D and M20F are dipeptidases, and seven of them have been provisionally identified as Met-Xaa dipeptidases (Table 7 and Supplementary Table S2), which catalyze the hydrolysis of Met-Xaa dipeptides^117^. Of the remaining 3 enzymes from family M20F, An04g10270 and An11g11180 have been provisionally identified as cytosolic nonspecific dipeptidases like carnosine dipeptidase II which has L-carnosine hydrolyzing activity^118,119^. Five enzymes from subfamilies M20A and M28B are carboxypeptidases. Among them, one enzyme (An02g13740) in subfamily M20A is a putative Gly-Xaa carboxypeptidase which releases C-terminal amino acids from a peptide with glycine as the penultimate amino acid, whereas An14g00620 and An17g00390 in subfamily M28B have been provisionally identified as glutamate carboxypeptidase II, a membrane-bound extracellular carboxypeptidase hydrolyzing the neuropeptide N-acetylaspartylglutamate^120^. An04g02880 and An18g03780 from subfamily M28F are putative *i*-AAA and *m*-AAA peptidases, respectively, which coordinately regulate OMA1 (a zinc metallopeptidase of the inner mitochondrial membrane) processing and turnover^121^.

Based on the active site architectures and overall fold similarities, enzymes from subfamilies M18, M20A, M20F, M28A, M28B, M28E and M28F are reassigned into the aminoacylase-1 family of the αβα-exopeptidases tribe, with the catalytic amino acid residues contained in the conserved motif (S/G/A)HXDXV + P/GXXD + XEE + D/E + H (X denotes any amino acid), whereas members of subfamily M20D contain the conserved EE motif and belong to EEM2-MPs family of the αβα-exopeptidases tribe (Supplementary Fig. S2f and Supplementary Table S5).

### Clan MJ

This clan contains 2 families, M19, which is typified by membrane dipeptidase, and M38 represented by isoaspartyl dipeptidase which participates in the processing of isoAsp dipeptides^5,122^. The *A. niger* genome encodes 1 and 5 putatively active enzymes from families M19 and M38, respectively (Table 7, Supplementary Table S2 and Supplementary Fig. S1f). The single enzyme from family M19, An01g11740, is a putative membrane dipeptidase involved in the metabolism of glutathione and its conjugates, especially leukotriene D_4_^123^, whereas the 5 members of family M38 have not been provisionally identified.

An01g11740 may be a novel Zincin, as it contains none of the major zinc peptidase motifs such as HEXXH or HXXEH^123^, but has a region (DHIMYIGNLIGFDH; residues 361–374; Supplementary Fig. S2f) sharing close similarities with the crystallographically identified zinc-binding motif (DHTH) in D-alanyl-D-alanine-cleaving carboxypeptidase of *Streptomyces albus* G^124^ and the region (DHLDH) in the membrane dipeptidase from pig kidney cortex^123^. As aligned with the amino acid sequence of the membrane dipeptidase from pig kidney cortex, two amino acid residues of An01g11740 (H77 and H270) are predicted to be involved in the catalysis, while H220 is implicated in the binding of substrate or inhibitor^123^. Family M38 enzymes contain the conserved motif HXH + K + H + H + D, where aspartate is the nucleophile, while histidines and lysine are metal binding residues (Table 7 and Supplementary Fig. S2f).

### Clan MK

This clan comprises of only one family, M22, which is typified by O-sialoglycoprotein endopeptidase, an enzyme specifically cleaving the protein part of O-glycosylated proteins on threonine or serine residues^125^. The *A. niger* genome encodes two putatively active kinase-associated endopeptidase 1 (An07g03020 and An15g00900; Table 7, Supplementary Table S2), which has been shown to be essential for the cell growth of *S. cerevisiae*^125^. These two enzymes contain the conserved motif HX(E/A/Q)XH (X represents any amino acid, Supplementary Fig. S2f), where the two histidine residues are the catalytic dyad^126^.

### Clan MP

This clan contains proteases from a single family, M67, which is part of the ubiquitin cellular regulatory system^127^. In the *A. niger* genome, An07g07860 and An07g10110 from subfamily M67A are provisionally identified as 26S proteasome regulatory subunits PRN11 and RPN8, respectively, while An02g12490 in subfamily M67C is a putative endosome-associated ubiquitin isopeptidase (Table 7), which is the member of the JAB1/MPN/MOV34 (JAMM) family of DUBs catalyzing the hydrolysis of isopeptide (or peptide) bonds between ubiquitin and target proteins or within polymeric chains of ubiquitin^128^. These enzymes contain the highly conserved JAMM motif EX_n_HXHX_10_D (X represents any amino acid), where the two histidine residues and the aspartate residue bind a zinc ion, and the glutamate acts as a general base/acid during catalysis^127^.

### Other metallopeptidases

The version 12.2 MEROPS database lists 6 families of metallopeptidases (M73, M77, M79, M82, M87 and M96) which have not been assigned into any clan^5^. The *A. niger* genome encodes one active enzyme (An14g03420) from family M79 which is provisionally identified as CAAX prenyl proteinase Rce1, an enzyme capable of processing all farnesylated and geranylgeranylated CAAX proteins^129^. This enzyme contains the conserved motif EE and belongs to clan EEM2-MPs in αβα-exopeptidases tribe (Supplementary Table S4). There are four metallopeptidases in the *A. niger* genome that can not be assigned into any clan and family. Among them, An06g01580 contains the classical zinc binding motif HEXXH, thus belonging to the gluzincins clan of zincin tribe, while An02g06910 possesses the extended motif HEXXHXXGXXH and may be an ascomycolysin within the metzincins clan of zincin tribe (Supplementary Fig. S2f and Supplementary Table S5)^130^. An06g00780 and An07g03400 may belong to aminoacylase-1 family of αβα-exopeptidases tribe, as they contain the conserved motif (S/G/A)HXDXV + P/GXXD + XEE + D/E + H (Supplementary Fig. S2f and Supplementary Table S5). However, An18g05100 may be not a metallopeptidase, as it shares high similarity (43.74%) with cytosine deaminase (GenBank accession No: EMT69769.1) from *Fusarium oxysporum* f. sp. *cubense* race 4 and contains the equivalent active sites and metal-binding residues (H65, H67, H227, H265, N337)^131^. Further studies are thus needed to identify the exact function of An18g05100 in the future.

## Discussion

Our understanding of peptidase diversity and complexity has expanded rapidly in the post-genomic era, and it has been found that degradome composition varies greatly between kingdoms of life with surprisingly little apparent variation in subkingdoms and their phyla. However, studies on the comprehensive identification and comparison of the degradomes mainly focus on mammals for therapeutical benefit^10,12,13,132^. The degradomes of many industrially important microorganisms have not been investigated. In the present study, we have performed a more precise and comprehensive genomic analysis of the complete set of proteases in *A. niger*, and the 232 putative proteases presented here cover ~ 1.64% of the 14,165 putative protein sequences in the *A. niger* genome (Table 1 and Supplementary Table S2). Comparatively, this protease content is larger than that in *Ixodes scapularis* (~ 1.14%)^8^, similar to that in yeast (~ 1.7%), but smaller than what has been observed in other organisms, such as ~ 1.74% in *Meloidogyne incognita*^7^, ~ 1.8% in humans^133^, ~ 3.4% in *Drosophila*, ~ 2% in *Culex quinquefasciatus*, ~ 2.4% in *Anopheles gambiae*, and ~ 3.7% in *Aedes aegypti*^8^. It should be emphasized here that basic delineation and verification of these newly characterized genomes are still running, and the degradome sizes are thus subject to change. The 91 non-protease homologues in *A. niger* represent ~ 0.64% of its genome, which is comparable to the non-peptidase contents of other organisms, e.g., ~ 0.65, 0.64, 1.06 and 1.7% in *I. scapularis*, *C. quinquefasciatus*, *A. aegypti*, and *A. gambiae*^8^, respectively. Given that we do not have gene expression data in this study, whether or not non-protease homologues are expressed is still unknown at this point.

The full repertoire of peptidases in the *A. niger* genome has been further assigned into 71 families/subfamilies and 26 clans of the six known catalytic classes by the MEROPS classification system (Fig. 1 and Supplementary Table S2), i.e., 17 aspartic, 5 glutamic, 14 threonine, 41 cysteine, 72 serine and 83 metallo-proteases. Different classes of peptidases associate with vital biological pathways. Peptidases within a clan tend to have similar functions and properties, since they have similar structure although they can also differ greatly, even to the extent of being of different catalytic types^40^. As stated above, aspartic peptidases may perform important functions related to nutrition and pathogenesis in *A. niger*^1,23,24^. Glutamic peptidases are involved in a variety of processes like digestion^31,35,36^. Threonine peptidases function as the central conduit for protein turnover^37^. Cysteine peptidases are mainly involved in the housekeeping autophagy^44^, ubiquitin^45,46^, and SUMOylation^57^ cellular homeostasis regulatory mechanisms, which regulate protein turnover. Serine peptidases are implicated in protein digestion and processing, cell signaling, protein quality control, intracellular protein turnover, and flavour development^60,61,73,82,86,87^. Metallopeptidases regulate a diverse of biological processes ranging from nutrient absorption, protein turnover, extracellular matrix remodeling to microbial defense^92^. However, since very few data are currently available concerning the biochemical characterization of these proteases in *A. niger* (Supplementary Table S4), we mainly provide guiding descriptions of their probable functions where available. Further molecular and biochemical studies are therefore highly warranted to elucidate the exact biological functions of these proteases.

The active sites and active site architectures of all the peptidases in the *A. niger* genome were also characterized (Tables 2–7, Supplementary Table S2 and Fig. S2). Within each protease class, most, but not all, clans consist of one active site arrangement^93^. However, some members of the same clan can use different active site architectures, indicating that the tertiary structure is not always related to the same active site configuration^60^. Threonine peptidases use the Thr-only catalytic mechanism which is involved in autoproteolysis to generate mature proteasome (Table 4, Fig. S2)^134^, while aspartic and glutamic peptidases utilize the Asp/Asp and Gln/Glu catalytic dyads (Tables 2 and 3), respectively. As shown in Tables 5 and 6, the active site configurations of cysteine and serine peptidases are more complex, which usually consist of different catalytic dyads and triads. Besides active site residues, metallopeptidases also contain metal-binding residues which are mostly histidines, aspartates and glutamates (Table 7). In addition, the loop structures which are termed “Ser/Gly-turn” and “Met-turn” in gluzincins and metzincins (Table 7 and Supplementary Fig. S2f), respectively, provide a basement for the metal-binding site. It has also been found in serine peptidases that different active site arrangements used by these proteases may allow for activity in a different cellular environment^60^. The optimum pH of the peptidase is affected by the pKa value of the general base residue that is employed in catalysis. For instance, the proteases with Ser/Glu/Asp active sites (such as sedolisin proteases) typically carry out catalysis with a pH optimum that is lower than Ser/His/Asp proteases^60^. In contrast, the pH optimum is lower for serine proteases with Ser/Lys active sites than those with Ser/His/Asp active sites. Moreover, variations in the active site configurations of proteases may also influence what cellular inhibitors they are susceptible to. For example, many Ser/Lys proteases are not inhibited by the classical serine peptidase inhibitors such as diisopropyl fluorophosphates (DFP) or phenylmethanesulfonyl fluoride (PMSF)^60,135^. Future studies will provide deeper insights into the reasons why alternative active site geometries have arisen during evolution.

## Conclusion

The availability of the genome sequence of *A. niger* strains CBS 513.88 and ATCC 1015 has allowed the analysis of the full repertoire of proteases from this industrially important fungus. The *A. niger* degradome consists of at least 232 putative proteases, which represent ~ 1.64% of the 14,165 putative protein sequences in *A. niger*. To clarify peptidase diversity, the complete set of proteases within the *A. niger* genome was precisely assigned into the 71 families/subfamilies and 26 clans of the six known catalytic classes by the MEROPS classification system. The active sites, metal-binding residues and active site architectures of these peptidases were then characterized. Guiding descriptions of their probable functions were also provided. Our results provide a landscape of genome-wide peptidase diversity in *A. niger* that enables a framework for decoding function, architecture and evolution of proteolysis in vivo, which will also lay a solid foundation for the further molecular and biochemical characterization of these proteases.

## Methods

### Genome mining and extracellular proteases prediction

The complete set of predicted gene models encoding proteases in the genomes of *A. niger* strains CBS 513.88 and ATCC 1015 were retrieved from AspGD (http://www.aspergillusgenome.org/, version s01-m07-r13, February 2020), JGI (https://genome.jgi.doe.gov/Aspni1, version 4.0), and version 12.2 MEROPS database^20,21^. Besides these proteases, other homologues were added to the clusters by homolog searches against the non redundant database at NCBI (https://www.ncbi.nlm.nih.gov/) using each single *A. niger* protease as the query sequence in a BLASTP analysis^136^, with default settings. The functional domains of these proteases were identified by Conserved Domain Database on NCBI (CDD, https://www.ncbi.nlm.nih.gov/cdd) and hmmscan of the HMMER v3.3 package (https://www.ebi.ac.uk/Tools/hmmer/search/hmmscan)^137^ with default parameters, using the complete Pfam-A and Pfam-B models (data retrieved from Pfam 33.1 database)^138^. The predictions obtained were pooled, and proteins containing no known protease-related Pfam domain(s) were removed when no additional literature support could be found.

Theoretical subcellular localization of the proteases identified was predicted by SignalP 4.1 (http://www.cbs.dtu.dk/services/SignalP/), WoLF-PSORT (https://wolfpsort.hgc.jp/), PrediSi (http://www.predisi.de/), CELLO (http://cello.life.nctu.edu.tw/), Phobius (https://www.ebi.ac.uk/Tools/pfa/phobius/) and TargetP (http://www.cbs.dtu.dk/services/TargetP). Default settings of each predictor were used, with the species parameters as “Fungi” or “Eukaryotic”. Majority votes were applied to combine the results of each prediction.

### Protease family reassignment

The proteases were assigned into the clans, families and subfamilies according to the MEROPS database (version 12.2, https://www.ebi.ac.uk/merops/)5 and manual literature searches. These proteases were then reclassified based on their EC numbers and the literatures published by Andersen et al*.* and Guillemette et al*.*^20,21^. EC numbers were assigned based on enzyme database BRENDA (http://www.brenda.uni-koeln.de/), hmmsearch of HMMER v3.3 package (https://www.ebi.ac.uk/Tools/hmmer/search/hmmsearch), and the described function of the selected similar protein and the degree of similarity as previously described^18^.

### Characterization of the active sites and metal-binding residues of these proteases

The active sites and metal ligands of the proteases were predicted by MEROPS batch BLAST^139^. Additionally, sequence alignment analysis based on the homologous enzyme sequences with known catalytic residues was also employed to confirm and recharacterize the active sites, metal-binding residues, and conserved motifs of the proteases. The reported peptidases used for active site characterization and their homologues identified in *A. niger* strains CBS 513.88 and ATCC 1015 are listed in Supplementary Table S1. Putatively active or hypothetically inactive protease homologs were identified by the presence or absence of consensus active site amino acid residues, respectively, whereas non-peptidase homologues are those lacking any of the active sites and metal binding residues known for the protease family^139^.

### Identification of proteases that have been characterized

To identify proteases that have been characterized by molecular, biochemical and/or omics techniques, a computer-based comprehensive literature search was carried out by full text search in Pubmed, Google, ScienceDirect, Springer, Web of Science, etc.

### Multiple sequence alignment and phylogenetic analysis

Multiple amino acid sequence alignments were performed with the Clustal X2 and BioEdit version 7.0.9 software packages, using standard parameters. After visually examined and edited, the alignments were subjected to phylogenetic analysis using the Maximum Parsimony approach as implemented in program MEGA 6.0^140^. Bootstrap resampling with 1000 pseudoreplicates was conducted to assess support for each individual branch.

## Supplementary information

Supplementary Information 1.

Supplementary Table S1.

Supplementary Table S2.

Supplementary Table S3.

Supplementary Table S4.

Supplementary Table S5.

Supplementary Figure S1.

Supplementary Figure S2.
