# Supplementary material for: Bioinformatic mapping of a more precise Aspergillus niger degradome
Source: Sci Rep. 2021 Jan 12;11:693. doi: 10.1038/s41598-020-80028-3 (PMC7804941; doi:10.1038/s41598-020-80028-3)
Supplement: Supplementary file 1 — Supplementary Information 1. [file 41598_2020_80028_MOESM1_ESM.doc]

**Supplementary materials**

**Scientific Reports**

**Bioinformatic mapping of a more precise *Aspergillus niger* degradome**

**Zixing Dong****^1^†^*^ • Shuangshuang Yang^2^† • Byong Hoon Lee^3^**

^1^ Henan Provincial Engineering Laboratory of Insect Bio-reactor and Henan Key Laboratory of Ecological Security for Water Region of Mid-line of South-to-North, Nanyang Normal University, Nanyang 473061, China

^2^ College of Physical Education, Nanyang Normal University, Nanyang 473061, China

^3^ Department of Microbiology/Immunology, McGill University, Montreal, QC, Canada

† These authors contributed equally to this work.

*** Corresponding author**

Zixing Dong

E-mail: [star1987.com@163.com](mailto:star1987.com@163.com); dzx@nynu.edu.cn

Address: Henan Provincial Engineering Laboratory of Insect Bio-reactor, Nanyang Normal University, 1638 Wolong Road, Nanyang, Henan 473061, People’s Republic of China

Telephone: +86-377-63525087

Fax: +86-377-63525087
