# Supplementary material for: Bioinformatic mapping of a more precise Aspergillus niger degradome
Source: Sci Rep. 2021 Jan 12;11:693. doi: 10.1038/s41598-020-80028-3 (PMC7804941; doi:10.1038/s41598-020-80028-3)
Supplement: Supplementary file 2 — Supplementary Table S1. [file 41598_2020_80028_MOESM2_ESM.doc]

**Supplementary Table S1.** Peptidases used for the identification of the active sites, metal-binding residues, conserved motifs and residues/molecules occupying the position of the Met-turn or Ser/Gly-turn beneath the metal sites of the homologues in *Aspergillus niger* CBS 513.88 and ATCC 1015

| **Peptidases reported in the literature (Organism)** | **Family/Subfamily** | **GenBank accession No.** | **References** | **Homologues identified in this study** |
| --- | --- | --- | --- | --- |
| BcAP1 (*Botrytis cinerea*) | A1A | AF121229.1 | [^1^](#_ENREF_1) | 53364  An01g00370  An02g07210  An04g01440  An07g00950  An11g00310  An11g09170  An12g03300  An13g02130  An14g04710  An15g06280  An15g07770  An18g01320 |
| Intramembrane protease 2 (*Caenorhabditis elegans*) | A22B | NP_502079.1 | [^2^](#_ENREF_2) | An15g02400 |
| AGP (*A. niger* var. *macrosporus*) | G1 | 1Y43_B | [^3^](#_ENREF_3) | 126639  An01g00530  An07g00320  An14g03250  An15g07700 |
| Ta_beta (*Thermoplasma acidophilum*) | T1 | P28061.1 | [^4^](#_ENREF_4) | An02g03400  An02g07040  An02g10790  An07g02010  An11g01760  An11g04620  An11g06720  An13g01210  An18g06680  An18g06800 |
| LAP3 *(Saccharomyces cerevisiae* S288C) | C1B | Q01532.3 | [^5^](#_ENREF_5) | An01g01720 |
| PalBH (*Homo sapiens*) | C2A | BAA78730.1 | [^6^](#_ENREF_6) | An01g04680  An11g02950 |
| Uch2p (*Schizosaccharomyces pombe*) | C12 | CAB52608.1 | [^7^](#_ENREF_7) | An01g11160  An02g13920  An08g11630  An11g11130  An12g01820 |
| GPI8 (*Drosophila melanogaster*) | C13 | Q8T4E1.1 | [^8^](#_ENREF_8) | An01g13530 |
| Yca1 (*S. cerevisiae* S288C) | C14B | NP_014840.4 | [^9^](#_ENREF_9) | An09g04470  An18g05760 |
| Pyrrolidone carboxyl peptidase (*Thermococcus litoralis*) | C15 | 1A2Z_A | [^10^](#_ENREF_10) | An11g01970 |
| DOA4 (*S. cerevisiae*) | C19 | AAC48915.1 | [^11^](#_ENREF_11) | An11g04380 |
| SUMO-1-specific protease (*H. sapiens*) | C48 | AAF04852.1 | [^12^](#_ENREF_12) | An09g05400  An13g01190 |
| Ulp 1 (*S. cerevisiae* S288C) | C48 | Q02724.1 | [^13^](#_ENREF_13) | An14g05500 |
| BimB (*A. nidulans* FGSC A4) | C50 | P33144.2 | [^14^](#_ENREF_14) | An07g03090 |
| HsAtg4B (*H. sapiens*) | C54 | Q9Y4P1.2 | [^15^](#_ENREF_15) | An11g11320 |
| Protease I (*Pyrococcus furiosus* DSM 3638) | C56 | AAB04694.1 | [^16^](#_ENREF_16) | An16g00930 |
| OTUB1 (*H. sapiens*) | C65 | NP_060140.2 | [^17^](#_ENREF_17) | 39420 |
| OTU domain-containing protein 6B (*Paracoccidioides lutzii* Pb01) | C85A | XP_002791043.1 | [^18^](#_ENREF_18) | An01g09320 |
| OTU1 (*S. cerevisiae* S288C) | C85B | P43558.1 | [^19^](#_ENREF_19) | An11g11090 |
| DEG1 (*Arabidopsis thaliana*) | S1D | O22609 | [^20^](#_ENREF_20) | An08g08670 |
| PepC, PepD and KexB (*A. niger*) | S8 | AAA32702.1, AAA32703.1, CAB64692.1 | [^21-23^](#_ENREF_21) | An01g08530  An02g02850  An07g03880  An09g03780  An14g01380  An14g01530  An16g06260  An18g02630  An18g04970 |
| DPP IV (*A. oryzae* RIB40) | S9 | Q2UH35.1 |  | An01g01210  An02g11420  An04g02850  An09g02830  An12g04700  An16g08150 |
| Carboxypeptidase O (*A. oryzae* RIB40) | S10 | XP_001824682.3 | [^26^](#_ENREF_26) | An02g04690  An03g05200  An05g01870  An05g02170  An06g00310  An07g08030  An08g00430  An08g08750  An11g06350  An14g02150  An16g09010  An17g00760 |
| Bs-Dap (*Brucella* sp.) | S12 | ENT06814.1 | [^27^](#_ENREF_27) | An09g00950  An16g06750 |
| ClpP (*Plasmodium falciparum* 3D7) | S14 | XP_001351149.1 | [^28^](#_ENREF_28) | An02g11960 |
| X-PDAP (*Lactococcus lactis* subsp. *lactis* NCDO 763) | S15 | P22346.1 | [^29-32^](#_ENREF_29) | An16g06560 |
| Endopeptidase La (*Actinobacillus pleuropneumoniae*) | S16 | WP_005600483.1 | [^33^](#_ENREF_33) | An02g03760  An18g02980 |
| Endopeptidase catalytic subunit IMP1 (*S. cerevisiae* S288C) | S26 | NP_013870.1 | [^34^](#_ENREF_34) | An01g00560  An09g02730  An14g06320 |
| ProtA/Epr (*A. niger*) | S28 | AX458699.1 | [^35-39^](#_ENREF_35) | An08g04490  An12g05960  An14g01120 |
| PapA (*A. niger*) | S33 | CAC40647.3 |  | An11g04730 |
| Hip1 (*Mycobacterium tuberculosis*) | S33 | 5UGQ_A | [^42^](#_ENREF_42) | An03g02530  An12g08560  An13g02620  An13g02790 |
| PepI (*L. delbrueckiiv* DSM 7290) | S33 | CAA81556.1 | [^43^](#_ENREF_43) | An16g06070 |
| Aorsin (*A. oryzae*)  Sedolisin (*A. fumigatus*) | S53 | AB084899  AJ585109 |  | An01g01750  An03g01010  An06g00190  An08g04640  An11g01110  An14g02470  An16g02250 |
| Rhomboid protease GlpG (*E. coli*) | S54 | P09391.5 |  | An08g00670  An08g10730  An15g06920 |
| YscII (*S. cerevisiae*)  Lysine aminopeptidase (*A. niger*) | M1 | CAA45403.1  CAC38353.1 |  | An04g03930  An05g00070  An09g06800 |
| MepB (*A. fumigatus*) | M3 | AAB66656.1 | [^50^](#_ENREF_50) | An07g00470  An07g01970  An11g05710  An15g02290 |
| Gentlyase (*Paenibacillus polymyxa*) | M4 | 4GER_A | [^51^](#_ENREF_51) | An12g05900 |
| Matrilysin (*H. sapiens*) | M10A | AAC37543.1 | [^52^](#_ENREF_52) | An12g02780 |
| Aspartyl endopeptidase (*Elizabethkingia meningoseptica*) | M12A | AAC41455.1 | [^53^](#_ENREF_53) | An15g00830 |
| VAP2 ([*Crotalus atrox*](https://www.ncbi.nlm.nih.gov/Taxonomy/Browser/wwwtax.cgi?id=8730)) | M12B | 2DW0_A | [^54^](#_ENREF_54) | An04g05530  An15g03750 |
| Metallocarboxypeptidase (*Pseudomonas aeruginosa*) | M14A | 4A37_A | [^55^](#_ENREF_55) | An12g04170 |
| Ste23p (*S. cerevisiae* S288C) | M16A | Q06010.2 | [^56^](#_ENREF_56) | An07g06490  An16g01860 |
| Mitochondrial processing peptide subunit beta (*Trypanosoma brucei*) | M16B | XP_803756.1 | [^57^](#_ENREF_57) | An01g12210  An08g04080  An09g06650 |
| Zinc metallopeptidase (*A. thaliana*) | M16C | AAG13049.1 | [^58^](#_ENREF_58) | An04g01980  An04g02320 |
| Aspartyl aminopeptidase (*H. sapiens*) | M18 | Q9ULA0.1 | [^59^](#_ENREF_59) | An02g11940  An09g06250 |
| Membrane dipeptidase (*H. sapiens*) | M19 | 1ITQ_A | [^60^](#_ENREF_60) | An01g11740 |
| Carnosine dipeptidase I (*Anguilla japonica*) | M20A | BAF48785.1 | [^61^](#_ENREF_61) | An02g12680  An02g13740  An18g06210 |
| SACOL0085 (*Staphylococcus aureus* COL)  HmrA (*S. aureus*) | M20D | 4EWT_A  Q99Q45 |  | An01g11610  An02g00990  An08g07280  An11g07760  An11g08890  An12g02360  An15g01800 |
| Carnosine dipeptidase II (*A. Japonica*; *Mus musculus*) | M20F | BAL62981.1  NP_075638.2 |  | An04g10270  An11g03000  An11g11180 |
| Sialoglycoprotease GCP1 (*A. thaliana*) | M22 | AAK00530.1 |  | An07g03020  An15g00900 |
| Methionine aminopeptidase (*Babesia bovis*) | M24A | XP_001610600.1 | [^68^](#_ENREF_68) | An01g11340  An01g11360  An01g13040  An01g14920  An03g04230  An04g01330  An05g00050  An07g09120  An11g06960 |
| Prolidase (*L. lactis*) | M24B | 4ZNG_A | [^69^](#_ENREF_69) | An09g00700 |
| [A](https://blast.ncbi.nlm.nih.gov/Blast.cgi#alnHdr_54792592)minopeptidase (*Streptomyces griseus*) | M28A | 1CP7_A |  | An02g06300  An03g01660  An14g00620  An17g00390  An18g03980 |
| Membrane glutamate carboxypeptidase (*H. sapiens*) | M28B | Q04609.1 |  | An02g06300  An18g03980 |
| Leucine aminopeptidase 2 (*A. fumigatus*) | M28E | AAR96058.1 | [^74^](#_ENREF_74) | An14g00620  An17g00390 |
| Murine QC (*Mus musculus*) | M28F | BAB30831.1 | [^75^](#_ENREF_75) | An04g02880  An18g03780 |
| Extracellular metalloproteinase (*A. fumigatus*) | M36 | 4K90_A | [^76^](#_ENREF_76) | An01g02070 |
| Xaa-Pro dipeptidase (*Nonlabens marinus* S1-08) | M38 | BAO55078.1 | [^77^](#_ENREF_77) | An02g00090  An11g05920  An14g02080  An14g03560  An15g04370 |
| MAP-1 (*Neurospora crassa*) | M41 | AF323912.1 | [^78^](#_ENREF_78) | An04g04970  An07g07000 |
| Metalloprotease (*Pleurotus ostreatus*) | M43B | AAU94648.1 | [^79^](#_ENREF_79) | An07g10410 |
| Ste24p (*S. mikatae*) | M48 | 4IL3_A | [^80^](#_ENREF_80) | An04g01950  An04g07380 |
| DPPIII (*S. cerevisiae*) | M49 | 3CSK_A |  | An01g02980  An04g00410 |
| prtB g.p. (*Grillisia forsetii*) | M57 | WP_026935415.1 | [^83^](#_ENREF_83) | An14g01410 |
| RPN11 peptidase (*S. cerevisiae*) | M67A | P43588.1 | [^84^](#_ENREF_84) | An07g07860  An07g10110 |
| Deubiquitinase AMSH (*H. sapiens*) | M67C | O95630.1 | [^85^](#_ENREF_85) | An02g12490 |
| Atp23 peptidase (*H. sapiens*) | M76 | NP_150592.1 | [^86^](#_ENREF_86) | An05g00110 |
| RCE1 peptidase (*S. cerevisiae*) | M79 | Q03530 | [^87^](#_ENREF_87) | An14g03420 |
| Metalloendopeptidase WSS1 (*S. cerevisiae* S288C) | M80 | NP_012002.1 | [^88^](#_ENREF_88) | An01g05470 |
| AN1049_2 (*A. nidulans* FGSC A4) | M80 | EAA66167.1 | [^88^](#_ENREF_88) | An08g05390 |
| Zinc metalloproteinase C607.06c (*S. pombe* 972h-) | Ascomycolysins | Q9US12.1 | [^89^](#_ENREF_89) | An02g06910 |

**References**

1 ten Have, A. *et al.* The *Botrytis cinerea* aspartic proteinase family. *Fungal Genet. Biol.* **47**, 53-65 (2010).

2 Golde, T. E., Wolfe, M. S. & Greenbaum, D. C. Signal peptide peptidases: A family of intramembrane-cleaving proteases that cleave type 2 transmembrane proteins. *Semin. Cell Dev. Biol.* **20**, 225-230 (2009).

3 Sasaki, H. *et al.* The crystal structure of an intermediate dimer of aspergilloglutamic peptidase that mimics the enzyme-activation product complex produced upon autoproteolysis. *J. Biochem.* **152**, 45-52 (2012).

4 Seemuller, E. *et al.* Proteasome from *Thermoplasma acidophilum*: a threonine protease. *Science* **268**, 579-582 (1995).

5 O'Farrell, P. A. & Joshua-Tor, L. Mutagenesis and crystallographic studies of the catalytic residues of the papain family protease bleomycin hydrolase: new insights into active-site structure. *Biochem. J.* **401**, 421-428 (2007).

6 Futai, E., Kubo, T., Sorimachi, H., Suzuki, K. & Maeda, T. Molecular cloning of PalBH, a mammalian homologue of the *Aspergillus* atypical calpain PalB. *BBA-Mol. Cell Res.* **1517**, 316-319 (2001).

7 Johnston, S. C., Larsen, C. N., Cook, W. J., Wilkinson, K. D. & Hill, C. P. Crystal structure of a deubiquitinating enzyme (human UCH-L3) at 1.8 Å resolution. *EMBO J.* **16**, 3787-3796 (1997).

8 Meyer, U., Benghezal, M., Imhof, I. & Conzelmann, A. Active site determination of Gpi8p, a caspase-related enzyme required for glycosylphosphatidylinositol anchor addition to proteins. *Biochemistry* **39**, 3461-3471 (2000).

9 Wong, A. H. H., Yan, C. Y. & Shi, Y. G. Crystal structure of the yeast metacaspase Yca1. *J. Biol. Chem.* **287**, 29251-29259 (2012).

10 Singleton, M. R., Isupov, M. N. & Littlechild, J. A. X-ray structure of pyrrolidone carboxyl peptidase from the hyperthermophilic archaeon *Thermococcus litoralis*. *Struct. Fold. Des.* **7**, 237-244 (1999).

11 Papa, F. R. & Hochstrasser, M. The yeast *DOA4* gene encodes a deubiquitinating enzyme related to a product of the human tre-2 oncogene. *Nature* **366**, 313-319 (1993).

12 Kim, K. I. *et al.* A new SUMO-1-specific protease, SUSP1, that is highly expressed in reproductive organs. *J. Biol. Chem.* **275**, 14102-14106 (2000).

13 Andres, G., Alejo, A., Simon-Mateo, C. & Salas, M. L. African swine fever virus protease, a new viral member of the SUMO-1-specific protease family. *J. Biol. Chem.* **276**, 780-787 (2001).

14 May, G. S., McGoldrick, C. A., Holt, C. L. & Denison, S. H. The *bimB3* mutation of *Aspergillus nidulans* uncouples DNA replication from the completion of mitosis. *J. Biol. Chem.* **267**, 15737-15743 (1992).

15 Maruyama, T. & Noda, N. N. Autophagy-regulating protease Atg4: structure, function, regulation and inhibition. *J. Antibiot.* **71**, 72-78 (2017).

16 Halio, S. B., Blumentals, I. I., Short, S. A., Merrill, B. M. & Kelly, R. M. Sequence, expression in *Escherichia coli*, and analysis of the gene encoding a novel intracellular protease (PfpI) from the hyperthermophilic archaeon *Pyrococcus furiosus*. *J. Bacteriol.* **178**, 2605-2612 (1996).

17 Shan, T. L. *et al.* Partial molecular cloning, characterization, and analysis of the subcellular localization and expression patterns of the porcine *OTUB1* gene. *Mol. Biol. Rep.* **36**, 1573-1577 (2009).

18 Desjardins, C. A. *et al.* Comparative genomic analysis of human fungal pathogens causing paracoccidioidomycosis. *Plos Genet.* **7**, e1002345 (2011).

19 Bailey-Elkin, B. A., van Kasteren, P. B., Snijder, E. J., Kikkert, M. & Mark, B. L. Viral OTU deubiquitinases: A structural and functional comparison. *Plos Pathogens* **10**, e1003894 (2014).

20 Schuhmann, H., Huesgen, P. F. & Adamska, I. The family of Deg/HtrA proteases in plants. *BMC Plant Biol.* **12**, 52 (2012).

21 Frederick, G. D., Rombouts, P. & Buxton, F. P. Cloning and characterisation of *pepC*, a gene encoding a serine protease from *Aspergillus niger*. *Gene* **125**, 57-64 (1993).

22 Jarai, G., Kirchherr, D. & Buxton, F. P. Cloning and characterization of the *pepD* gene of *Aspergillus niger* which codes for a subtilisin-like protease. *Gene* **139**, 51-57 (1994).

23 Jalving, R., van de Vondervoort, P. J. I., Visser, J. & Schaap, P. J. Characterization of the kexin-like maturase of *Aspergillus niger*. *Appl. Environ. Microbiol.* **66**, 363-368 (2000).

24 Doumas, A., van den Broek, P., Affolter, M. & Monod, M. Characterization of the prolyl dipeptidyl peptidase gene (*dppIV*) from the koji mold *Aspergillus oryzae*. *Appl. Environ. Microbiol.* **64**, 4809-4815 (1998).

25 Maeda, H. *et al.* Three extracellular dipeptidyl peptidases found in *Aspergillus oryzae* show varying substrate specificities. *Appl. Microbiol. Biotechnol.* **100**, 4947-4958 (2016).

26 Morita, H. *et al.* Molecular cloning of *ocpO* encoding carboxypeptidase O of *Aspergillus oryzae* IAM2640. *Biosci. Biotechnol. Biochem.* **74**, 1000-1006 (2010).

27 Tang, X. L., Lu, X. F., Wu, Z. M., Zheng, R. C. & Zheng, Y. G. Biocatalytic production of (S)-2-aminobutanamide by a novel D-aminopeptidase from *Brucella* sp. with high activity and enantioselectivity. *J. Biotechnol.* **266**, 20-26 (2018).

28 El Bakkouri, M. *et al.* Structural insights into the inactive subunit of the apicoplast-localized caseinolytic protease complex of *Plasmodium falciparum*. *J. Biol. Chem.* **288**, 1022-1031 (2013).

29 Chich, J. F. *et al.* Purification, crystallization, and preliminary X-ray analysis of PepX, an X-prolyl dipeptidyl aminopeptidase from *Lactococcus lactis*. *Proteins* **23**, 278-281 (1995).

30 Chich, J. F., Chapot-Chartier, M. P., Ribadeau-Dumas, B. & Gripon, J. C. Identification of the active site serine of the X-prolyl dipeptidyl aminopeptidase from *Lactococcus lactis*. *FEBS Lett.* **314**, 139-142 (1992).

31 Rigolet, P., Mechin, I., Delage, M. M. & Chich, J. F. The structural basis for catalysis and specificity of the X-prolyl dipeptidyl aminopeptidase from *Lactococcus lactis*. *Structure* **10**, 1383-1394 (2002).

32 Mayo, B. *et al.* Molecular cloning and sequence analysis of the X-prolyl dipeptidyl aminopeptidase gene from *Lactococcus lactis* subsp. *cremoris*. *Appl. Environ. Microbiol.* **57**, 38-44 (1991).

33 Xie, F. *et al.* The Lon protease homologue LonA, not LonC, contributes to the stress tolerance and biofilm formation of *Actinobacillus pleuropneumoniae*. *Microb. Pathogenesis* **93**, 38-43 (2016).

34 Nunnari, J., Fox, T. D. & Walter, P. A mitochondrial protease with two catalytic subunits of nonoverlapping specificities. *Science* **262**, 1997-2004 (1993).

35 Edens, L. *et al.* Extracellular prolyl endoprotease from *Aspergillus niger* and its use in the debittering of protein hydrolysates. *J. Agr. Food Chem.* **53**, 7950-7957 (2005).

36 Benoit, I. *et al.* Spatial differentiation of gene expression in *Aspergillus niger* colony grown for sugar beet pulp utilization. *Sci. Rep-UK.* **5**, 13592 (2015).

37 Kang, C., Yu, X. W. & Xu, Y. Gene cloning and enzymatic characterization of an endoprotease Endo-Pro-*Aspergillus niger*. *J. Ind. Microbiol. Biotechnol.* **40**, 855-864 (2013).

38 Kang, C., Yu, X. W. & Xu, Y. A codon-optimized endoprotease Endo-Pro-*Aspergillus niger*: Over expression and high-density fermentation in *Pichia pastoris*. *J. Mol. Catal. B-Enzym.* **104**, 64-69 (2014).

39 Kubota, K., Tanokura, M. & Takahashi, K. Purification and characterization of a novel prolyl endopeptidase from *Aspergillus niger*. *P. Jpn. Acad. B-Phys.* **81**, 447-453 (2005).

40 Li, N. A., Wu, J. M., Zhang, L. F., Zhang, Y. Z. & Feng, H. Characterization of a unique proline iminopeptidase from white-rot basidiomycetes *Phanerochaete chrysosporium*. *Biochimie* **92**, 779-788 (2010).

41 Basten, D. E., Moers, A. P., Ooyen, A. J. & Schaap, P. J. Characterisation of *Aspergillus niger* prolyl aminopeptidase. *Mol. Genet. Genomics* **272**, 673-679 (2005).

42 Naffin-Olivos, J. L. *et al.* Structure determination of *Mycobacterium tuberculosis* serine protease Hip1 (Rv2224c). *Biochemistry* **56**, 2304-2314 (2017).

43 Klein, J. R., Schmidt, U. & Plapp, R. Cloning, heterologous expression, and sequencing of a novel proline iminopeptidase gene, *pepI*, from *Lactobacillus delbrueckii* subsp. *lactis* DSM 7290. *Microbiology* **140 ( Pt 5)**, 1133-1139 (1994).

44 Lee, B. R. *et al.* Aorsin, a novel serine proteinase with trypsin-like specificity at acidic pH. *Biochem. J.* **371**, 541-548 (2003).

45 Reichard, U. *et al.* Sedolisins, a new class of secreted proteases from *Aspergillus fumigatus* with endoprotease or tripeptidyl-peptidase activity at acidic pHs. *Appl. Environ. Microbiol.* **72**, 1739-1748 (2006).

46 Wu, Z. *et al.* Structural analysis of a rhomboid family intramembrane protease reveals a gating mechanism for substrate entry. *Nat. Struct. Mol. Biol.* **13**, 1084-1091 (2006).

47 Lemberg, M. K. Sampling the membrane: function of rhomboid-family proteins. *Trends Cell Biol* **23**, 210-217 (2013).

48 García-Alvarez, N., Cueva, R. & Suárez-Rendueles, P. Molecular cloning of soluble aminopeptidases from *Saccharomyces cerevisiae*. Sequence analysis of aminopeptidase yscII, a putative zinc-metallopeptidase. *FEBS J.* **202**, 993–1002 (1991).

49 Basten, D. E. J. W., Visser, J. & Schaap, P. J. Lysine aminopeptidase of *Aspergillus niger*. *Microbiol-SGM* **147**, 2045-2050 (2001).

50 Ibrahim-Granet, O. & D'Enfert, C. The *Aspergillus fumigatus mepB* gene encodes an 82 kDa intracellular metalloproteinase structurally related to mammalian thimet oligopeptidases. *Microbiology* **143**, 2247-2253 (1997).

51 Ruf, A., Stihle, M., Benz, J., Schmidt, M. & Sobek, H. Structure of gentlyase, the neutral metalloprotease of *Paenibacillus polymyxa*. *Acta Crystallogr. D Biol. Crystallogr.* **69**, 24-31 (2013).

52 Muller, D. *et al.* The collagenase gene family in humans consists of at least four members. *Biochem. J.* **253**, 187-192 (1988).

53 Tarentino, A. L., Quinones, G., Grimwood, B. G., Hauer, C. R. & Plummer, T. H., Jr. Molecular cloning and sequence analysis of flavastacin: an O-glycosylated prokaryotic zinc metalloendopeptidase. *Arch. Biochem. Biophys.* **319**, 281-285 (1995).

54 Igarashi, T., Araki, S., Mori, H. & Takeda, S. Crystal structures of catrocollastatin/VAP2B reveal a dynamic, modular architecture of ADAM/adamalysin/reprolysin family proteins. *FEBS Lett.* **581**, 2416-2422 (2007).

55 Otero, A. *et al.* The novel structure of a cytosolic M14 metallocarboxypeptidase (CCP) from *Pseudomonas aeruginosa*: a model for mammalian CCPs. *FASEB J.* **26**, 3754-3764 (2012).

56 Alper, B. J., Rowse, J. W. & Schmidt, W. K. Yeast Ste23p shares functional similarities with mammalian insulin-degrading enzymes. *Yeast* **26**, 595-610 (2009).

57 Desy, S., Schneider, A. & Mani, J. *Trypanosoma brucei* has a canonical mitochondrial processing peptidase. *Mol. Biochem. Parasit.* **185**, 161-164 (2012).

58 Stahl, A. *et al.* Isolation and identification of a novel mitochondrial metalloprotease (PreP) that degrades targeting presequences in plants. *J. Biol. Chem.* **277**, 41931-41939 (2002).

59 Chaikuad, A. *et al.* Structure of human aspartyl aminopeptidase complexed with substrate analogue: insight into catalytic mechanism, substrate specificity and M18 peptidase family. *BMC Struct. Biol.* **12**, 14 (2012).

60 Keynan, S., Hooper, N. M. & Turner, A. J. Identification by site-directed mutagenesis of three essential histidine residues in membrane dipeptidase, a novel mammalian zinc peptidase. *Biochem. J.* **326**, 47-51 (1997).

61 Oku, T. *et al.* Purification and identification of two carnosine-cleaving enzymes, carnosine dipeptidase I and Xaa-methyl-His dipeptidase, from Japanese eel (*Anguilla japonica*). *Biochimie* **94**, 1281-1290 (2012).

62 Girish, T. S., Vivek, B., Colaco, M., Misquith, S. & Gopal, B. Structure of an amidohydrolase, SACOL0085, from methicillin-resistant *Staphylococcus aureus* COL. *Acta Crystallogr. F* **69**, 103-108 (2013).

63 Jamdar, S. N. *et al.* The members of M20D peptidase subfamily from *Burkholderia cepacia*, *Deinococcus radiodurans* and *Staphylococcus aureus* (HmrA) are carboxydipeptidases, primarily specific for Met-X dipeptides. *Arch. Biochem. Biophys.* **587**, 18-30 (2015).

64 Okumura, N., Tamura, J. & Takao, T. Evidence for an essential role of intradimer interaction in catalytic function of carnosine dipeptidase II using electrospray-ionization mass spectrometry. *Protein Sci.* **25**, 511-522 (2016).

65 Otani, H., Okumura, N., Hashida-Okumura, A. & Nagai, K. Identification and characterization of a mouse dipeptidase that hydrolyzes L-carnosine. *J. Biochem.* **137**, 167-175 (2005).

66 K, H. *et al.* Eukaryotic GCP1 is a conserved mitochondrial protein required for progression of embryo development beyond the globular stage in *Arabidopsis thaliana*. *Biochem. J.* **423**, 333-341 (2009).

67 Ikeda, S., Uda, H. & Seki, Y. Enzyme activity of O-sialoglycoprotein endopeptidase (OSGEP) of *Saccharomyces cerevisiae* Kae1p is essential for growth, but the bacterial and mammalian OSGEP homologs can not complement the yeast KAE1 null mutation. *Bulletin of the Okayama University of Science A Natural Science* **44**, 27-31 (2008).

68 Munkhjargal, T. *et al.* Molecular and biochemical characterization of methionine aminopeptidase of *Babesia bovis as* a potent drug target. *Vet. Parasitol.* **221**, 14-23 (2016).

69 Kgosisejo, O., Chen, J. A., Grochulski, P. & Tanaka, T. Crystallographic structure of recombinant *Lactococcus lactis* prolidase to support proposed structure-function relationships. *BBA-Mol. Cell Res.* **1865**, 473-480 (2017).

70 Maras, B. *et al.* Aminopeptidase from *Streptomyces griseus*. *Eur. J. Biochem.* **236**, 843–846 (1996).

71 Fundoiano-Hershcovitz, Y. *et al.* Identification of the catalytic residues in the double-zinc aminopeptidase from *Streptomyces griseus*. *FEBS Lett.* **571**, 192-196 (2004).

72 Rawlings, N. D. & Barrett, A. J. Structure of membrane glutamate carboxypeptidase. *BBA-Mol. Cell Res.* **1339**, 247-252 (1997).

73 Tsukamoto, T., Wozniak, K. M. & Slusher, B. S. Progress in the discovery and development of glutamate carboxypeptidase II inhibitors. *Drug Discov. Today* **12**, 767-776 (2007).

74 Monod, M. *et al.* Aminopeptidases and dipeptidyl-peptidases secreted by the dermatophyte *Trichophyton rubrum*. *Microbiology* **151**, 145-155 (2005).

75 Schilling, S. *et al.* Isolation, catalytic properties, and competitive inhibitors of the zinc-dependent murine glutaminyl cyclase. *Biochemistry* **44**, 13415-13424 (2005).

76 Fernandez, D., Russi, S., Vendrell, J., Monod, M. & Pallares, I. A functional and structural study of the major metalloprotease secreted by the pathogenic fungus *Aspergillus fumigatus*. *Acta Crystallogr. D-Biol. Crystallogr.* **69**, 1946-1957 (2013).

77 Yoshizawa, S. *et al.* Functional characterization of flavobacteria rhodopsins reveals a unique class of light-driven chloride pump in bacteria. *Proc. Natl. Acad. Sci. USA.* **111**, 6732-6737 (2014).

78 Klanner, C., Prokisch, H. & Langer, T. MAP-1 and IAP-1, two novel AAA proteases with catalytic sites on opposite membrane surfaces in mitochondrial inner membrane of *Neurospora crassa*. *Mol. Biol. Cell* **12**, 2858-2869 (2001).

79 Ming-Hua, S. *et al.* Purification, characterization, and cloning of fibrinolytic metalloprotease from *Pleurotus ostreatus* mycelia. *J. Microbiol. Biotechnol.* **17**, 1271-1283 (2007).

80 Pryor, E. E. *et al.* Structure of the integral membrane protein CAAX protease Ste24p. *Science* **339**, 1600-1604 (2013).

81 Jajcanin-Jozic, N., Deller, S., Pavkov, T., Macheroux, P. & Abramic, M. Identification of the reactive cysteine residues in yeast dipeptidyl peptidase III. *Biochimie* **92**, 89-96 (2010).

82 Jajcanin-Jozic, N., Macheroux, P. & Abramic, M. Yeast ortholog of peptidase family M49: the role of invariant Glu(461) and Tyr(327). *Croat. Chem. Acta* **85**, 535-540 (2012).

83 Panschin, I. *et al.* Comparing polysaccharide decomposition between the type strains *Gramella echinicola* KMM 6050(T) (DSM 19838(T)) and *Gramella portivictoriae* UST040801-001(T) (DSM 23547(T)), and emended description of *Gramella echinicola* Nedashkovskaya et al. 2005 emend. Shahina et al. 2014 and *Gramella portivictoriae* Lau et al. 2005. *Stand. Genomic Sci.* **11** (2016).

84 Verma, R. *et al.* Role of Rpn11 metalloprotease in deubiquitination and degradation by the 26S proteasome. *Science* **298**, 611-615 (2002).

85 Davies, C. W., Paul, L. N. & Das, C. Mechanism of recruitment and activation of the endosome-associated deubiquitinase AMSH. *Biochemistry* **52**, 7818-7829 (2013).

86 Zeng, X. M., Neupert, W. & Tzagoloff, A. The metalloprotease encoded by *ATP23* has a dual function in processing and assembly of subunit 6 of mitochondrial ATPase. *Mol. Biol. Cell* **18**, 617-626 (2007).

87 Manolaridis, I. *et al.* Mechanism of farnesylated CAAX protein processing by the intramembrane protease Rce1. *Nature* **504**, 301-305 (2013).

88 Iyer, L. M., Koonin, E. V. & Aravind, L. Novel predicted peptidases with a potential role in the ubiquitin signaling pathway. *Cell Cycle* **3**, 1440-1450 (2004).

89 Gomis-Ruth, F. X. Structural aspects of the metzincin clan of metalloendopeptidases. *Mol. Biotechnol.* **24**, 157-202 (2003).
