# Supplementary material for: Bioinformatic mapping of a more precise Aspergillus niger degradome
Source: Sci Rep. 2021 Jan 12;11:693. doi: 10.1038/s41598-020-80028-3 (PMC7804941; doi:10.1038/s41598-020-80028-3)
Supplement: Supplementary file 5 — Supplementary Table S4. [file 41598_2020_80028_MOESM5_ESM.doc]

**Supplementary Table S4.** Proteases from *A. niger* strains that have been characterized by molecular, biochemical and/or omics techniques

| **Serial Number** | **Old locus tag** | **Gene** | **Description** | **Identity ^a^** | **Family** | **References** |
| --- | --- | --- | --- | --- | --- | --- |
| **Molecularly and/or biochemically characterized proteases** | | | | | | |
| 1 | An15g06280 | *pepAa* | Aspergillopepsin A-like aspartic endopeptidase | 100% | Aspartic | [^1^](#_ENREF_1) |
| 2 | An01g00370 | *pepAb/pepN* | Aspartic endopeptidase (AP1) | 100% | Aspartic |  |
| 3 | An12g03300* | *pepAc/protG* | Aspartic protease pepAc | 100% | Aspartic |  |
| 4 | An04g01440* | *pepAd* | Aspartic-type endopeptidase ctsD | 100% | Aspartic | [^1^](#_ENREF_1) |
| 5 | An02g07210* | *pepE* | Intracellular protease | 99% | Aspartic |  |
| 6 | An14g04710* | *pepA/pep1* | Aspartic proteinase, proctase B, aspergillopepsin I | 99% | Aspartic | [^7-10^](#_ENREF_7) |
| 7 | An01g00530* | *pepB/protI/prtA* | Protease-A;  aspergilloglutamic peptidase and histidine-specific protease | 100% | Glutamic |  |
| 8 | An01g08530* | *kexB* | Dibasic-processing endoprotease | 99% | Serine | [^20-22^](#_ENREF_20) |
| 9 | An02g11420 | *dapB* | Dipeptidyl aminopeptidase type IV | 99% | Serine | [^23^](#_ENREF_23) |
| 10 | An04g02850 | *protC/apsC* | X-Pro dipeptidyl-peptidase IV | 99% | Serine | [^24^](#_ENREF_24) |
| 11 | An07g03880* | *pepC* | Serine proteinase | 99% | Serine | [^25^](#_ENREF_25) |
| 12 | An02g04690* | *cpd-I* | Serine carboxypeptidase | 98% | Serine |  |
| 13 | An07g08030* | *pepF/cpd-II* | Serine carboxypeptidase | 100% | Serine | [^26-29^](#_ENREF_26) |
| 14 | An08g04490* | *protA/epr* | Endoprotease | 93% | Serine | [^30-34^](#_ENREF_30) |
| 15 | An08g08750* | *cpyA/cpyD* | Carboxypeptidase Y | 94% | Serine |  |
| 16 | An09g03780* | *pepD* | Subtilisin-like serine protease | 99% | Serine | [^37^](#_ENREF_37) |
| 17 | An11g04730 | *papA* | Prolyl aminopeptidase | 98% | Serine | [^38^](#_ENREF_38) |
| 18 | An14g02470* |  | Tripeptidyl-peptidase sed2 | 100% | Serine |  |
| 19 | An04g03930 | *apsA* | Aminopeptidase | 98% | Metallo | [^45^](#_ENREF_45) |
| **Proteases identified by genomic, transcriptomic, proteomic or secretomic methods** | | | | | | |
| 1 | An11g00310 |  | Aspartic protease | 100% | Aspartic | [^46^](#_ENREF_46) |
| 2 | An11g09170 |  | Aspartic protease | 100% | Aspartic | [^31^](#_ENREF_31) |
| 3 | An15g07700 | *protD* | Acid protease A | 100% | Glutamic | [^4^](#_ENREF_4) |
| 4 | An01g04680 | *palB* | Calpain-like protease palB | 100% | Cysteine |  |
| 5 | An01g08470 |  | Ubiquitin carboxyl-terminal hydrolase creB | 100% | Cysteine |  |
| 6 | An03g03810 |  | X-Pro dipeptidyl-peptidase (S15 family) protein | 100% | Cysteine | [^31^](#_ENREF_31) |
| 7 | An09g04470 | *casB* | Metacaspase | 100% | Cysteine | [^48^](#_ENREF_48) |
| 8 | An09g05480 |  | Ubiquitin hydrolase | 100% | Cysteine | [^49^](#_ENREF_49) |
| 9 | An11g11320 | *atg4* | Cysteine protease | 100% | Cysteine | [^48^](#_ENREF_48) |
| 10 | An13g01190 |  | Ulp1 protease | 100% | Cysteine | [^46^](#_ENREF_46) |
| 11 | An18g05760 | *casA* | Metacaspase | 100% | Cysteine | [^48^](#_ENREF_48) |
| 12 | An01g00560 |  | Signal peptidase complex catalytic subunit SEC11C | 100% | Serine | [^50^](#_ENREF_50) |
| 13 | An02g01550 |  | Allergen Asp f 15 | 100% | Serine |  |
| 14 | An03g05200 | *protF* | Carboxypeptidase Y family secreted protease | 100% | Serine |  |
| 15 | An06g00190 |  | Tripeptidyl-peptidase sed2 | 100% | Serine |  |
| 16 | An08g04640 | *protB* | Serine endopeptidase | 100% | Serine |  |
| 17 | An08g00430 | *kex1* | Carboxypeptidase alpha-factor processing | 100% | Serine | [^52^](#_ENREF_52) |
| 18 | An09g00950 |  | D-stereospecific aminopeptidase | 100% | Seine |  |
| 19 | An11g01110 |  | Aorsin | 100% | Serine | [^46^](#_ENREF_46) |
| 20 | An12g04700 | *dpp5* | Dipeptidyl-peptidase 5 | 100% | Serine | [^31^](#_ENREF_31) |
| 21 | An13g03240 |  | Dipeptidyl aminopeptidase/acylaminoacyl peptidase | 100% | Serine | [^31^](#_ENREF_31) |
| 22 | An14g02150 |  | Carboxypeptidase | 100% | Serine |  |
| 23 | An16g02250 |  | Tripeptidyl-peptidase sed3 | 100% | Serine | [^46^](#_ENREF_46) |
| 24 | An16g08150 |  | Dipeptidyl-peptidase 5 | 100% | Serine |  |
| 25 | An16g09010 | *protH* | Carboxypeptidase I | 100% | Serine | [^4^](#_ENREF_4) |
| 26 | An17g00760 |  | Carboxypeptidase S1 | 100% | Serine | [^31^](#_ENREF_31) |
| 27 | An01g11340 |  | Methionine aminopeptidase 1 | 100% | Metallo |  |
| 28 | An02g11940 |  | Aspartyl aminopeptidase | 100% | Metallo |  |
| 29 | An02g13740 |  | Putative Gly-X carboxypeptidase precursor | 100% | Metallo | [^31^](#_ENREF_31) |
| 30 | An03g01660 | *ape3* | Vacuolar aminopeptidase Y | 100% | Metallo |  |
| 31 | An04g02320 |  | Metalloprotease 1 MP1 | 100% | Metallo | [^46^](#_ENREF_46) |
| 32 | An04g02880 |  | Glutaminyl cyclase | 100% | Metallo | [^31^](#_ENREF_31) |
| 33 | An04g05530 |  | Adamalysin | 100% | Metallo | [^46^](#_ENREF_46) |
| 34 | An05g00070 |  | Leukotriene A-4 hydrolase | 100% | Metallo | [^46^](#_ENREF_46) |
| 35 | An07g01970 |  | Metallopeptidase MepB | 100% | Metallo | [^46^](#_ENREF_46) |
| 36 | An09g06250 |  | Aspartyl aminopeptidase | 100% | Metallo |  |
| 37 | An09g06800 | *protE/apsB* | Leucyl aminopeptidase | 100% | Metallo | [^48^](#_ENREF_48) |
| 38 | An11g06960 | *pepP* | Cell cycle kinase | 100% | Metallo | [^54^](#_ENREF_54) |
| 39 | An11g11180 |  | Glutamate carboxypeptidase-like protein | 100% | Metallo | [^49^](#_ENREF_49) |
| 40 | An14g00620 | *lap1* | Leucine aminopeptidase | 100% | Metallo |  |
| 41 | An18g06210 |  | Peptidase | 100% | Metallo | [^31^](#_ENREF_31) |

**^a^** The identity between the reported gene and the corresponding gene in *A. niger* CBS 513.88 or ATCC 1015

* Secreted proteases that have been predicted by the six predictors.

**References**

1 Wang, Y. C. *et al.* Isolation of four pepsin-like protease genes from *Aspergillus niger* and analysis of the effect of disruptions on heterologous laccase expression. *Fungal Genetics and Biology* **45**, 17-27 (2008).

2 Burggraaf, A. M., Punt, P. J. & Ram, A. F. J. The unconventional secretion of PepN is independent of a functional autophagy machinery in the filamentous fungus *Aspergillus niger*. *FEMS Microbiology Letters* **363**, fnw152 (2016).

3 Morya, V. K., Yadav, S., Kim, E. K. & Yadav, D. *In silico* characterization of alkaline proteases from different species of *Aspergillus*. *Appl. Biochem. Biotechnol.* **166**, 243-257 (2012).

4 Levin, A. M. *et al.* Spatial differentiation in the vegetative mycelium of *Aspergillus niger*. *Eukaryot. Cell* **6**, 2311-2322 (2007).

5 Jarai, G., van den Hombergh, H. & Buxton, F. P. Cloning and characterization of the *pepE* gene of *Aspergillus niger* encoding a new aspartic protease and regulation of *pepE* and *pepC*. *Gene* **145**, 171-178 (1994).

6 Buxton, F., Jarai, G. & Visser, J. *Aspergillus niger* vacuolar aspartyl protease. US005674728A (1997).

7 Lu, J. F., Inoue, H., Kimura, T., Makabe, O. & Takahashi, K. Molecular cloning of a cDNA for proctase B from *Aspergillus niger* var. *macrosporus* and sequence comparison with other aspergillopepsins I. *Biosci Biotechnol Biochem* **59**, 954-955 (1995).

8 Mattern, I. E. *et al.* Isolation and characterization of mutants of *Aspergillus niger* deficient in extracellular proteases. *Mol. Gen. Genet.* **234**, 332-336 (1992).

9 Yin, L. J., Hsu, T. H. & Jiang, S. T. Characterization of acidic protease from *Aspergillus niger* BCRC 32720. *Journal of Agricultural & Food Chemistry* **61**, 662-666 (2013).

10 Purushothaman, K., Bhat, S. K., Singh, S. A., Marathe, G. K. & Appu Rao, A. R. G. Aspartic protease from *Aspergillus niger*: Molecular characterization and interaction with pepstatin A. *Int. J. Biol. Macromol.* **139**, 199-212 (2019).

11 Shi, J. *et al.* Properties of hemoglobin decolorized with a histidine-specific protease. *J. Food Sci.* **80**, E1202-1208 (2015).

12 Bruins, M. J., Edens, L. & Leneke, N. Use of *Aspergillus niger* aspergilloglutamic peptidase to improve animal performance. US20160302446A1 (2016).

13 Inoue, H., Kimura, T., Makabe, O. & Takahashi, K. The gene and deduced protein sequences of the zymogen of *Aspergillus niger* acid proteinase A. *J. Biol. Chem.* **266**, 19484-19489 (1991).

14 Takahashi, K. *et al.* The primary structure of *Aspergillus niger* acid proteinase A. *J. Biol. Chem.* **266**, 19480-19483 (1991).

15 Takahashi, K. Proteinase A from *Aspergillus niger*. *Methods Enzymol.* **248**, 146-155 (1995).

16 Iio, K. & Yamasaki, M. Specificity of acid proteinase A from *Aspergillus niger* var. *macrosporus* towards B-chain of performic acid oxidized bovine insulin. *BBA-Mol. Cell Res.* **429**, 912-924 (1976).

17 Takahashi, K. *et al.* Structure and function of a pepstatin-insensitive acid proteinase from *Aspergillus niger* var. *macrosporus*. *Adv. Exp. Med. Biol.* **306**, 203-211 (1991).

18 Sasaki, H. *et al.* The crystal structure of an intermediate dimer of aspergilloglutamic peptidase that mimics the enzyme-activation product complex produced upon autoproteolysis. *J. Biochem.* **152**, 45-52 (2012).

19 Yabuki, Y., Kubota, K., Kojima, M., Inoue, H. & Takahashi, K. Identification of a glutamine residue essential for catalytic activity of aspergilloglutamic peptidase by site-directed mutagenesis. *FEBS Lett.* **569**, 161-164 (2004).

20 Jalving, R., van de Vondervoort, P. J. I., Visser, J. & Schaap, P. J. Characterization of the kexin-like maturase of *Aspergillus niger*. *Appl. Environ. Microbiol.* **66**, 363-368 (2000).

21 Heerikhuisen, M. *et al.* Novel means of transformation of fungi and their use for heterologous protein production. AU 782116 B2 (2005).

22 Punt, P. J. *et al.* The role of the *Aspergillus niger* furin-type protease gene in processing of fungal proproteins and fusion proteins - Evidence for alternative processing of recombinant (fusion-) proteins. *J. Biotechnol.* **106**, 23-32 (2003).

23 Jalving, R., Godefrooij, J., ter Veen, W. J., van Ooyen, A. J. J. & Schaap, P. J. Characterisation of the *Aspergillus niger dapB* gene, which encodes a novel fungal type IV dipeptidyl aminopeptidase. *Molecular Genetics and Genomics* **273**, 319-325 (2005).

24 Basten, D. E. J. W., Dekker, P. J. T. & Schaap, P. J. Aminopeptidase C of *Aspergillus niger* is a novel phenylalanine aminopeptidase. *Appl. Environ. Microbiol.* **69**, 1246-1250 (2003).

25 Frederick, G. D., Rombouts, P. & Buxton, F. P. Cloning and characterisation of *pepC*, a gene encoding a serine protease from *Aspergillus niger*. *Gene* **125**, 57-64 (1993).

26 Dal, D. F., Ribadeaudumas, B. & Breddam, K. Purification and characterization of two serine carboxypeptidases from *Aspergillus niger* and their use in C-terminal sequencing of proteins and peptide synthesis. *Appl. Environ. Microbiol.* **58**, 2144-2152 (1992).

27 Svendsen, I. & Dal Degan, F. The amino acid sequences of carboxypeptidases I and II from *Aspergillus niger* and their stability in the presence of divalent cations. *BBA-Protein Struct. Mol. Enzymol.* **1387**, 369-377 (1998).

28 van den Hombergh, J. P., Jarai, G., Buxton, F. P. & Visser, J. Cloning, characterization and expression of *pepF*, a gene encoding a serine carboxypeptidase from *Aspergillus niger*. *Gene* **151**, 73-79 (1994).

29 Krishnan, S. & Vijayalakshmi, M. A. Purification of an acid protease and a serine carboxypeptidase from *Aspergillus niger* using metal-chelate affinity chromatography. *J. Chromatogr.* **329**, 165-170 (1985).

30 Edens, L. *et al.* Extracellular prolyl endoprotease from *Aspergillus niger* and its use in the debittering of protein hydrolysates. *J Agric Food Chem* **53**, 7950-7957 (2005).

31 Benoit, I. *et al.* Spatial differentiation of gene expression in *Aspergillus niger* colony grown for sugar beet pulp utilization. *Sci. Rep-UK.* **5**, 13592 (2015).

32 Kang, C., Yu, X. W. & Xu, Y. Gene cloning and enzymatic characterization of an endoprotease Endo-Pro-*Aspergillus niger*. *J. Ind. Microbiol. Biotechnol.* **40**, 855-864 (2013).

33 Kang, C., Yu, X. W. & Xu, Y. A codon-optimized endoprotease Endo-Pro-*Aspergillus niger*: Over expression and high-density fermentation in *Pichia pastoris*. *J. Mol. Catal. B-Enzym.* **104**, 64-69 (2014).

34 Kubota, K., Tanokura, M. & Takahashi, K. Purification and characterization of a novel prolyl endopeptidase from *Aspergillus niger*. *P. Jpn. Acad. B-Phys.* **81**, 447-453 (2005).

35 Yaver, D. S. & Thompson, S. A. Gene encoding carboxypeptidase of *Aspergillus niger*. US5939305 A (1997).

36 Thompson, S. A. & Yaver, D. S. Gene encoding carboxypeptidase of *Aspergillus niger*. US5693510 (1995).

37 Jarai, G., Kirchherr, D. & Buxton, F. P. Cloning and characterization of the *pepD* gene of *Aspergillus niger* which codes for a subtilisin-like protease. *Gene* **139**, 51-57 (1994).

38 Basten, D. E., Moers, A. P., Ooyen, A. J. & Schaap, P. J. Characterisation of *Aspergillus niger* prolyl aminopeptidase. *Mol Genet Genomics* **272**, 673-679 (2005).

39 Holm, K. A., Rasmussen, G., Halkier, T. & Lehmbeck, J. Tripeptidyl aminopeptidase. US5821104 (1998).

40 Reichard, U. *et al.* Sedolisins, a new class of secreted proteases from *Aspergillus fumigatus* with endoprotease or tripeptidyl-peptidase activity at acidic pHs. *Appl. Environ. Microbiol.* **72**, 1739-1748 (2006).

41 Adav, S. S., Li, A. A., Manavalan, A., Punt, P. & Sze, S. K. Quantitative iTRAQ secretome analysis of *Aspergillus niger* reveals novel hydrolytic enzymes. *J. Proteome Res.* **9**, 3932-3940 (2010).

42 Braaksma, M., Martens-Uzunova, E. S., Punt, P. J. & Schaap, P. J. An inventory of the *Aspergillus niger* secretome by combining in silico predictions with shotgun proteomics data. *BMC Genomics* **11**, 584 (2010).

43 Krijgsheld, P. *et al.* Spatially resolving the secretome within the mycelium of the cell factory *Aspergillus niger*. *J. Proteome Res.* **11**, 2807-2818 (2012).

44 de Souza, W. R. *et al.* Transcriptome analysis of *Aspergillus niger* grown on sugarcane bagasse. *Biotechnol. Biofuels* **4**, 40 (2011).

45 Basten, D. E. J. W., Visser, J. & Schaap, P. J. Lysine aminopeptidase of *Aspergillus niger*. *Microbiol-SGM* **147**, 2045-2050 (2001).

46 Schäpe, P. *et al.* Updating genome annotation for the microbial cell factory *Aspergillus niger* using gene co-expression networks. *Nucleic Acids Res.* **47**, 559-569 (2018).

47 Chen, Z. *et al.* Comparative genomic and transcriptomic analysis of *Wangiella dermatitidis*, A major cause of phaeohyphomycosis and a model black yeast human pathogen. *G3-Genes Genomes Genetics* **4**, 561-578 (2014).

48 Nitsche, B. M., Jorgensen, T. R., Akeroyd, M., Meyer, V. & Ram, A. F. J. The carbon starvation response of *Aspergillus niger* during submerged cultivation: Insights from the transcriptome and secretome. *BMC Genomics* **13**, 380 (2012).

49 Manzanares-Miralles, L. *et al.* Quantitative proteomics reveals the mechanism and consequence of gliotoxin-mediated dysregulation of the methionine cycle in *Aspergillus niger*. *J. Proteomics* **131**, 149-162 (2016).

50 Guillemette, T. *et al.* Genomic analysis of the secretion stress response in the enzyme-producing cell factory *Aspergillus niger*. *BMC Genomics* **8**, 158 (2007).

51 Krijgsheld, P. Transcriptome analysis of zones of colonies of the Δ*flbA* strain of *Aspergillus niger*. *Fungal Genomics & Biology* **3**, 1000109 (2013).

52 Ida, V. V. D. P. J., Jan, P. H. & Stanley, D. P. Process for preparing filamentous fungal strains having a sexual cycle and a process for preparing sexually crossed filamentous fungal strains. CA2789176 A1 (2011).

53 Kitoh, S. *et al.* 1-Acetyl-5-(4-fluoro-phen-yl)-2-sulfanyl-ideneimidazolidin-4-one. *Acta Crystallogr Sect E Struct Rep Online* **69**, o1699 (2013).

54 Jørgensen, T. R. *et al.* Transcriptomic insights into the physiology of *Aspergillus niger* approaching a specific growth rate of zero. *Appl. Environ. Microbiol.* **76**, 5344-5355 (2010).

55 Huang, W. Q. *et al.* The structure and enzyme characteristics of a recombinant leucine aminopeptidase rLap1 from *Aspergillus sojae* and its application in debittering. *Appl. Biochem. Biotechnol.* **177**, 190-206 (2015).
