# Supplementary material for: Bioinformatic mapping of a more precise Aspergillus niger degradome
Source: Sci Rep. 2021 Jan 12;11:693. doi: 10.1038/s41598-020-80028-3 (PMC7804941; doi:10.1038/s41598-020-80028-3)
Supplement: Supplementary file 6 — Supplementary Table S5. [file 41598_2020_80028_MOESM6_ESM.doc]

**Supplementary Table S5a. Reclassification of metallopeptidases based on active site architecture and overall fold similarity** [**^1^**](#_ENREF_1)

| **Tribe** ^a^ | **Clan (conserved motif)** ^a^ | **Family** ^a^ | **Members (subfamily)** |
| --- | --- | --- | --- |
| Inverzincins (HXXEH) | HXXEHX_76_EXXV/H+E |  | An07g06490 (M16A)  An16g01860 (M16A)  An01g12210 (M16B)  An04g01980 (M16B)  An08g04080 (M16B)  An09g06650 (M16B)  An04g02320 (M16C) |
| αβα-Exopeptidases | Aminoacylase-1 family ((S/G/A)HXDXV+P/GXXD+XEE+D/E+H) |  | An02g11940 (M18)  An09g06250 (M18)  An02g12680 (M20A)  An02g13740 (M20A)  An18g06210 (M20A)  An04g10270 (M20F)  An11g03000 (M20F)  An11g11180 (M20F)  An03g01660 (M28A)  An02g06300 (M28B)  An18g03980 (M28B)  An14g00620 (M28E)  An17g00390 (M28E)  An04g02880 (M28F)  An18g03780 (M28F)  An06g00780  An07g03400 |
|  | EEM2-MPs (EE) |  | An01g11610 (M20D)  An02g00990 (M20D)  An08g07280 (M20D)  An11g07760 (M20D)  An11g08890 (M20D)  An12g02360 (M20D)  An15g01800 (M20D)  An14g03420 (M79) |
|  | Funnelins (M14) (HXXE+R+NR+H+Y+E) | Subfamily A/B (M14A) | An12g04170 (M14A) |
| Zincins (HEXXH) | Metzincins (HEXXHXXGXXH/D+M) |  | An01g05470 (M80)  An08g05390 (M80) |
|  |  | Ascomycolysins | An02g06910 |
|  |  | Astacins | An15g00830 (M12A)  An14g01410 (M57) |
|  |  | Matrixins/Matrilysin | An12g02780 (M10A) |
|  |  | Adamalysins/ADAMs (M12B) | An04g05530 (M12B)  An15g03750 (M12B) |
|  |  | Pappalysins (M43B) | An07g10410 (M43B) |
|  | Gluzincins (HEXXH+E/HXXZ) | M48/M56 IMMPs (HEXXH+EXXA+N+H) | An04g01950 (M48A)  An04g07380 (M48C) |
|  |  | Dipeptidyl peptidase III (M49) (HEXXXH+EER/KXAE/D) | An01g02980 (M49)  An04g00410 (M49) |
|  |  | Thermolysins (M4) (HEXXH+NEXXS+Y+H) | An12g05900 (M4) |
|  |  | Fungalysins [^2^](#_ENREF_2) (HEYTH+YALESGGMGEGWSD+TYTSVNSLNAVHAIGTVWASILY) | An01g02070 (M36) |
|  |  | Cowrins (HEXXH+EXXS/G+H+Y/R+Y) | An07g00470 (M3A)  An07g01970 (M3A)  An11g05710 (M3A)  An15g02290 (M3A) |
|  |  | LA4H+M1+APs (HEXXH+NEXXT/A+GXMEN+Y) | An04g03930 (M1)  An05g00070 (M1)  An09g06800 (M1) |
|  | FtsH-like AAA MPs (HEXXH+E+ND+H_2_O) |  | An04g04970 (M41)  An07g07000 (M41) |

^a^ Putative catalytic residues, metal-binding residues and residues/molecules occupying the position of the Met-turn or Ser/Gly-turn beneath the metal sites are colored in pink, green and orange, respectively. Other residues involved in stabilization of the reaction intermediate, substrate binding, and/or catalysis are shown in black, except for X, which represents any amino acid and is here only used as a spacer within motifs.

Besides the 57 metallopeptidases listed in Supplementary Table S5A, there are also 23 enzymes that can not be assigned into the abovementioned clans or tribes. Among them, An05g00110 from family M76 and An06g01580 contain the conserved motif HEXXH, and they are thus zincins. The cDNA-derived amino acid sequence of An01g11740 (family M19) contains none of the major zinc peptidase motifs such as HEXXH or HXXEH [^3^](#_ENREF_3), but possesses a region (DHIMYIGNLIGFDH; residues 361-374) of close similarity with the crystallographically identified zinc-binding motif (DHTH) in D-alanyl-D-alanine-cleaving carboxypeptidase of *Streptomyces albus* G [^4^](#_ENREF_4) and the region (DHLDH) from the membrane dipeptidase from pig kidney cortex [^3^](#_ENREF_3). Thus, it’s a novel zincin. Other 20 enzymes from families M22, M24, M38 and M67 that can not be assigned into the above clans or tribes are listed below.

**Supplementary Table S5b.** Metallopeptidases that can not be assigned into the above tribes or clans

| **Family** | **Conserved motif** | **Members (Subfamily)** |
| --- | --- | --- |
| M22 (Glycoprotein endopeptidase) | HX(E/V/Q)XH+D+H | An07g03020 (M22)  An15g00900 (M22) |
| M24 (Methionyl aminopeptidase and Xaa-Pro dipeptidase) |  | An01g11340 (M24A) |
|  |  | An01g11360 (M24A) |
|  |  | An04g01330 (M24A) |
|  |  | An07g09120 (M24A) |
|  | HXXGHXXGX_3-8_H | An01g13040 (M24B)  An01g14920 (M24B)  An03g04230 (M24B)  An05g00050 (M24B)  An09g00700 (M24B)  An11g06960 (M24B) |
| M38 (Enzymes that are not provisionally identified) | HXH+K+H+H+D | An02g00090 (M38)  An11g05920 (M38)  An14g02080 (M38)  An14g03560 (M38)  An15g04370 (M38) |
| M67 (JAMM (Jab1/MPN/MOV34) family of deubiquitinating enzymes) | EX_n_HXHX_10_D | An07g07860 (M67A)  An07g10110 (M67A)  An02g12490 (M67C) |

**References**

1 Cerda-Costa, N. & Xavier Gomis-Ruth, F. Architecture and function of metallopeptidase catalytic domains. *Protein Sci.* **23**, 123-144 (2014).

2 Kolattukudy, P. E. & Sirakova, T. D. in *Handbook of Proteolytic Enzymes (Second Edition)* (eds Alan J. Barrett, Neil D. Rawlings, & J. Fred Woessner) 792-793 (Academic Press, 2004).

3 Keynan, S., Hooper, N. M. & Turner, A. J. Identification by site-directed mutagenesis of three essential histidine residues in membrane dipeptidase, a novel mammalian zinc peptidase. *Biochem. J.* **326**, 47-51 (1997).

4 Joris, B. *et al.* The complete amino acid sequence of the Zn^2+^-containing D-alanyl-D-alanine-cleaving carboxypeptidase of *Streptomyces albus* G. *FEBS J.* **130**, 53–69 (1983).
