# Supplementary material for: Bioinformatic mapping of a more precise Aspergillus niger degradome
Source: Sci Rep. 2021 Jan 12;11:693. doi: 10.1038/s41598-020-80028-3 (PMC7804941; doi:10.1038/s41598-020-80028-3)

**Supplementary Figure S1.** Phylogenetic relationships among aspartic (**a**), glutamic (**b**), threonine (**c**), cysteine (**d**), serine (**e**) and metallopeptidases (**f**) from *A. niger* CBS 513.88 and ATCC 1015 obtained by a Maximum Parsimony analysis using MEGA 6.0. Only bootstrap values higher than 50% are indicated for each branch. Triangles indicate genes (their names are in parentheses) that have been previously characterized by molecular and/or biochemical methods.


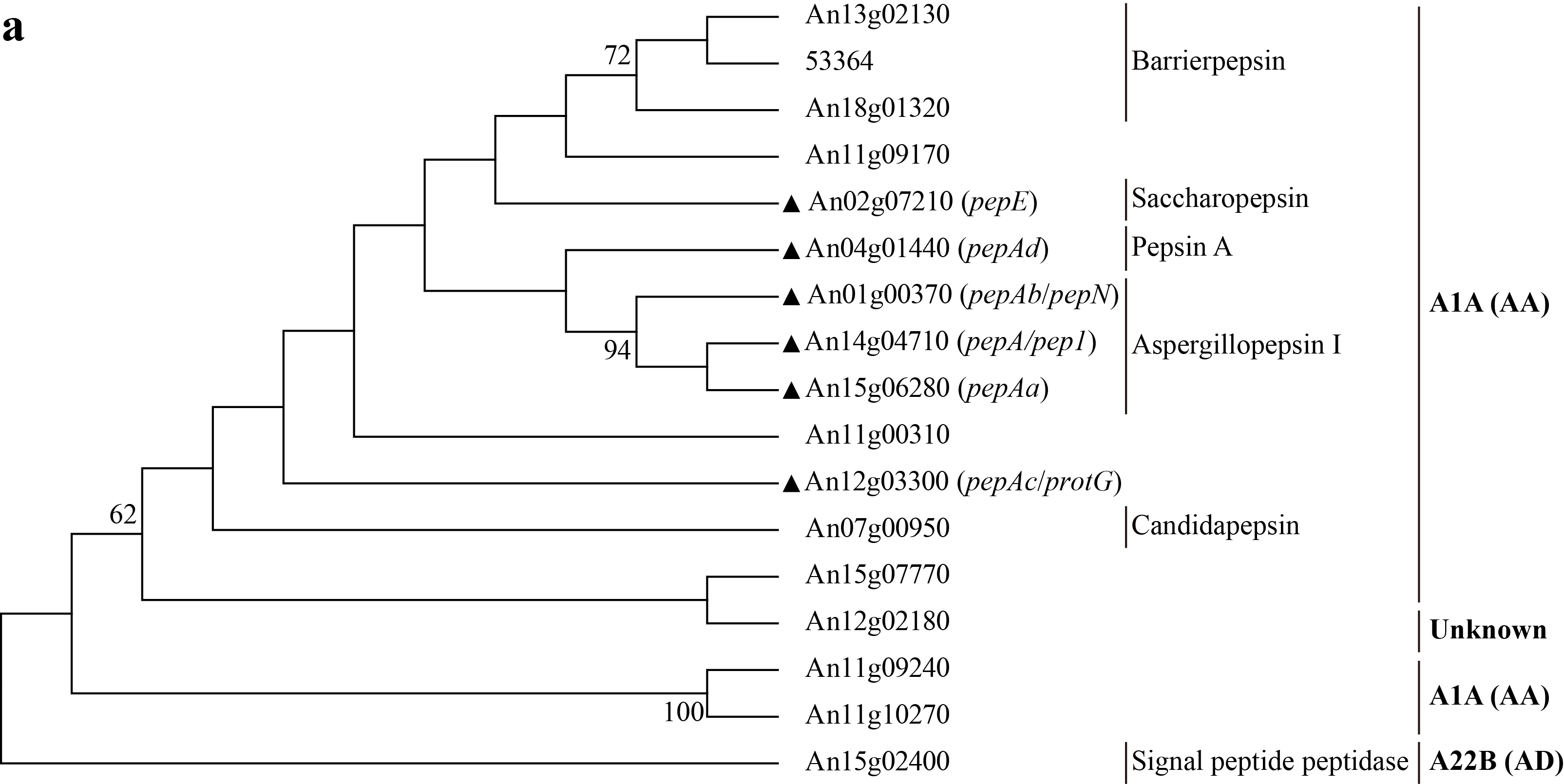


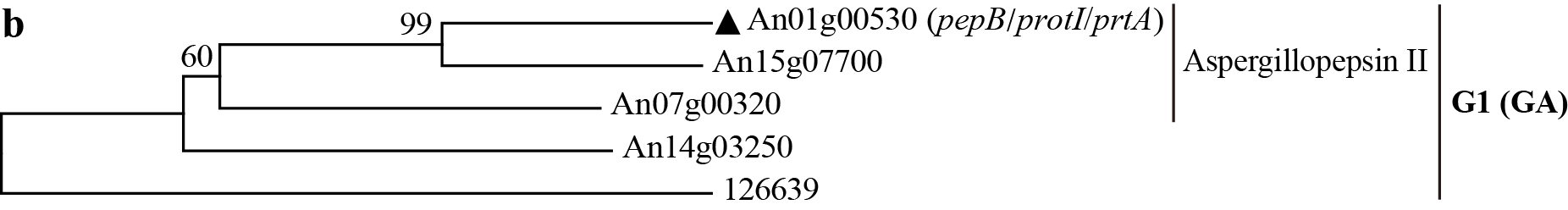


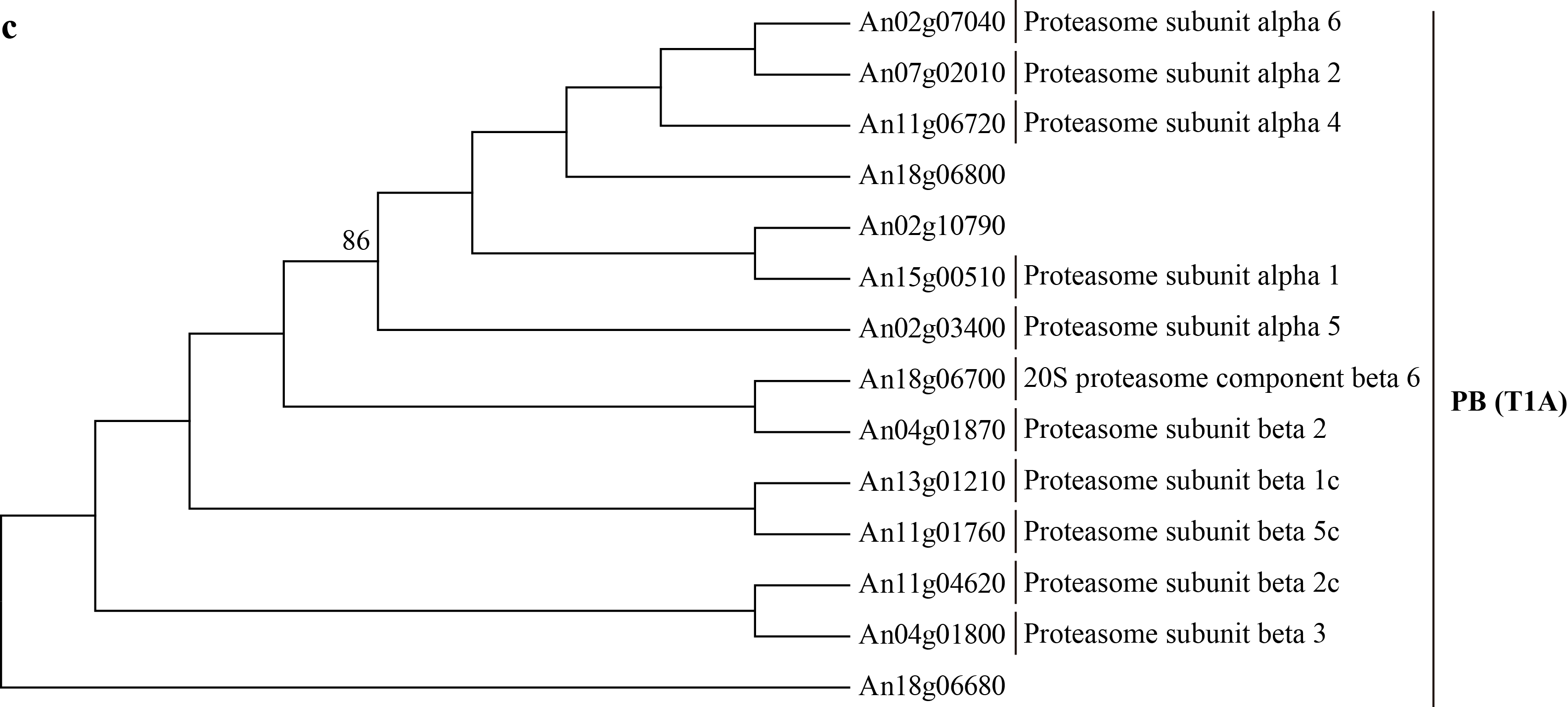


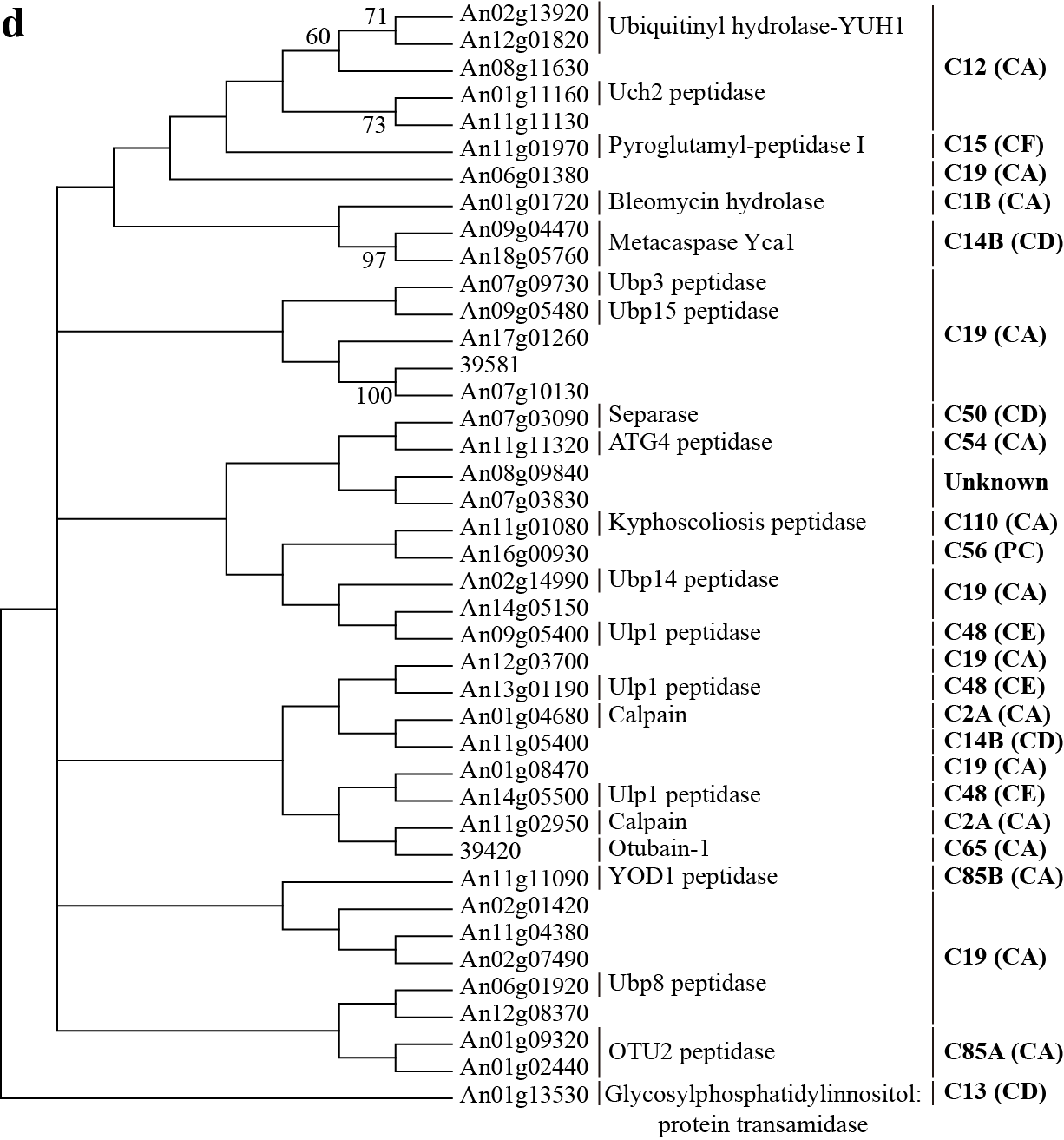


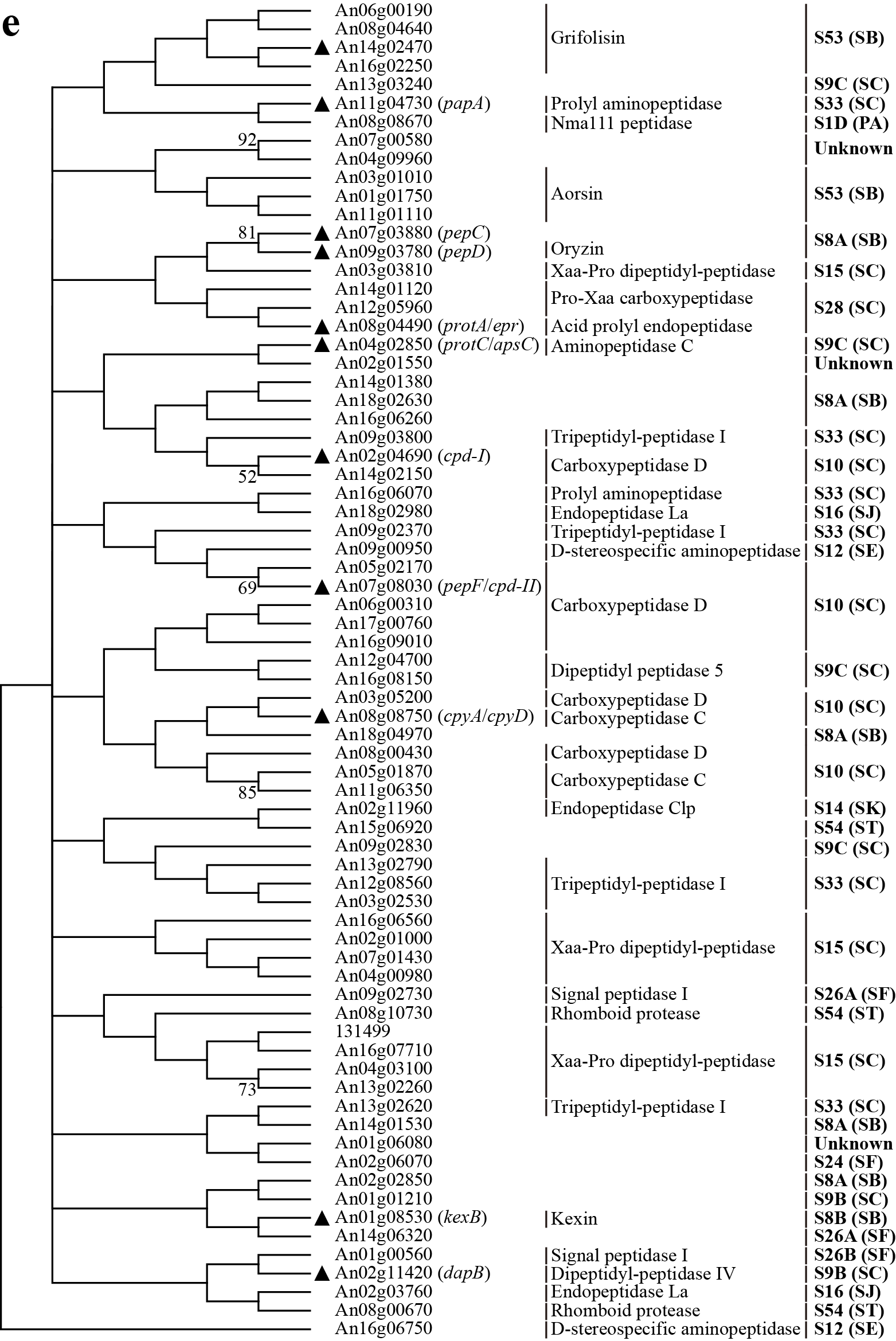


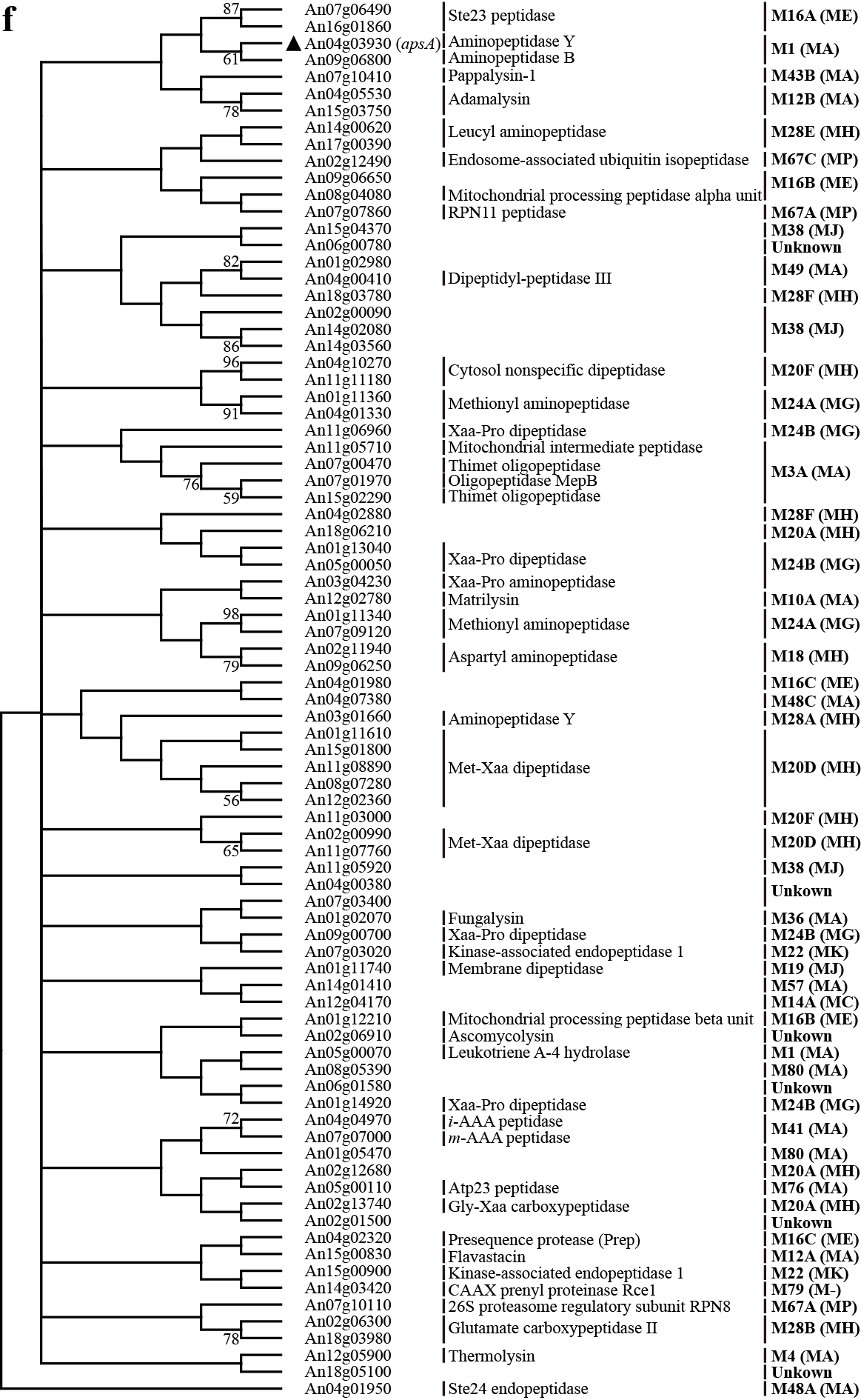

Supplement: Supplementary file 7 — Supplementary Figure S1. [file 41598_2020_80028_MOESM7_ESM.doc]
