# Supplementary material for: Bioinformatic mapping of a more precise Aspergillus niger degradome
Source: Sci Rep. 2021 Jan 12;11:693. doi: 10.1038/s41598-020-80028-3 (PMC7804941; doi:10.1038/s41598-020-80028-3)

**Supplementary Figure S2.** Multiple sequence alignment of aspartic (**a**), glutamic (**b**), threonine (**c**), cysteine (**d**), serine (**e**) and metalloproteases (**f**) from *A. niger* CBS 513.88 and ATCC 1015, respectively. Genes that have been previously characterized by molecular and/or biochemical methods are in bold. Putative signal peptides, active sites and metal-binding residues are highlighted in gray, purple and green, respectively. The possible proton acceptor (lysine residues) and two glycine-rich sequences (GlySerGly and SerGlyGly) that are conserved in active threonine proteases (**c**) and residues occupying the position of the Met-turn or Ser/Gly-turn beneath the metal sites of metallopeptidases (**f**) are colored in yellow and orange, respectively. Conserved motifs or cysteines are boxed.


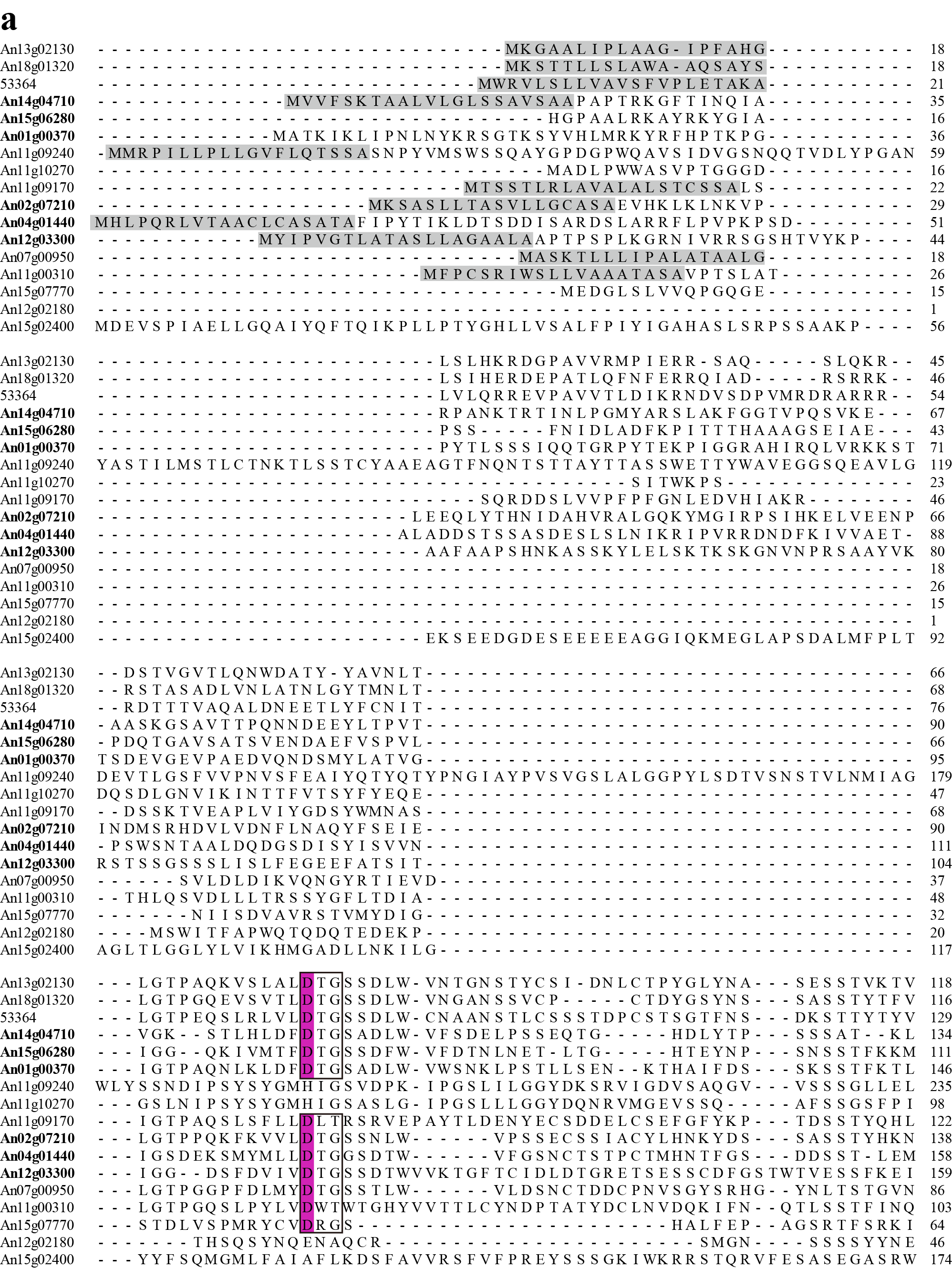


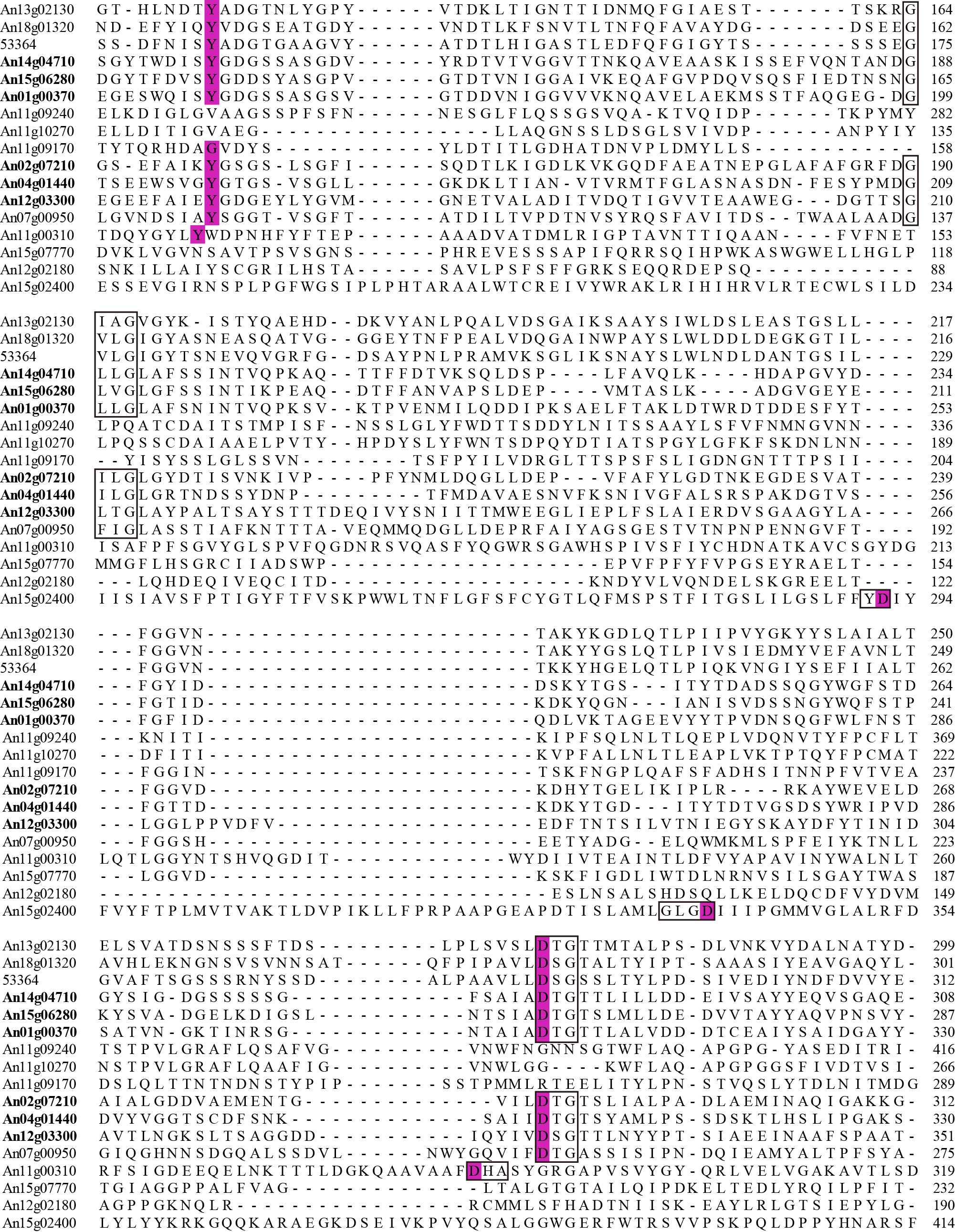


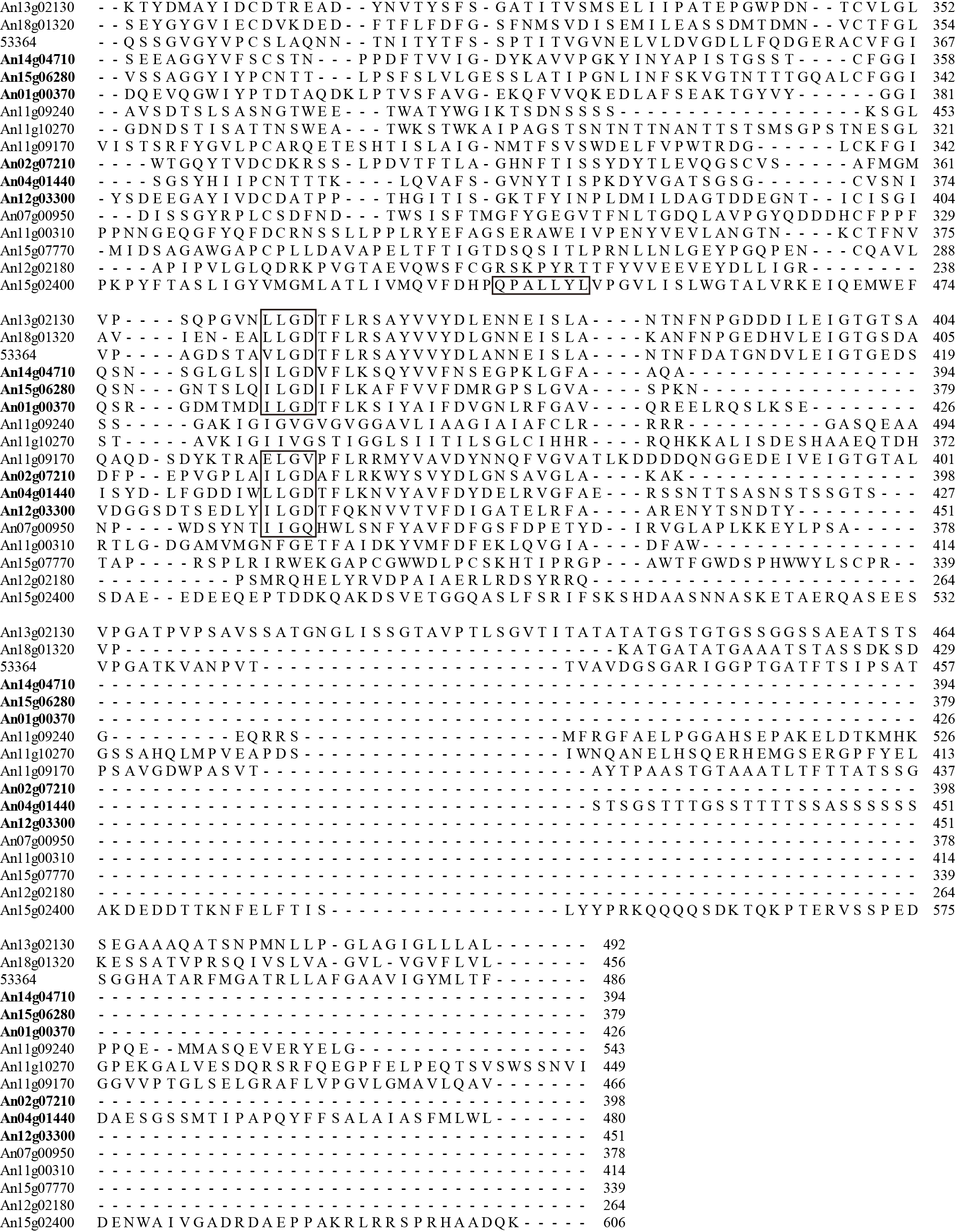


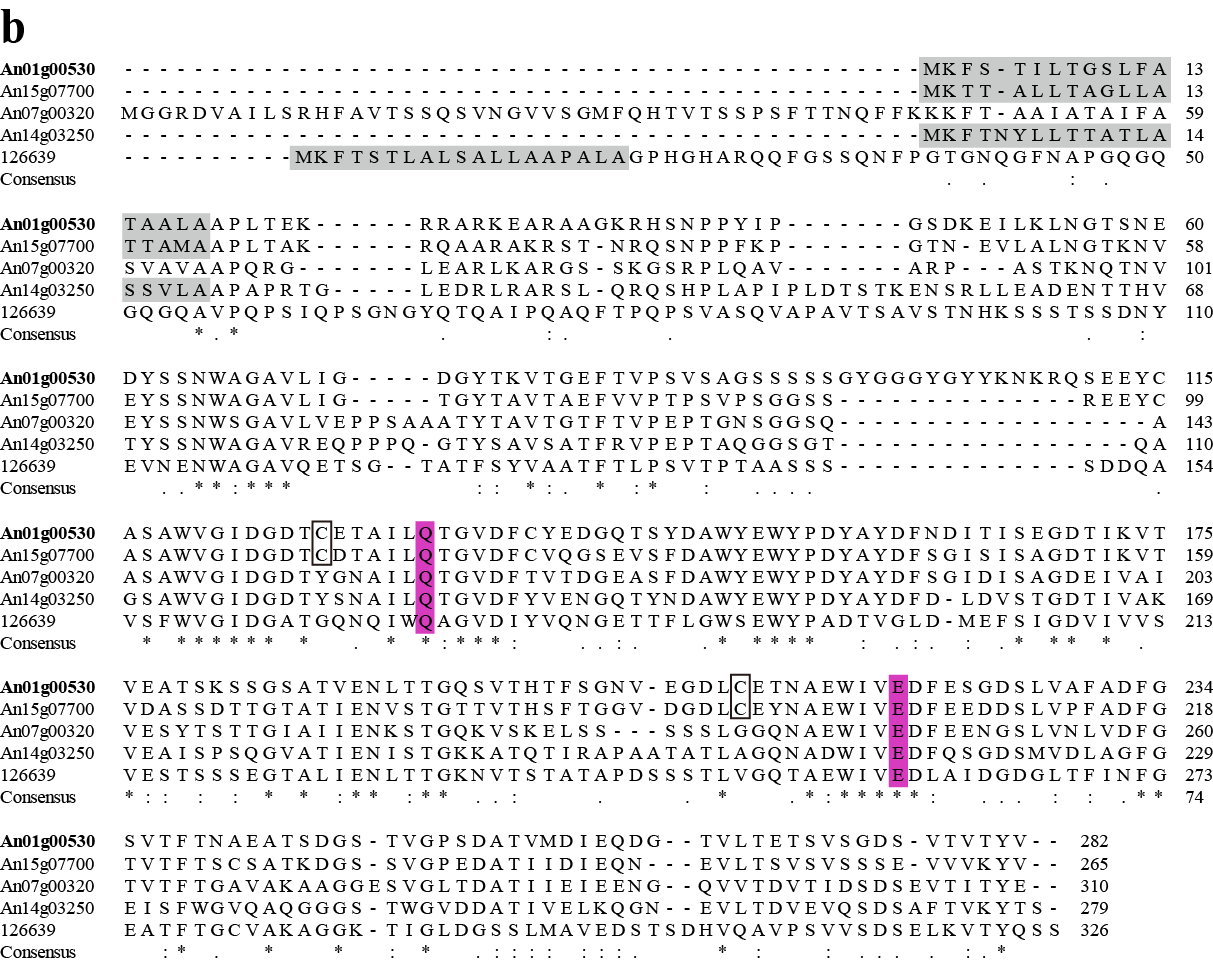


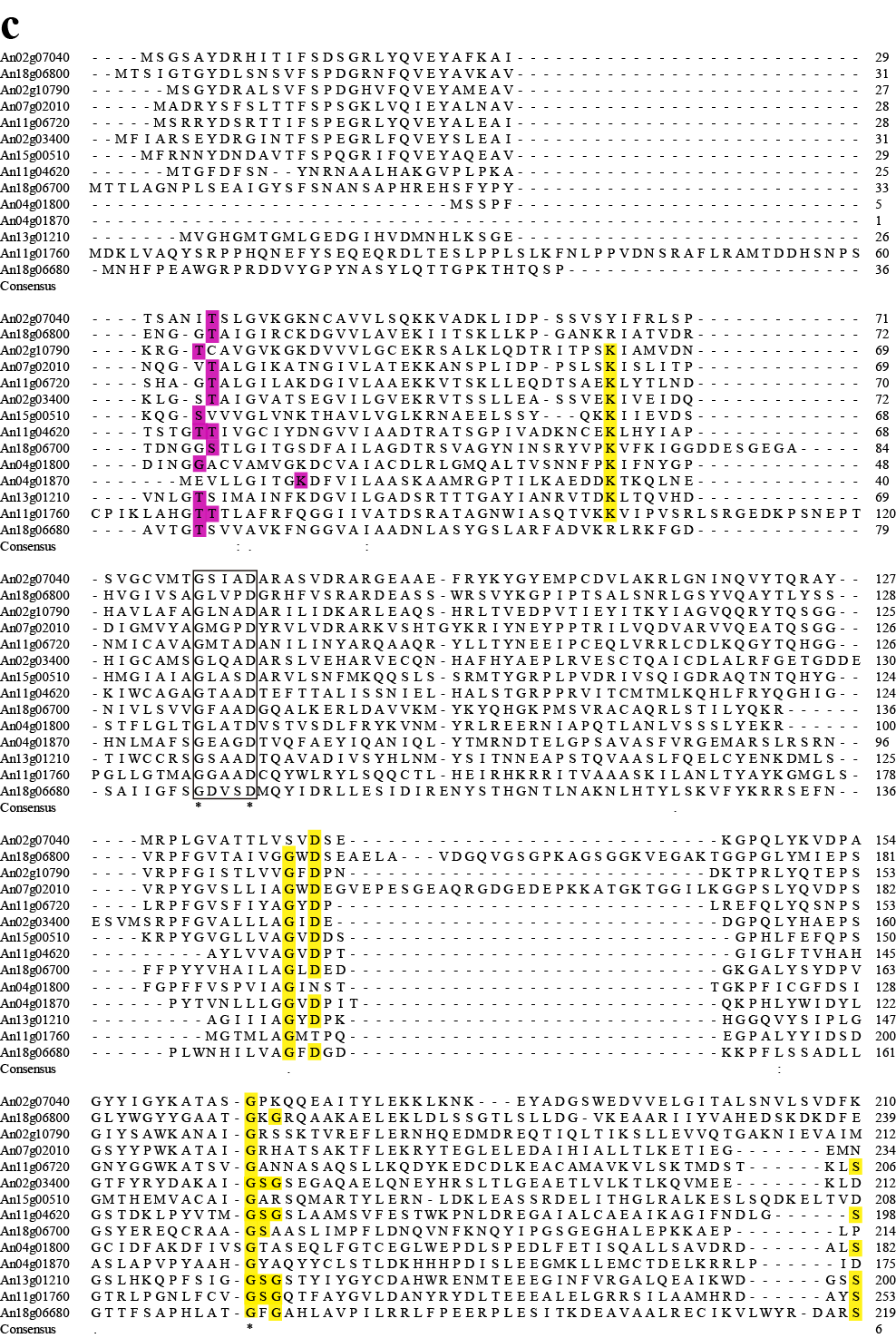


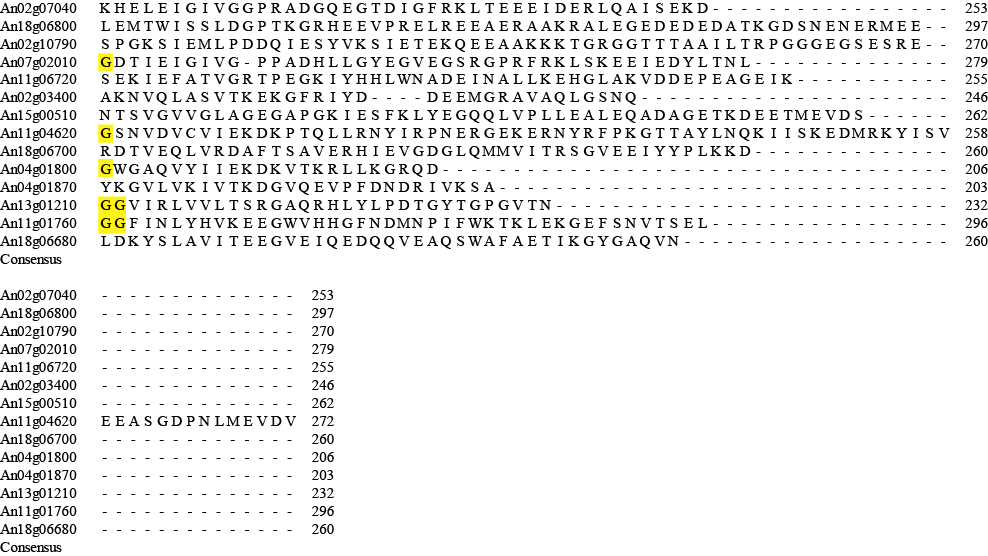


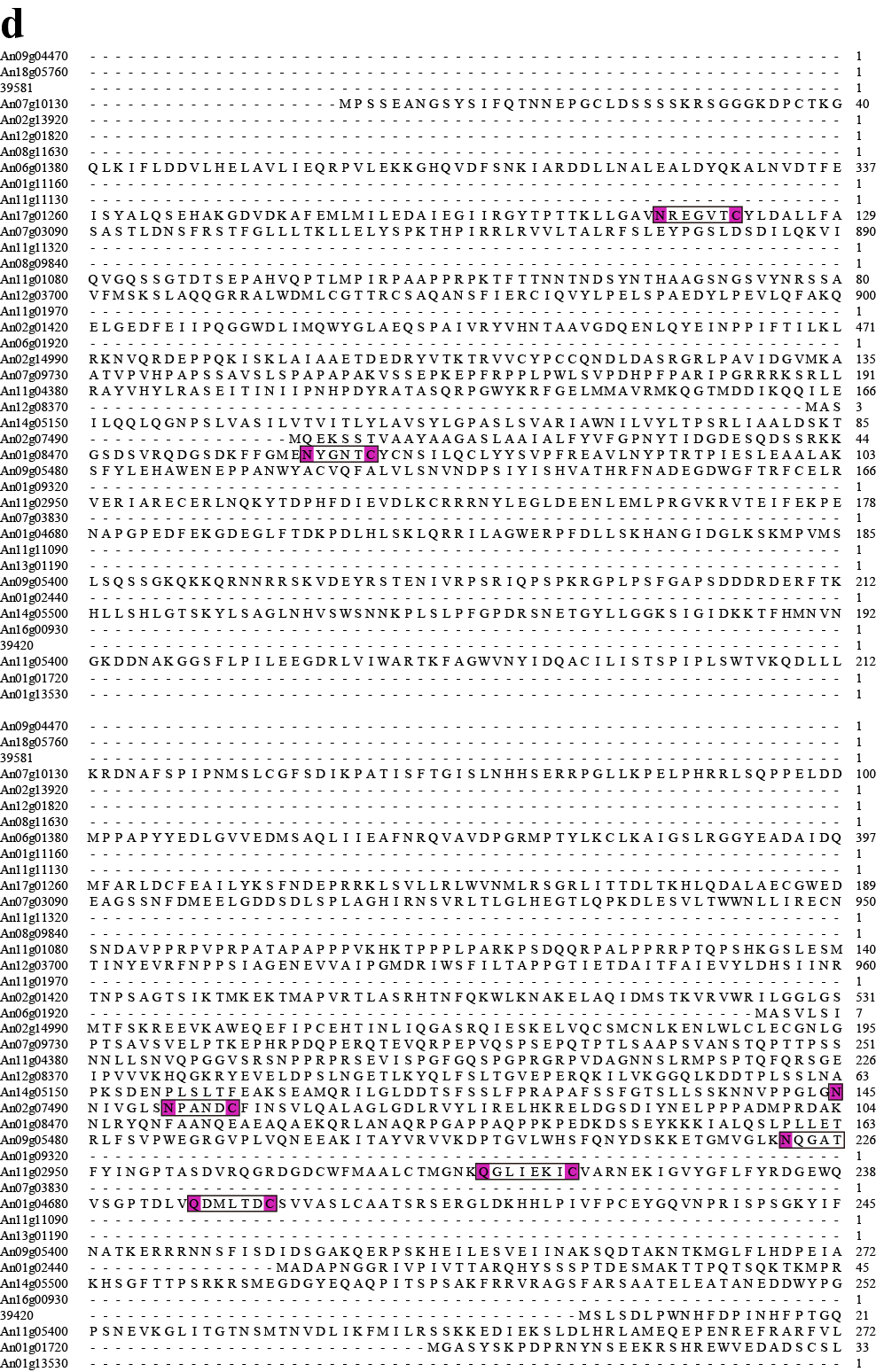


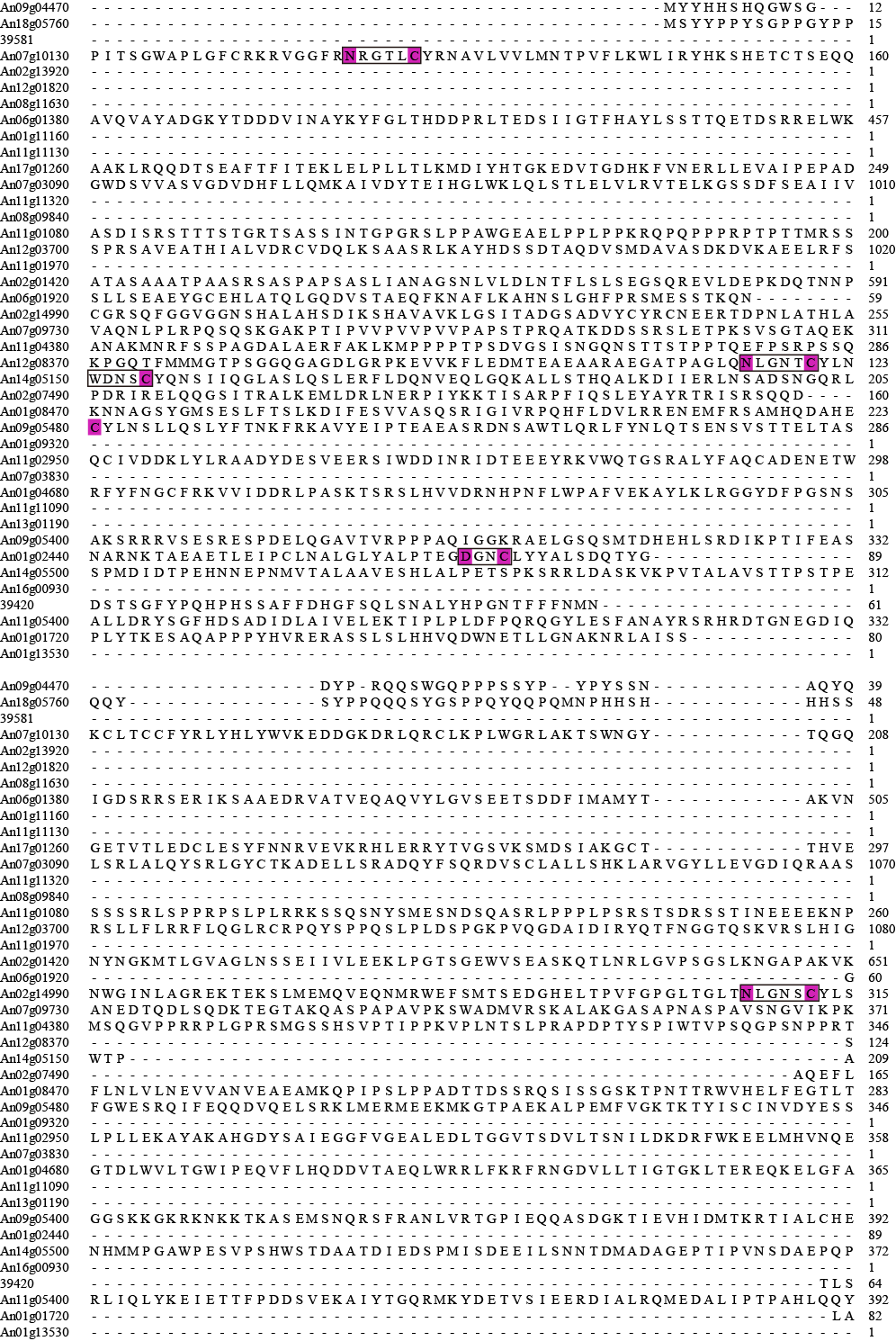


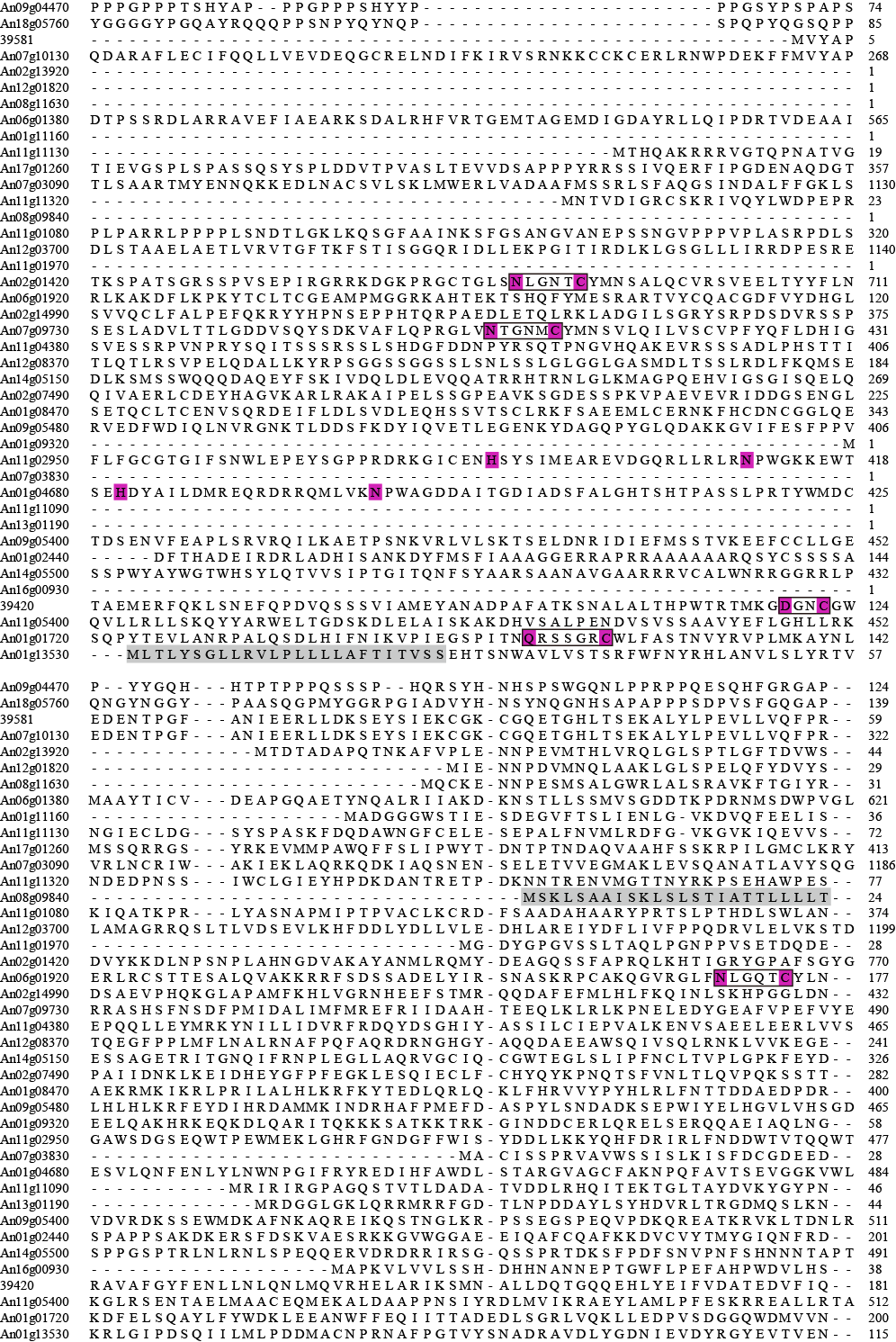


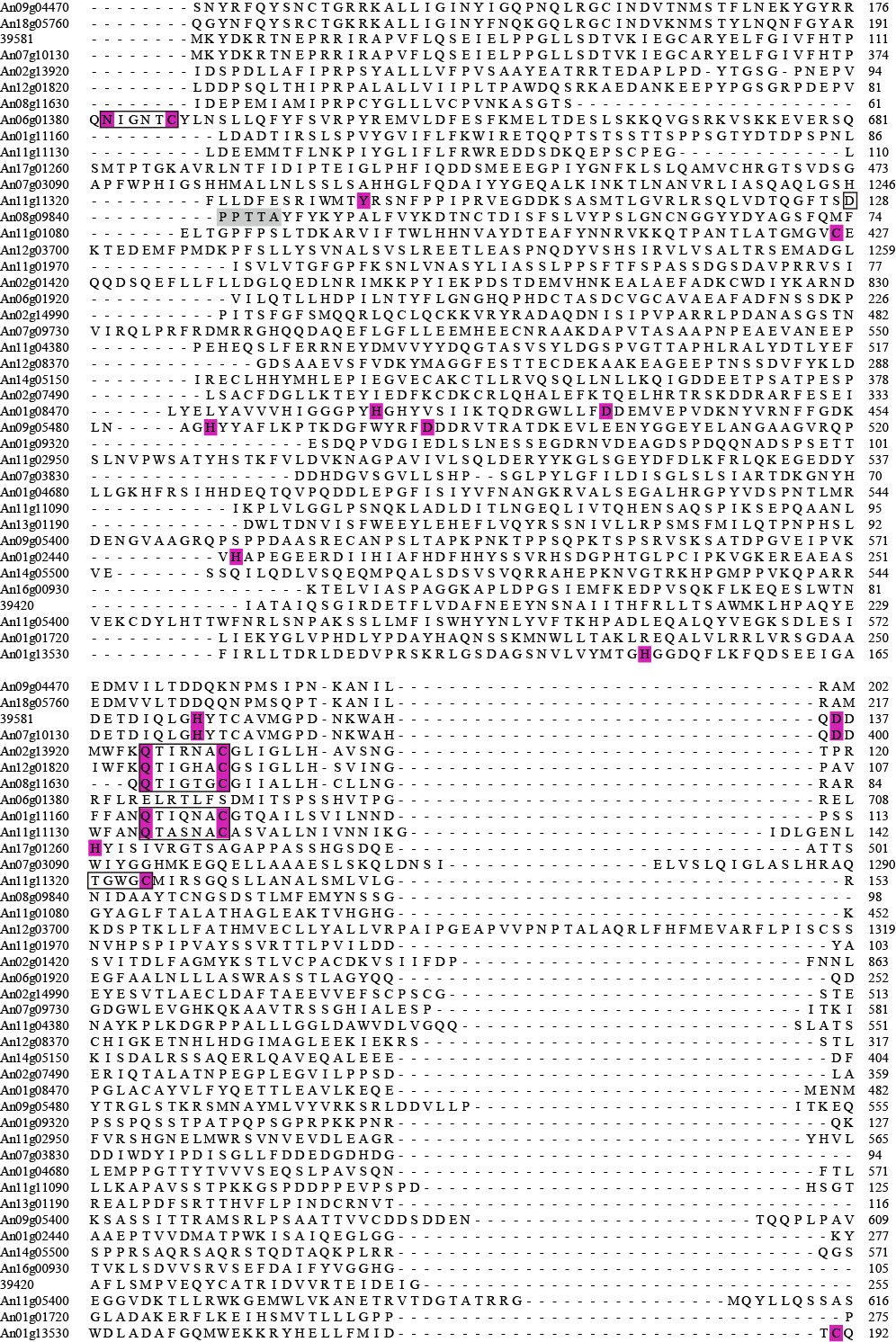


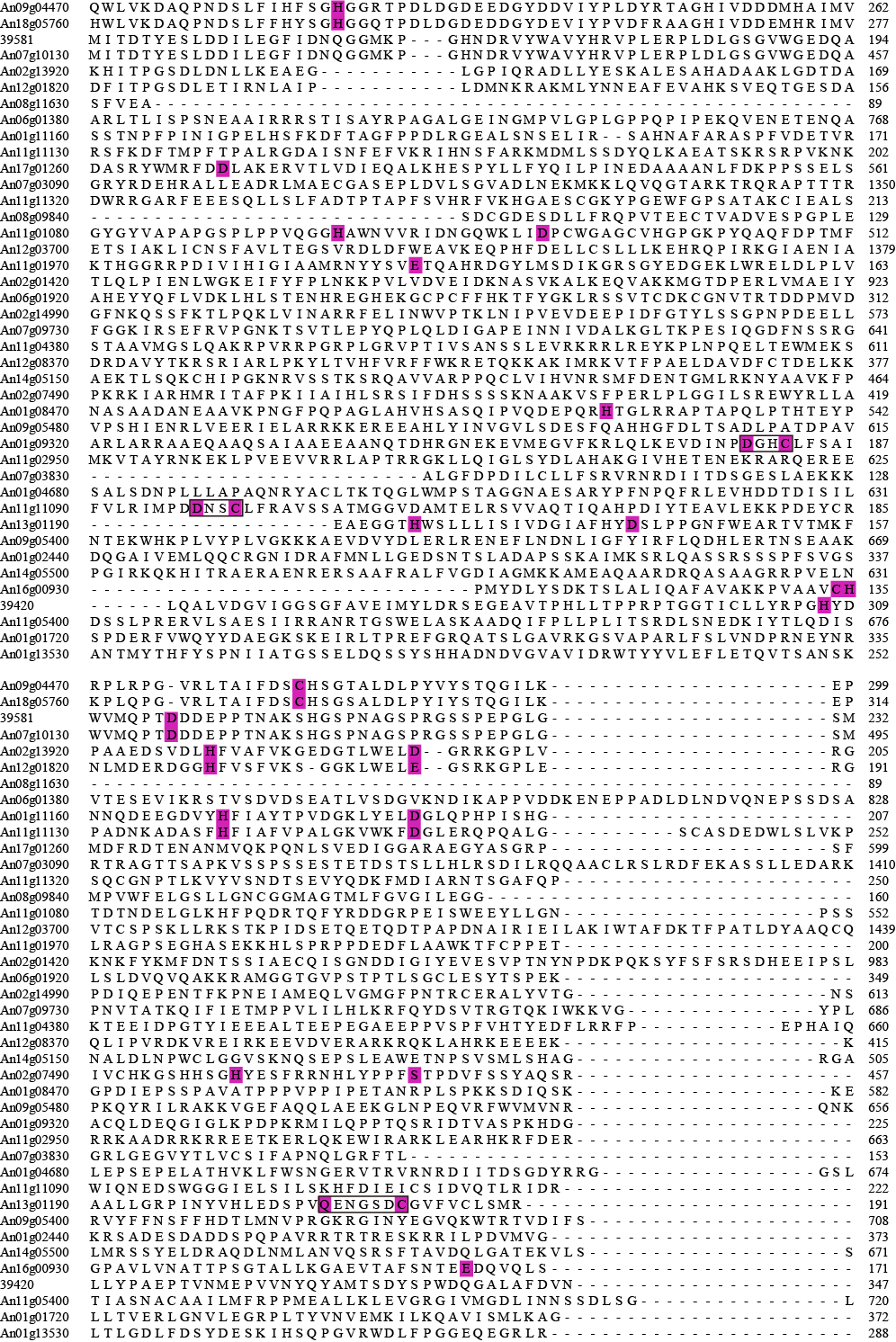


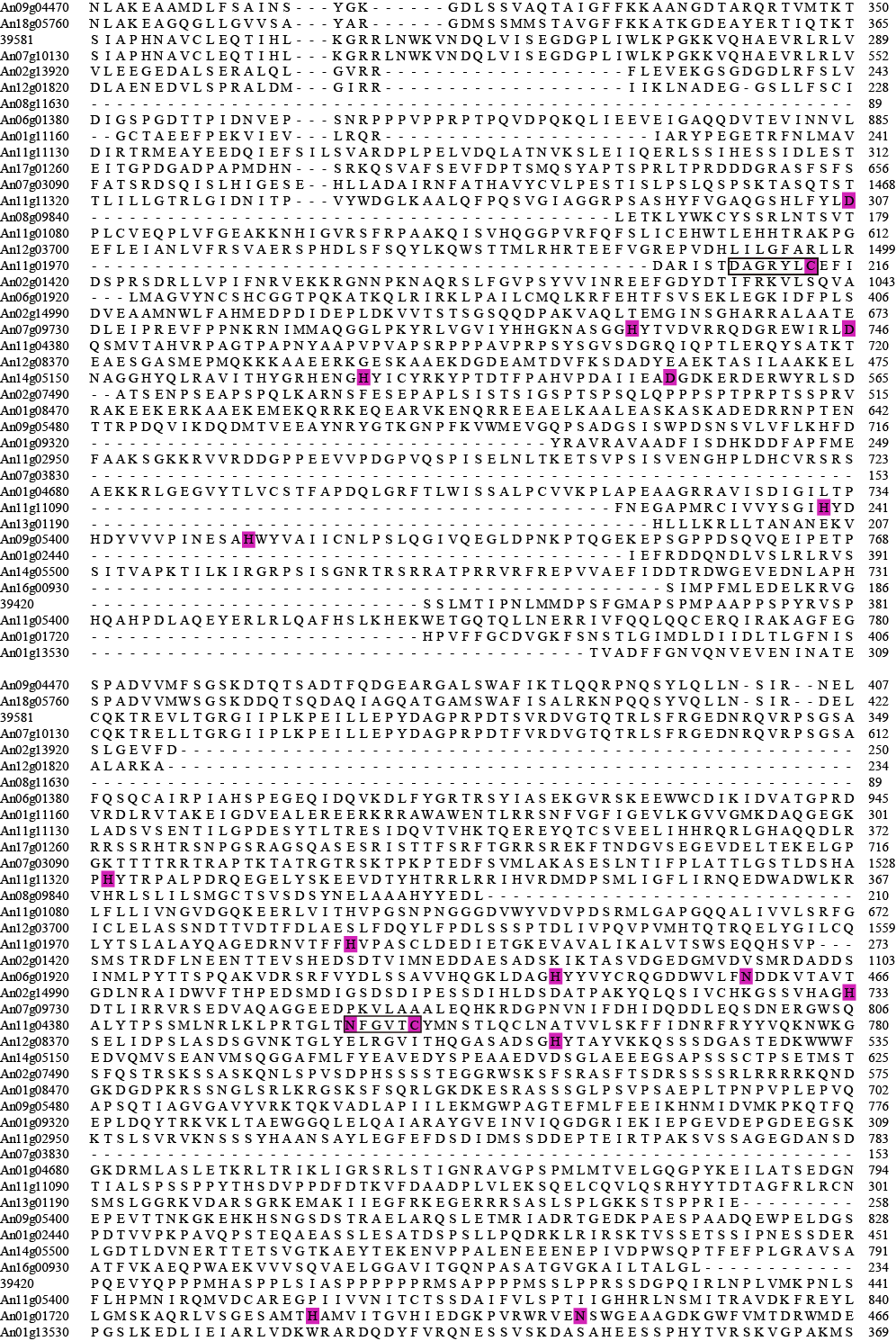


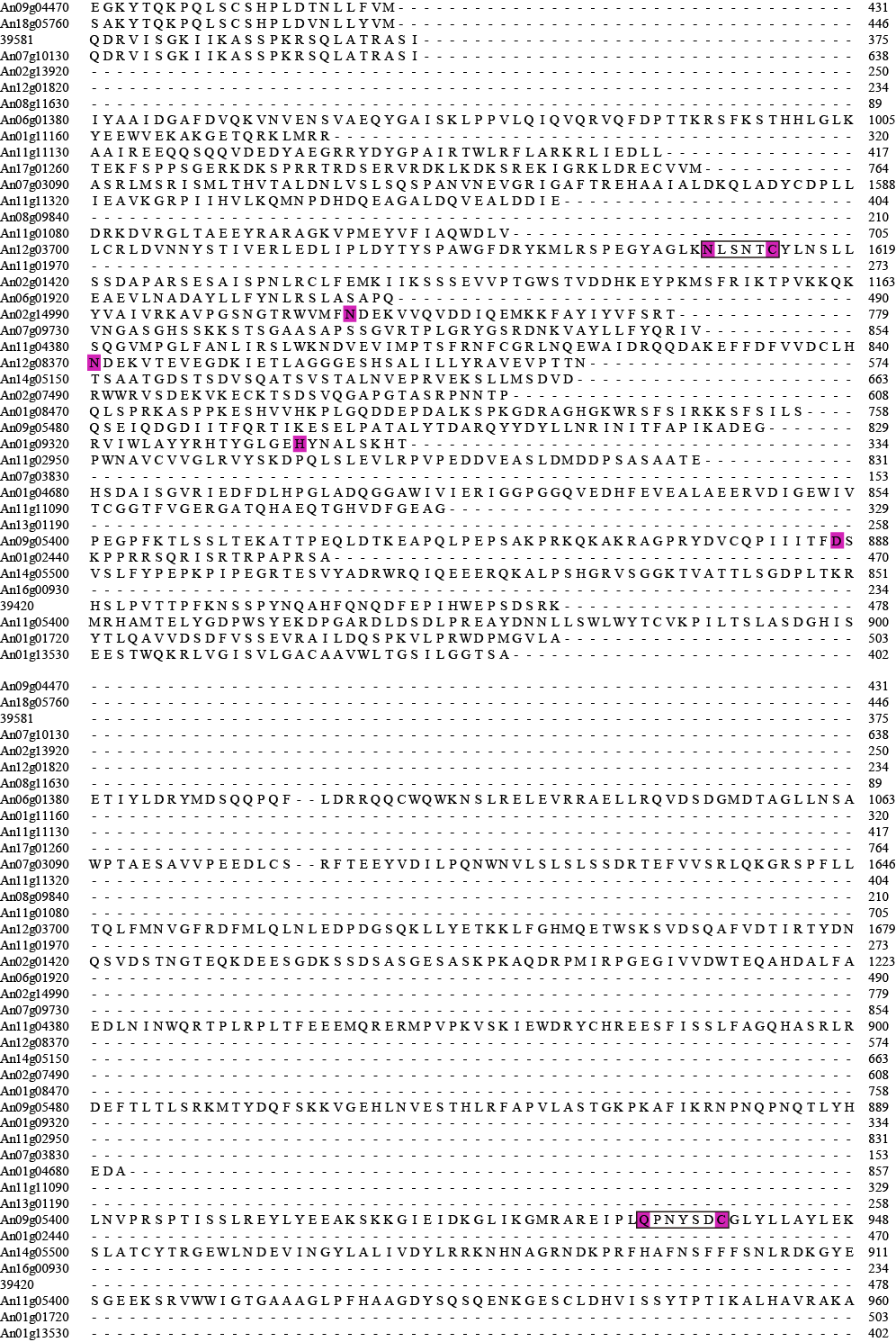


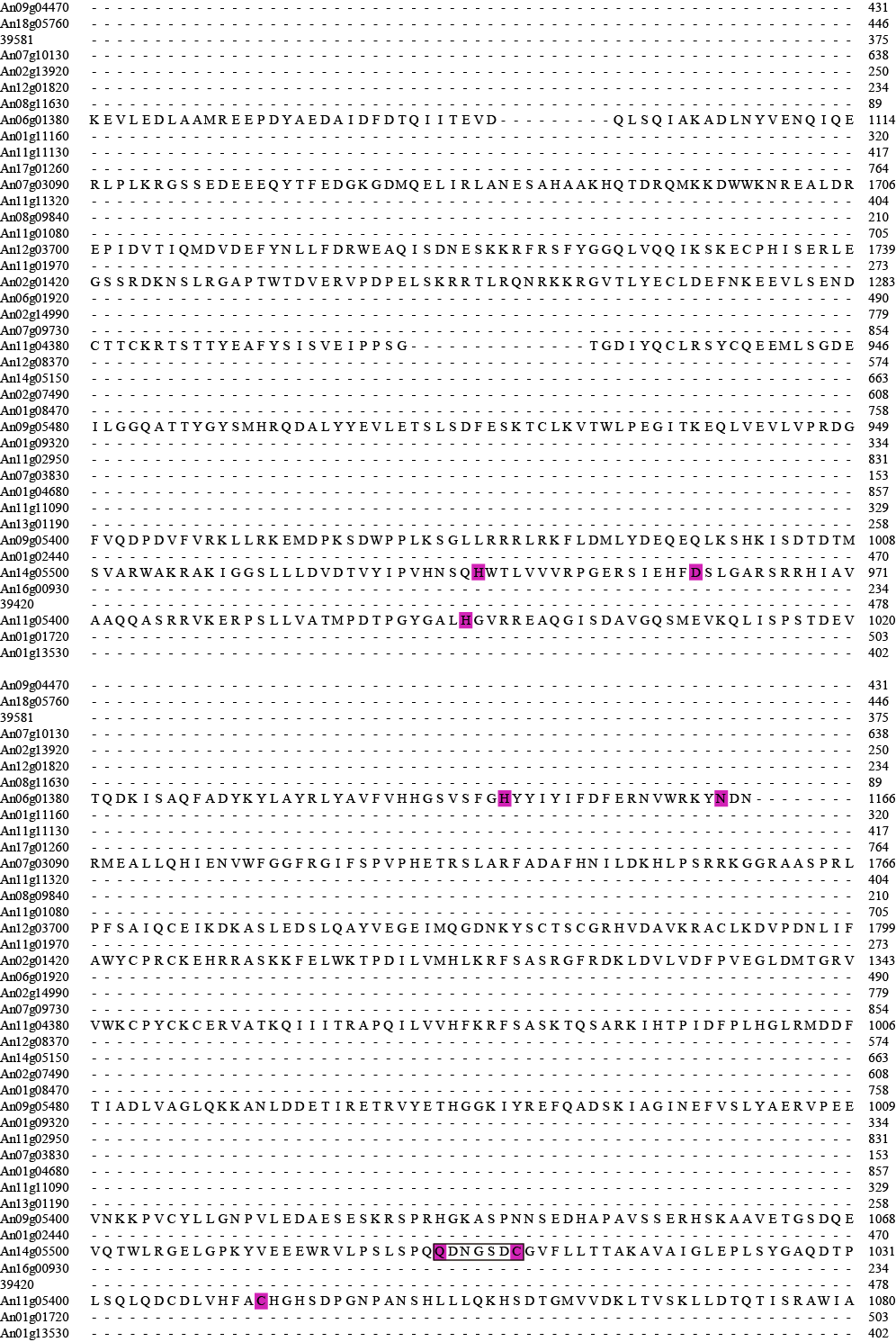


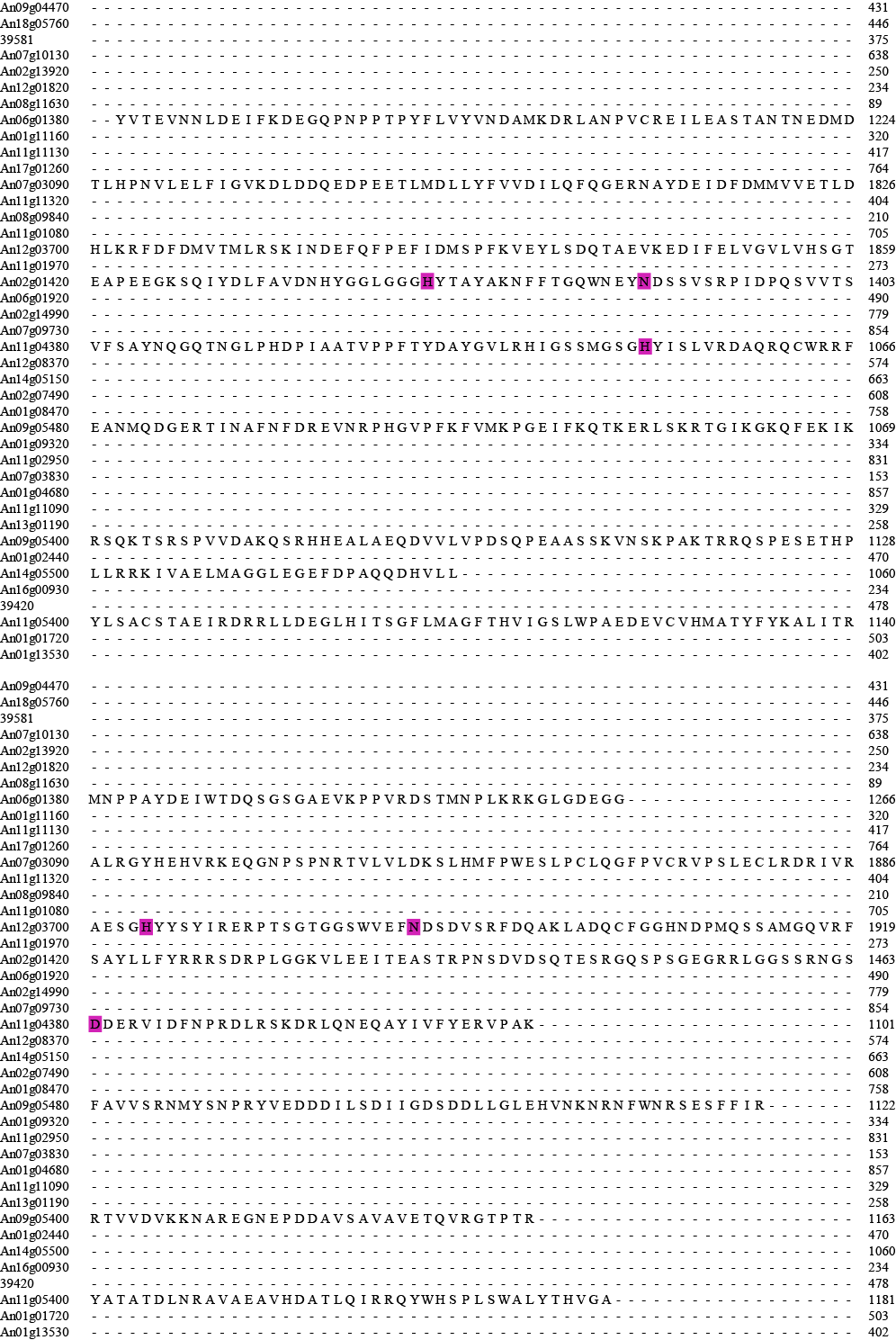


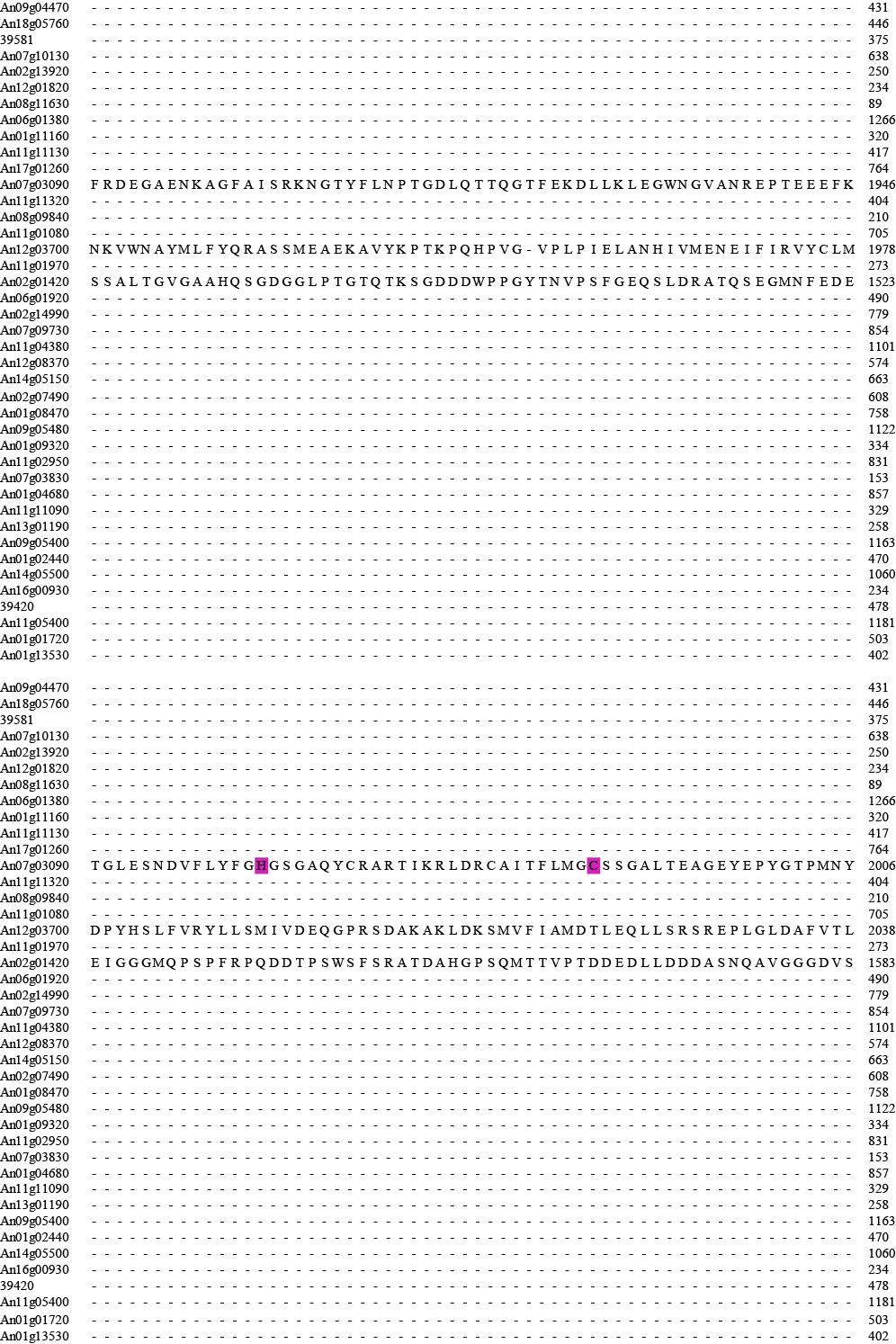


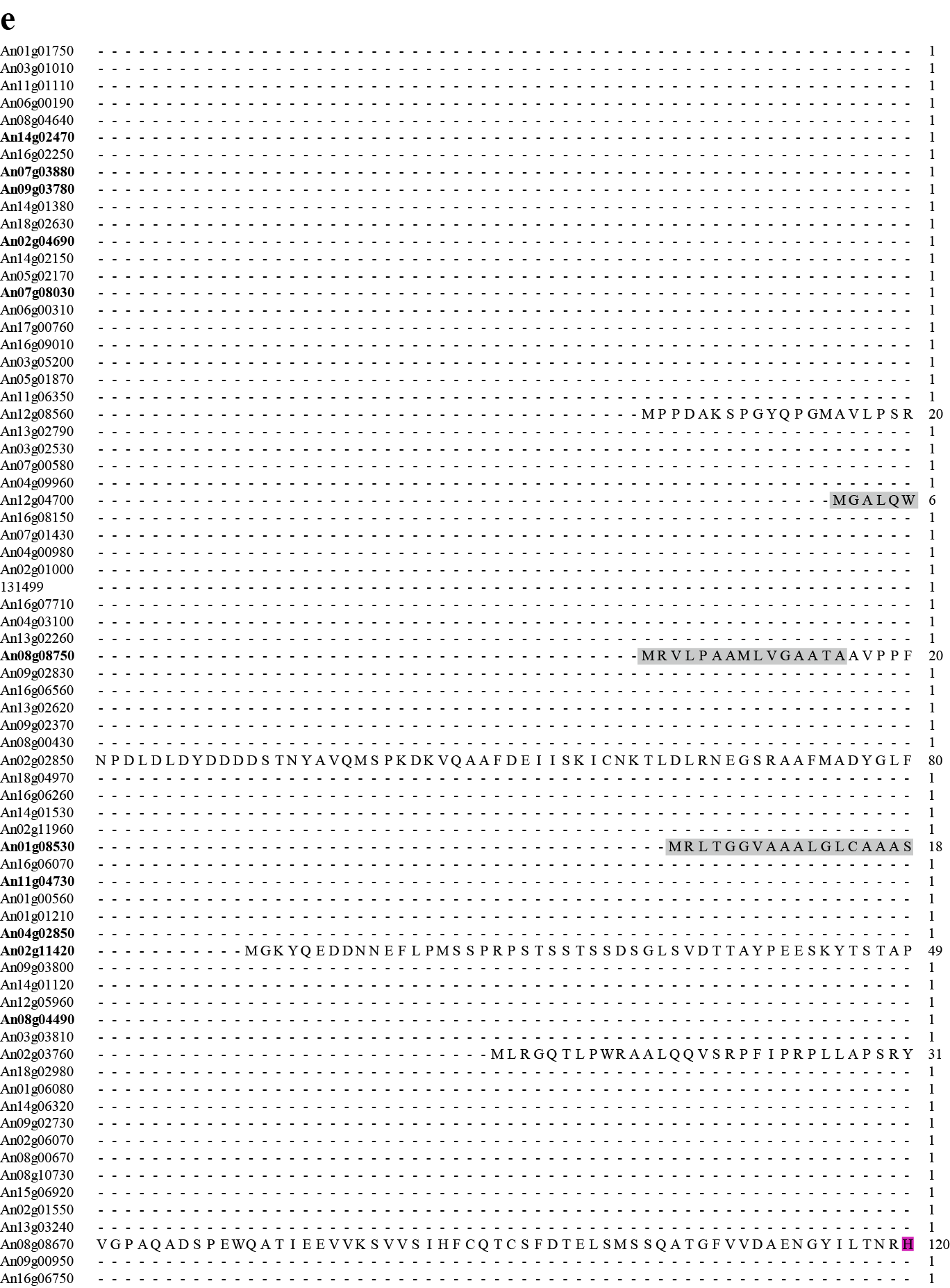


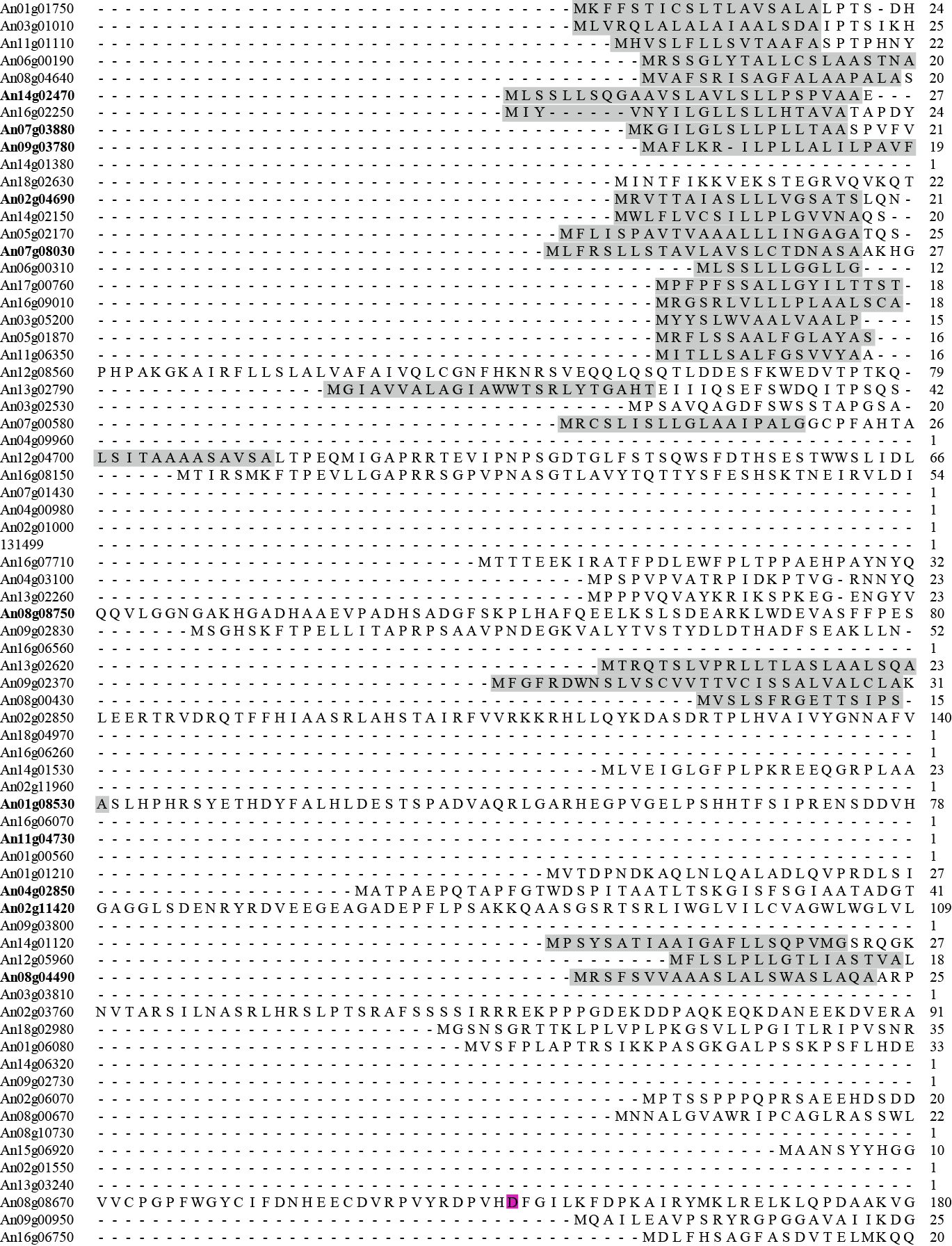


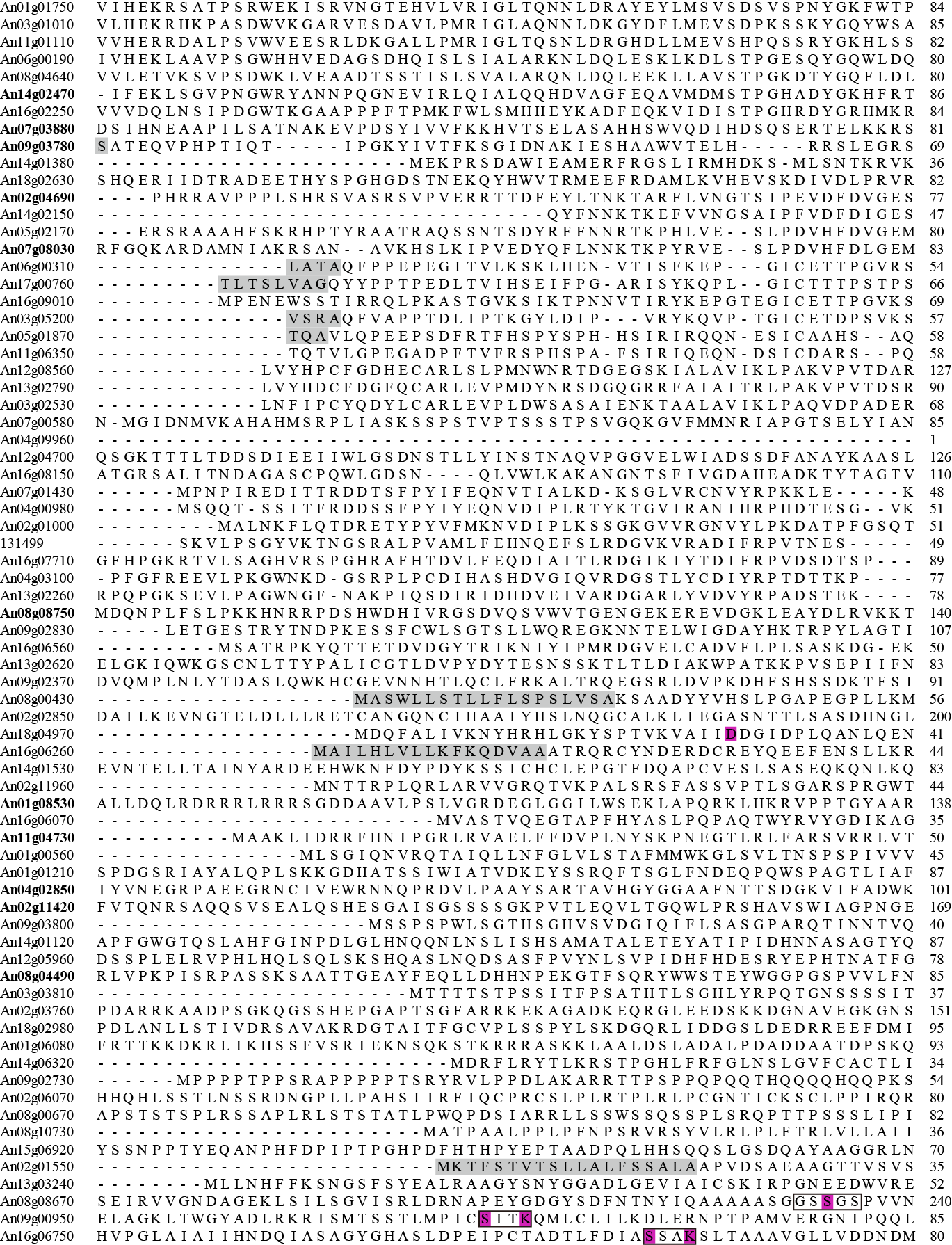


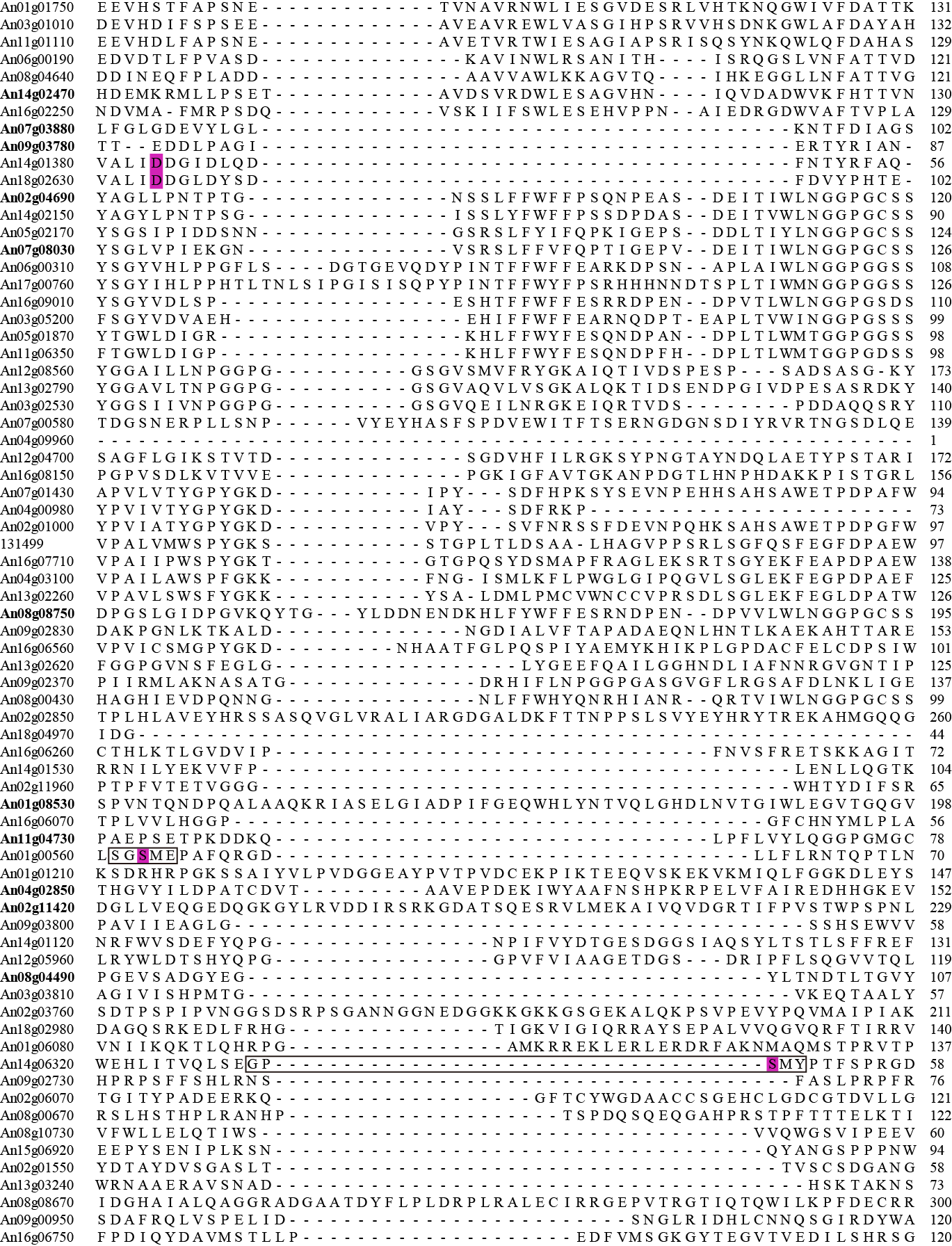


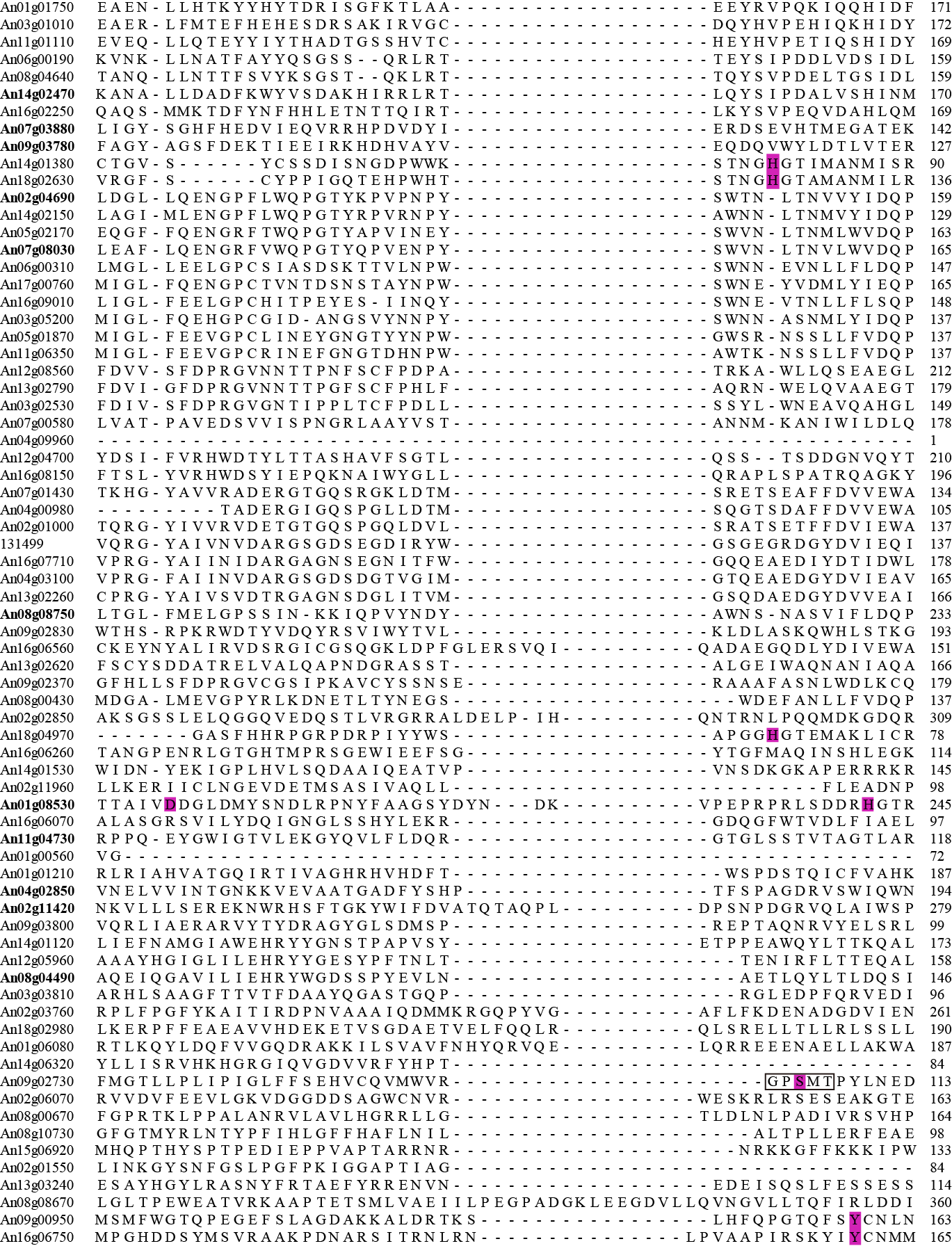


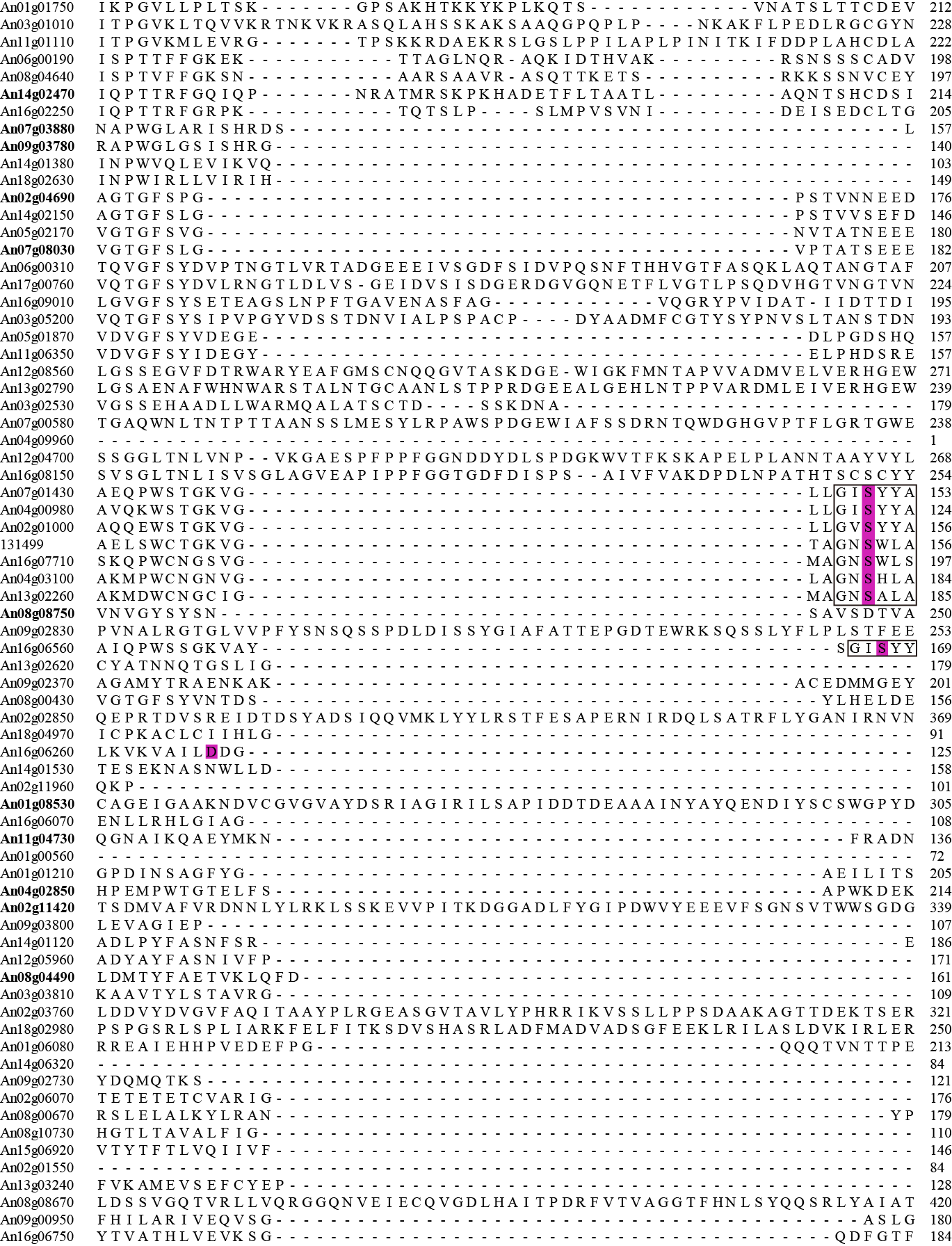


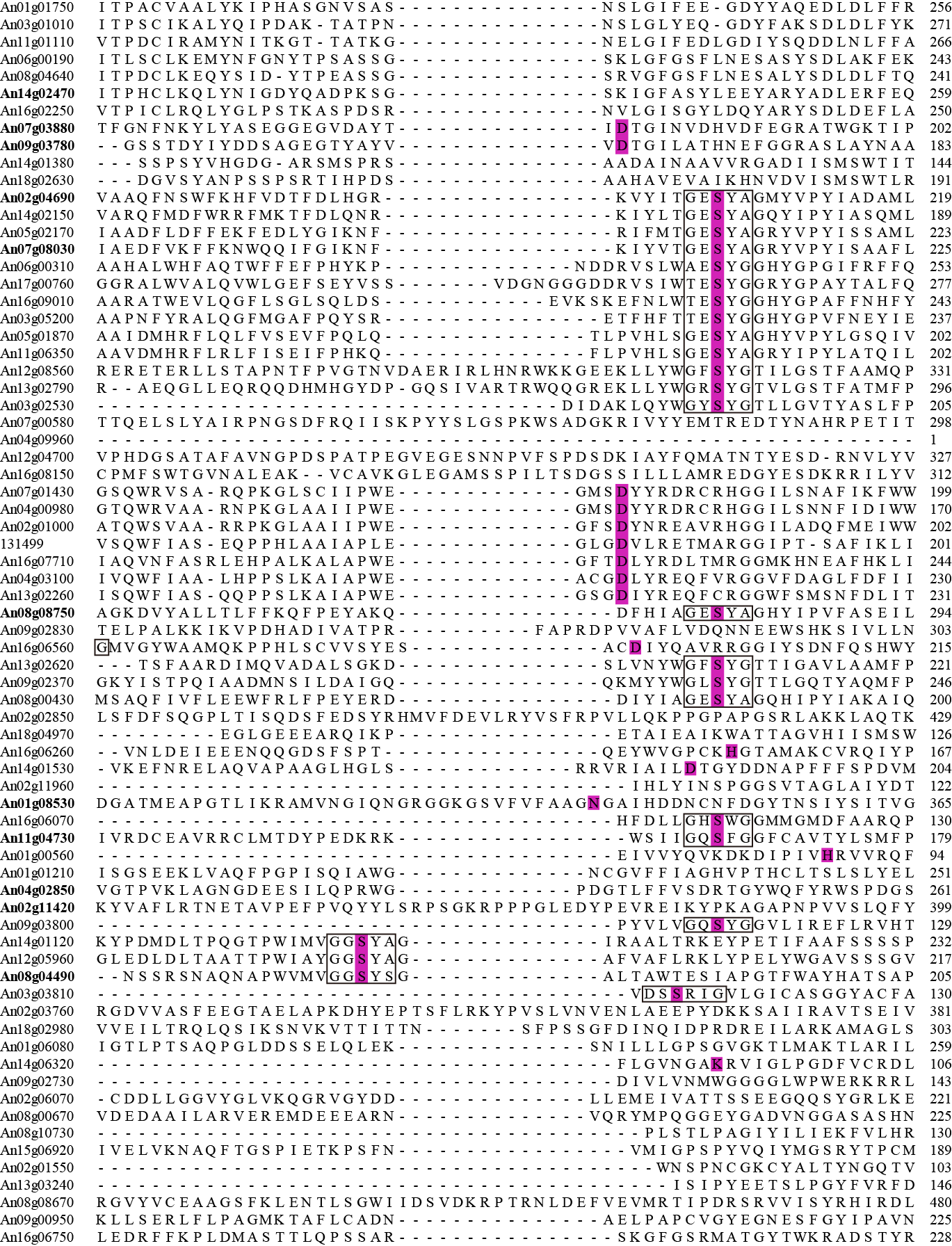


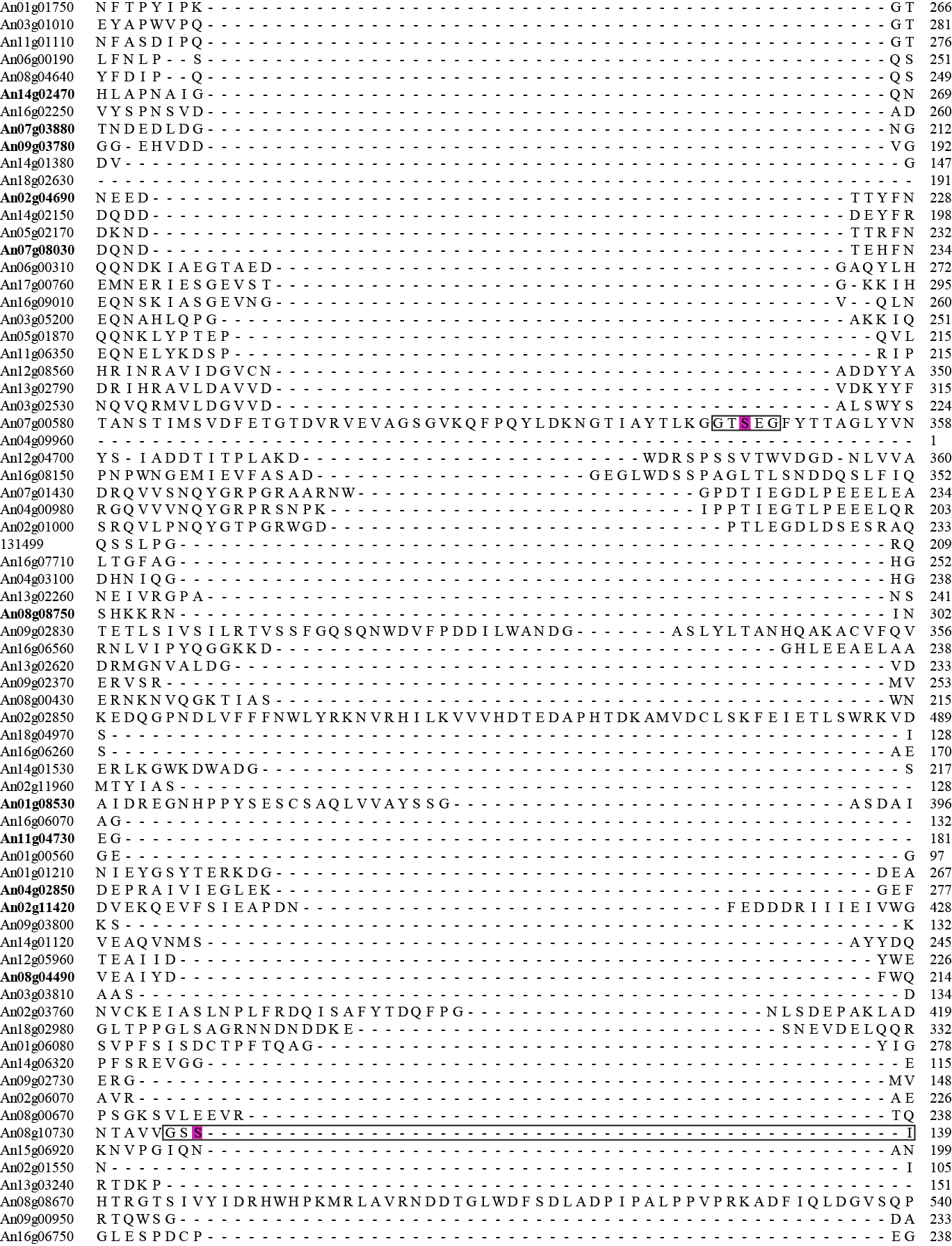


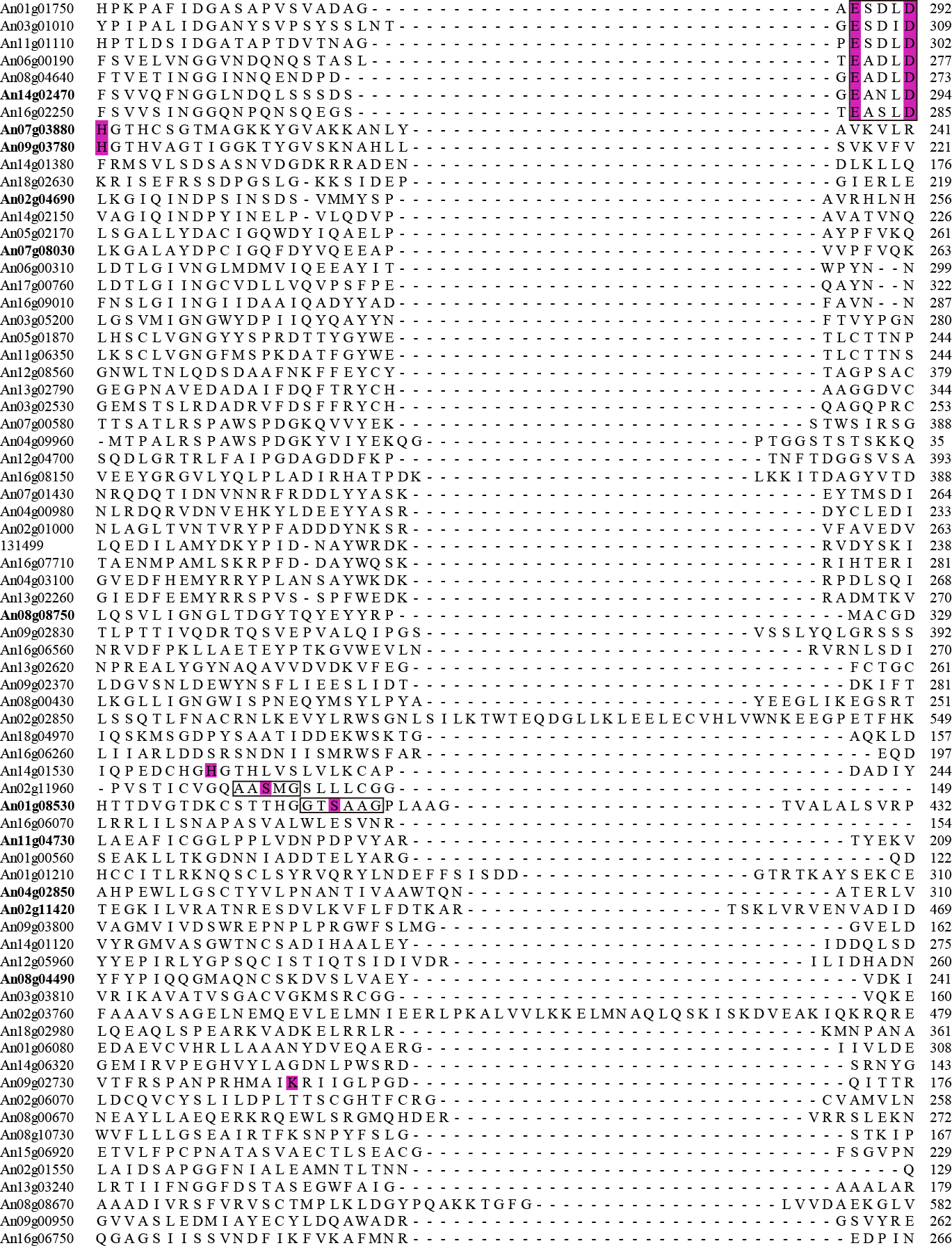


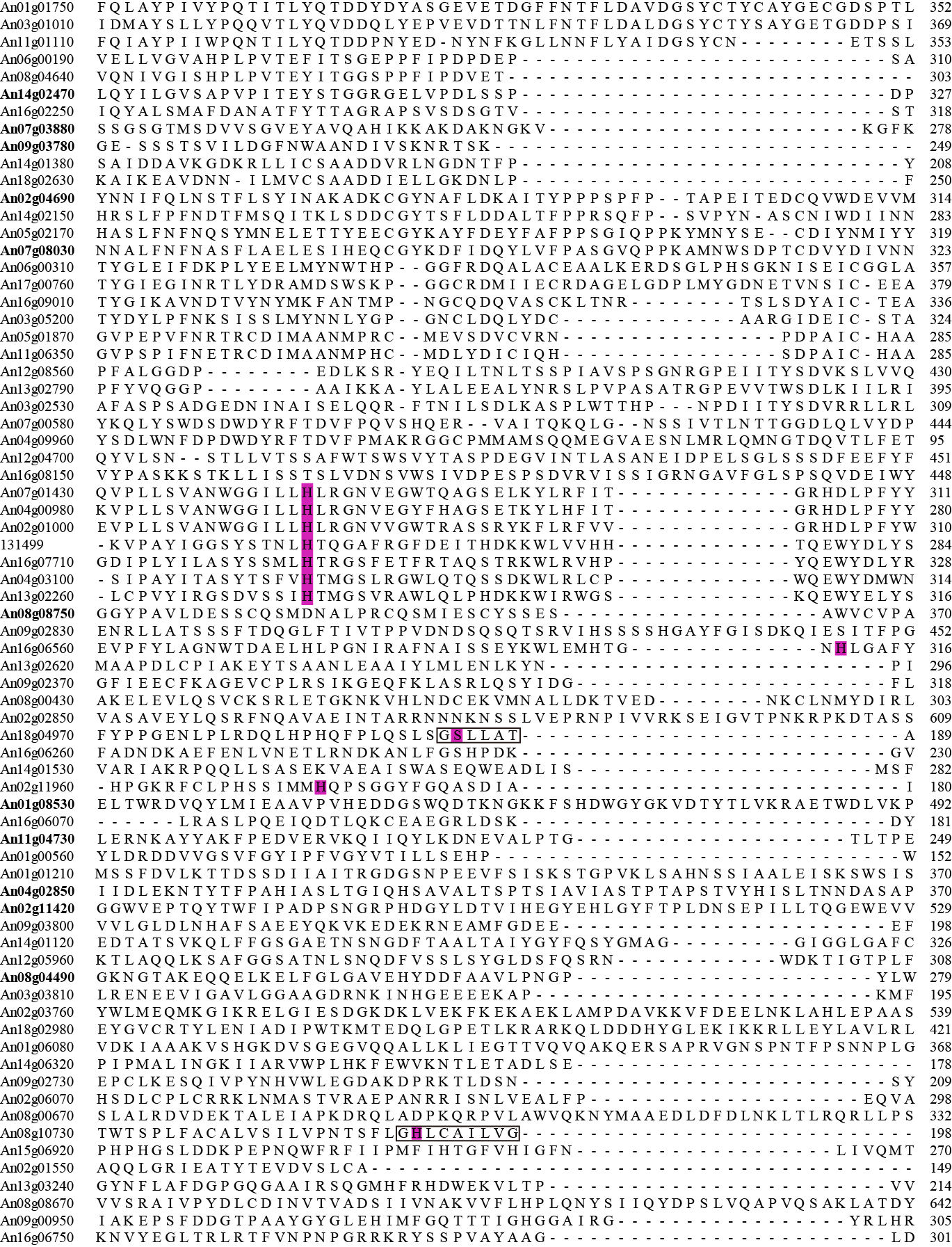


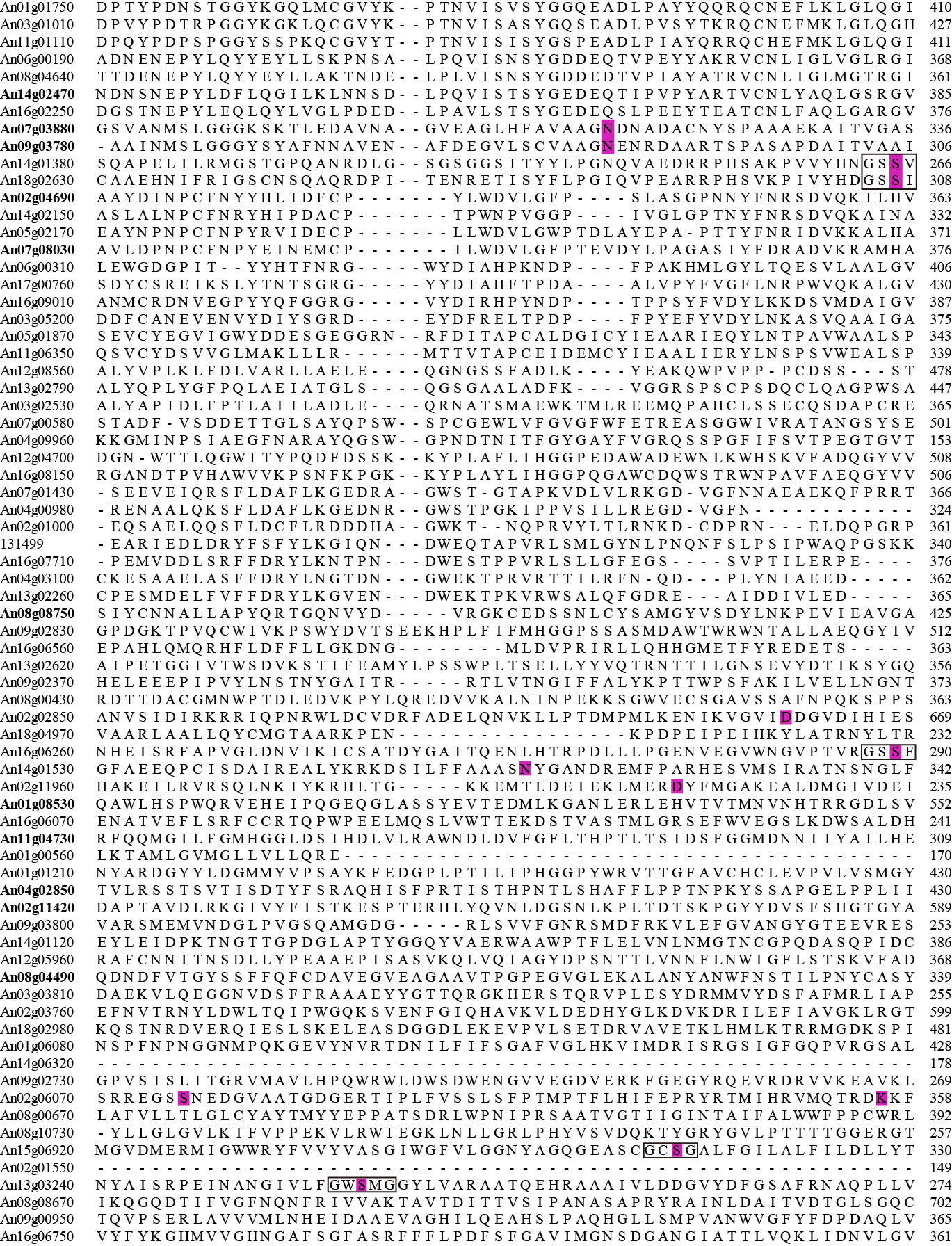


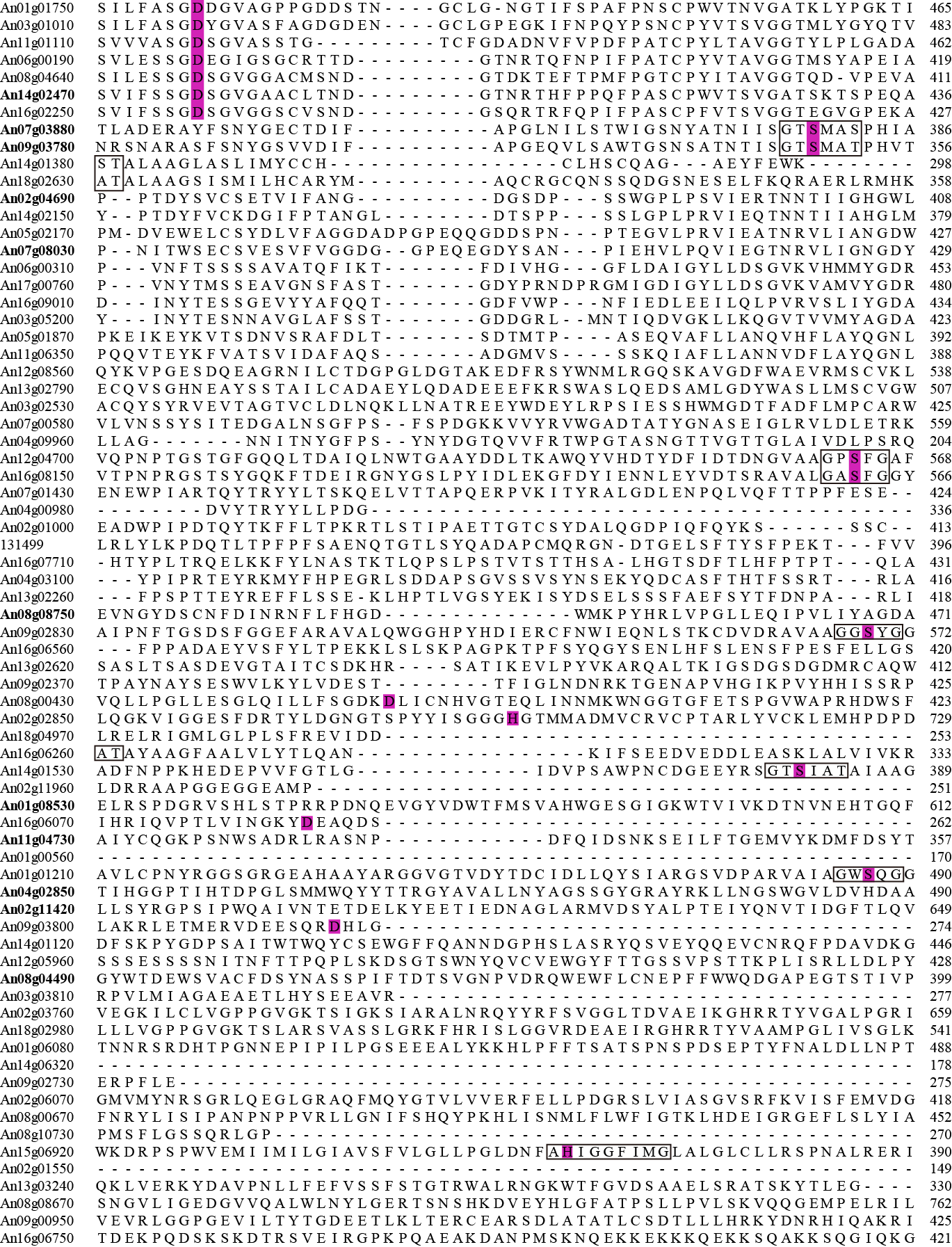


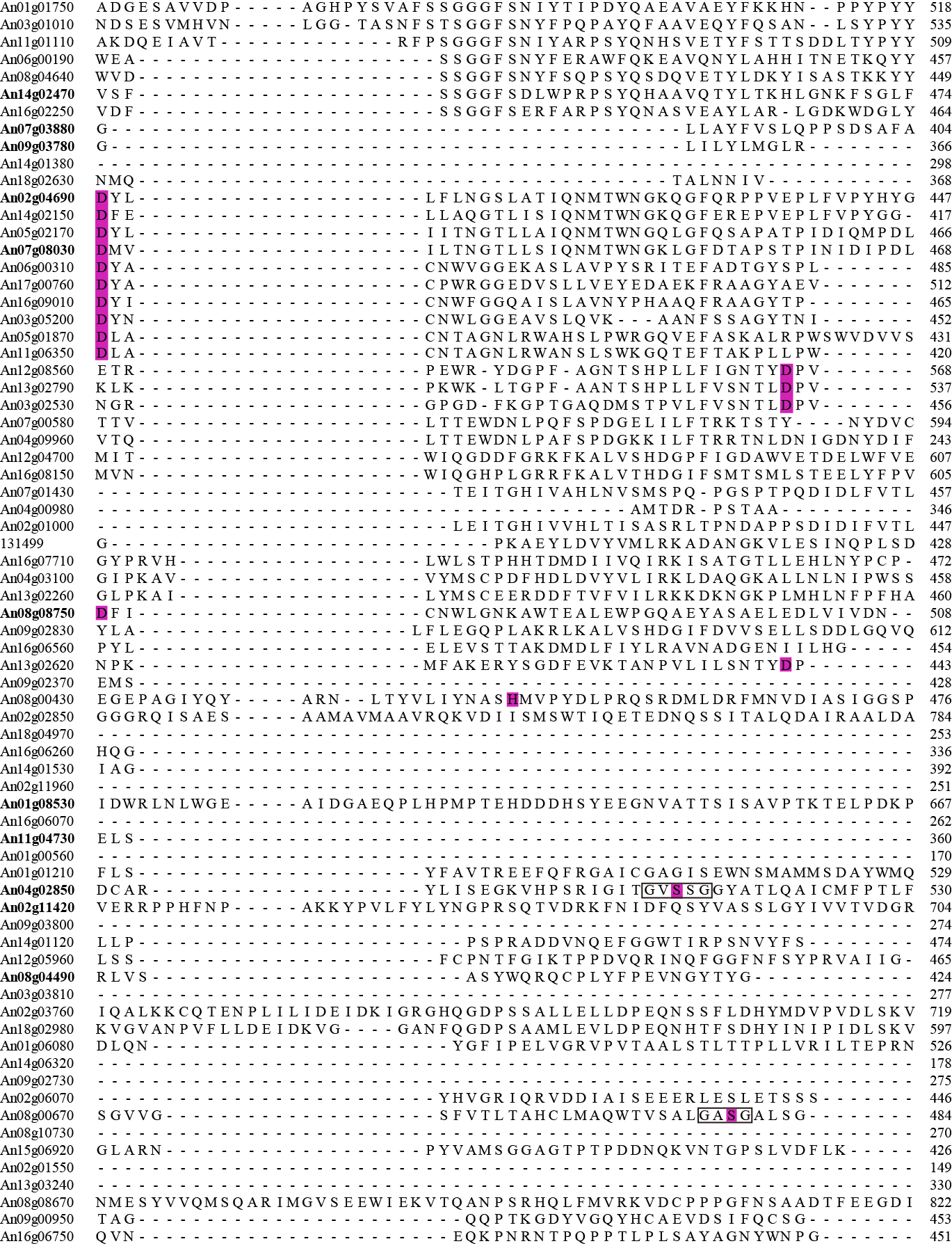


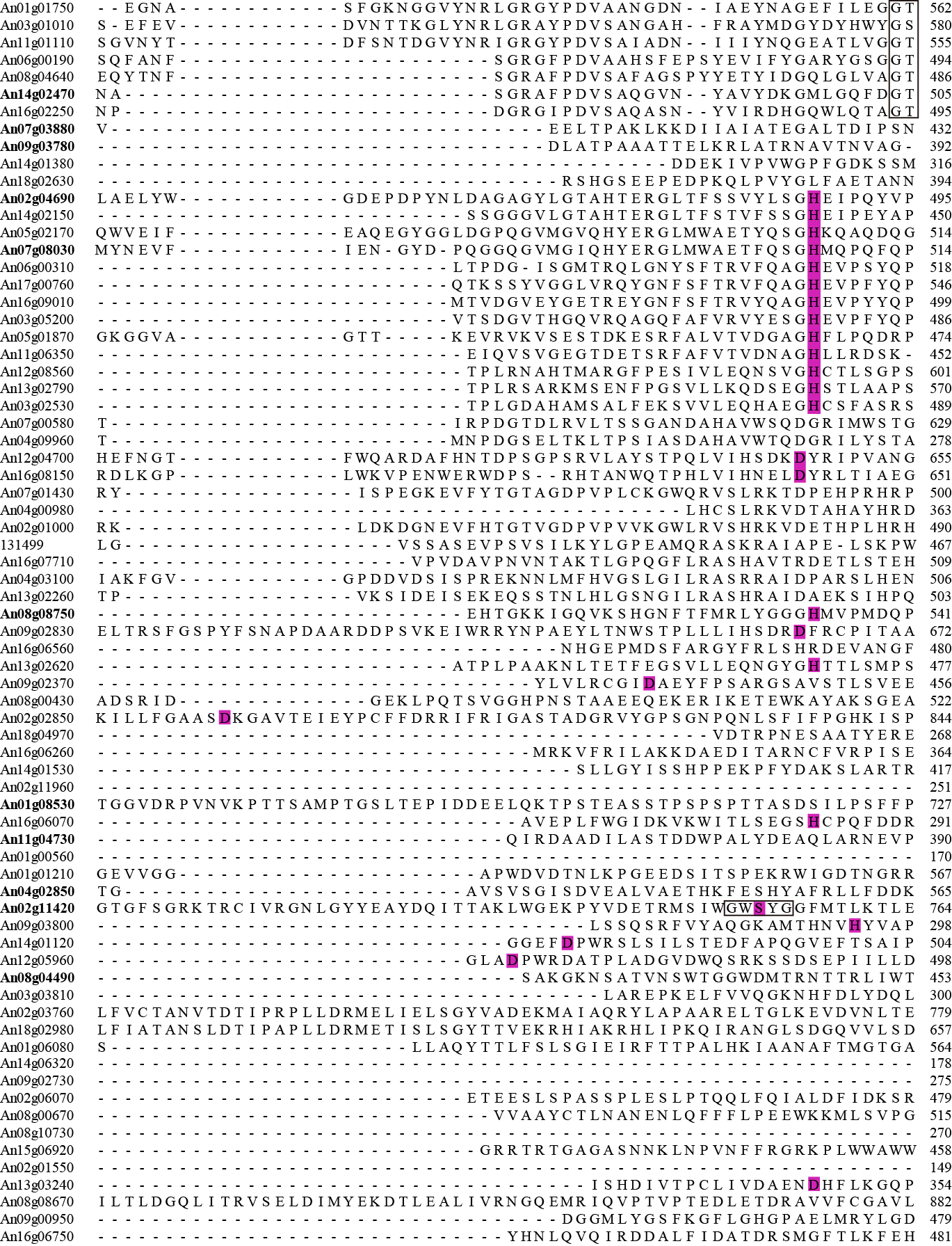


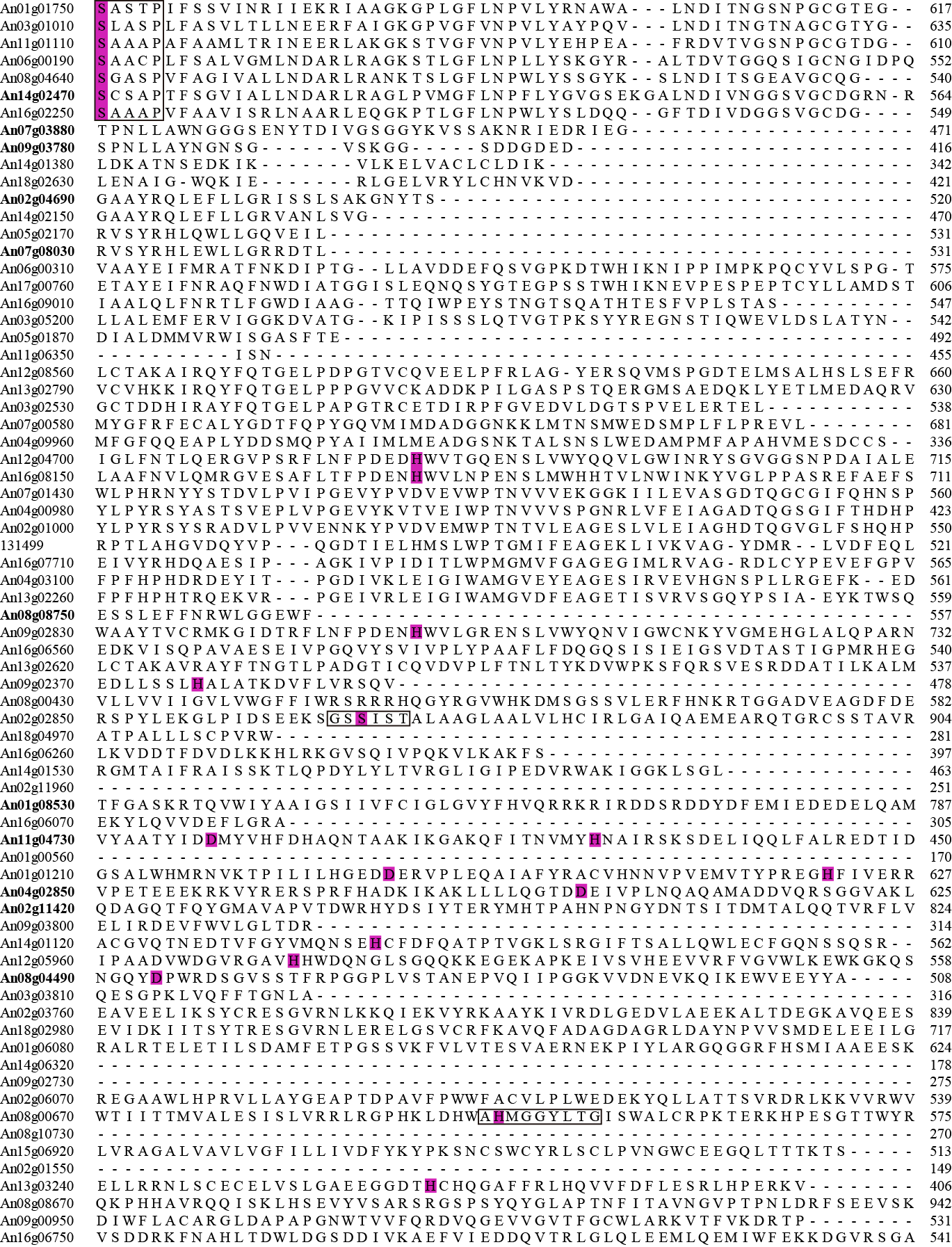


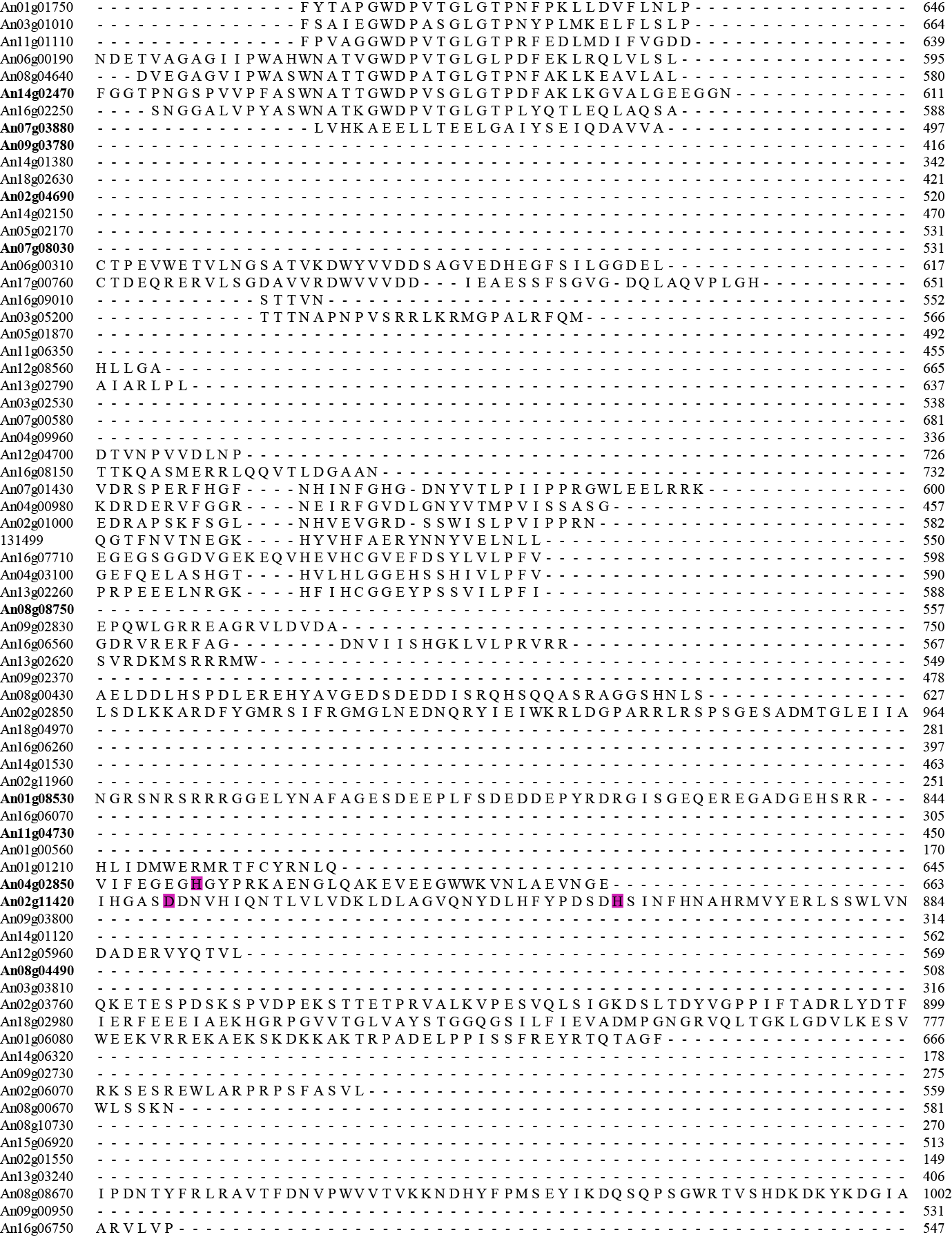


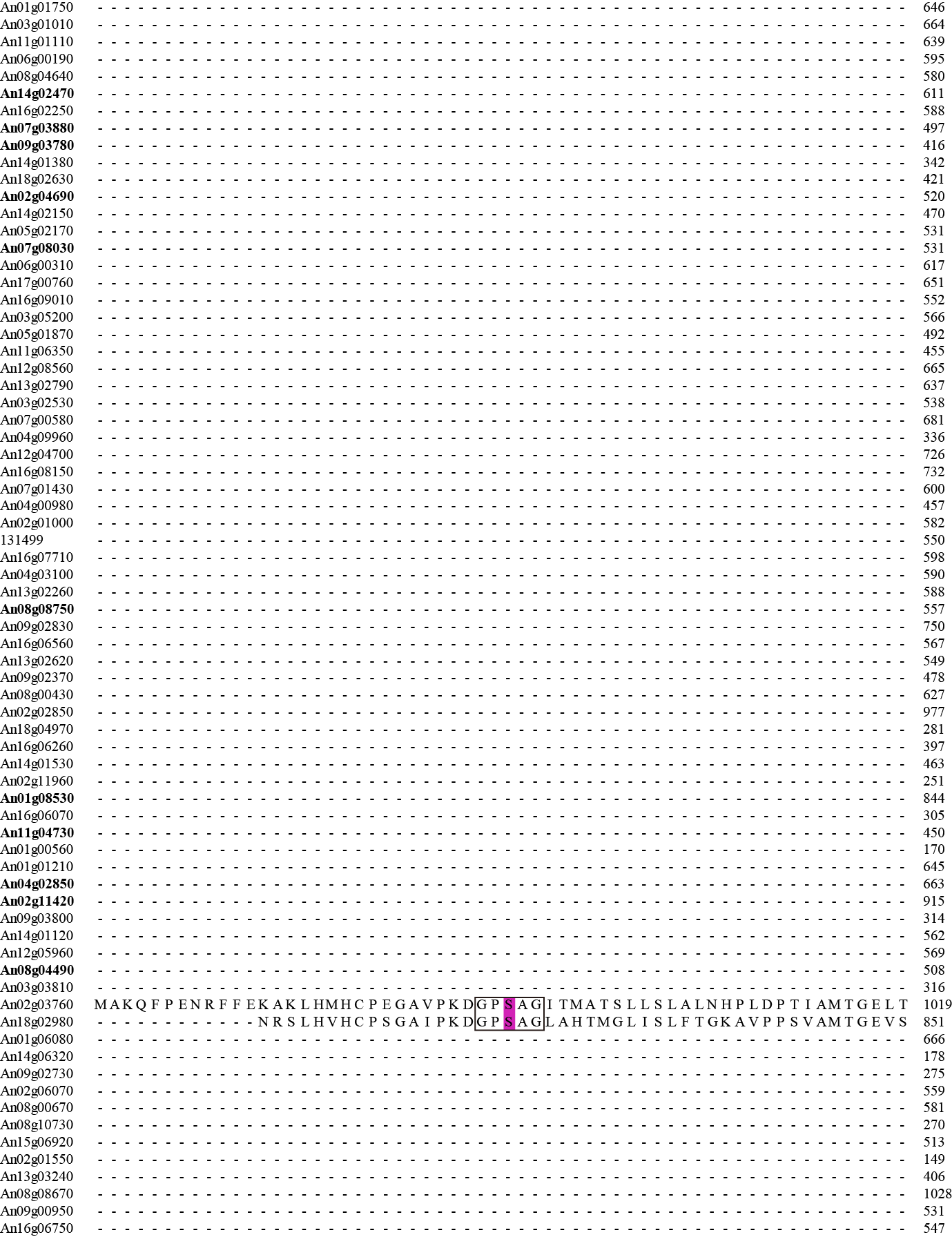


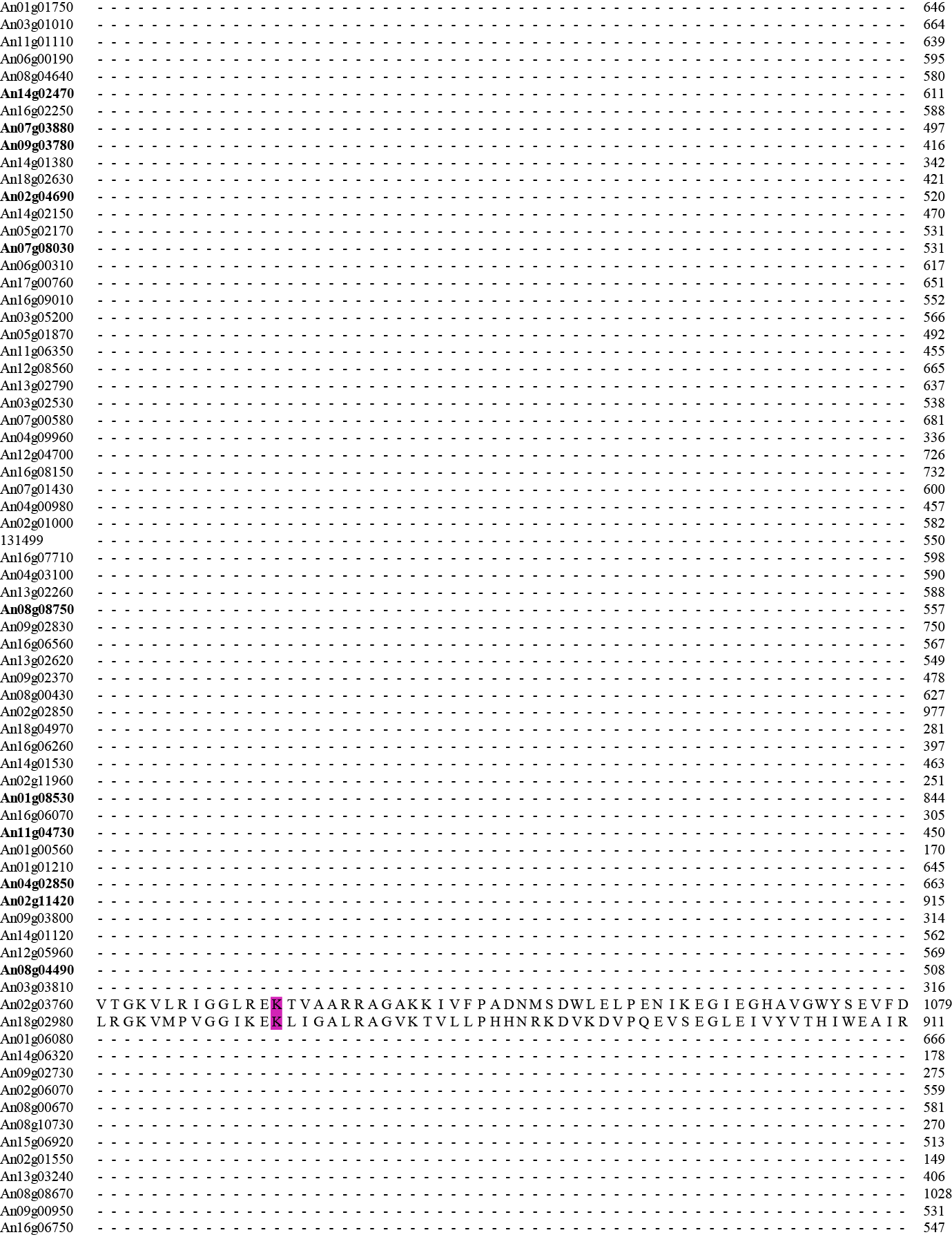


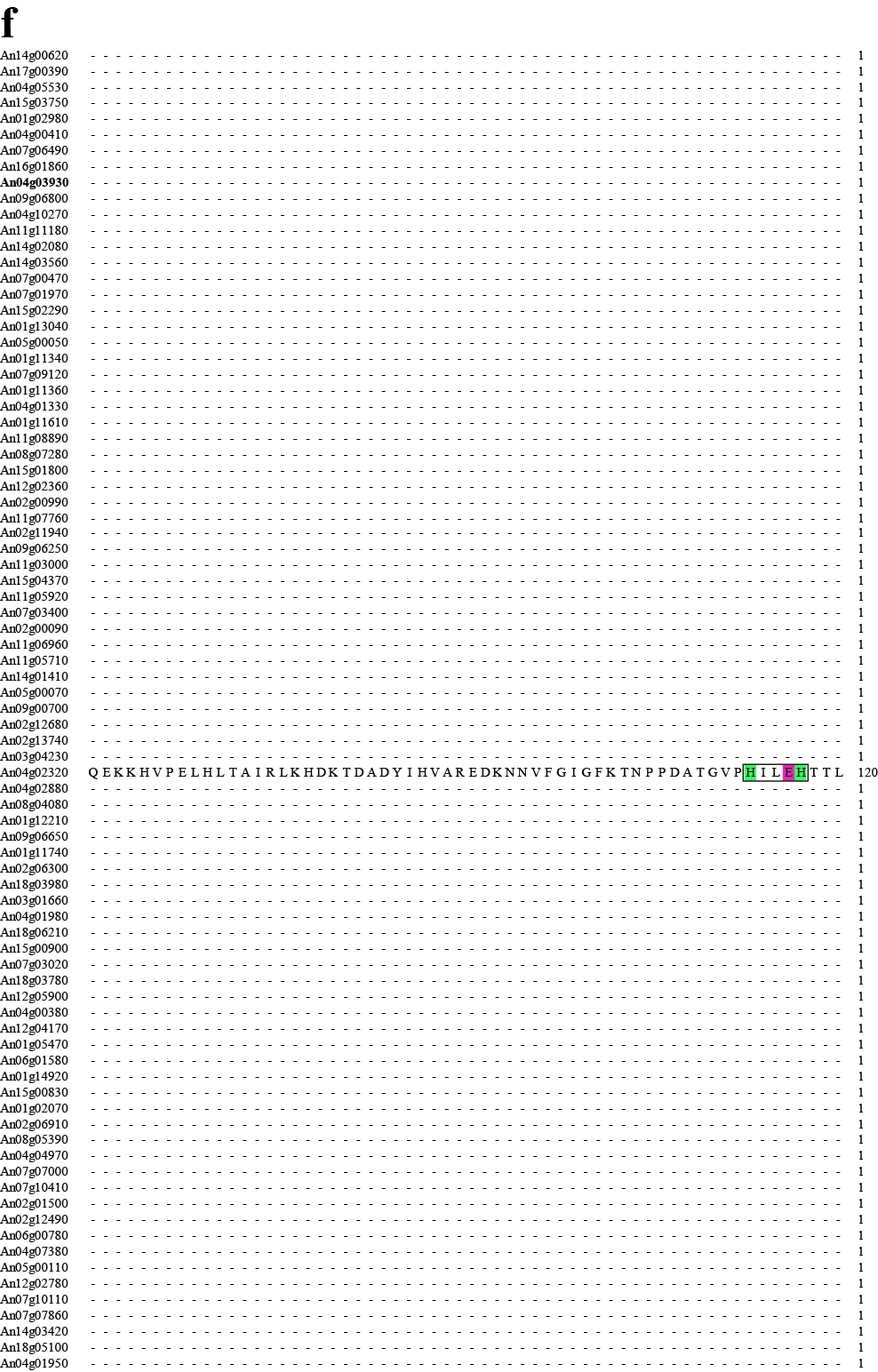


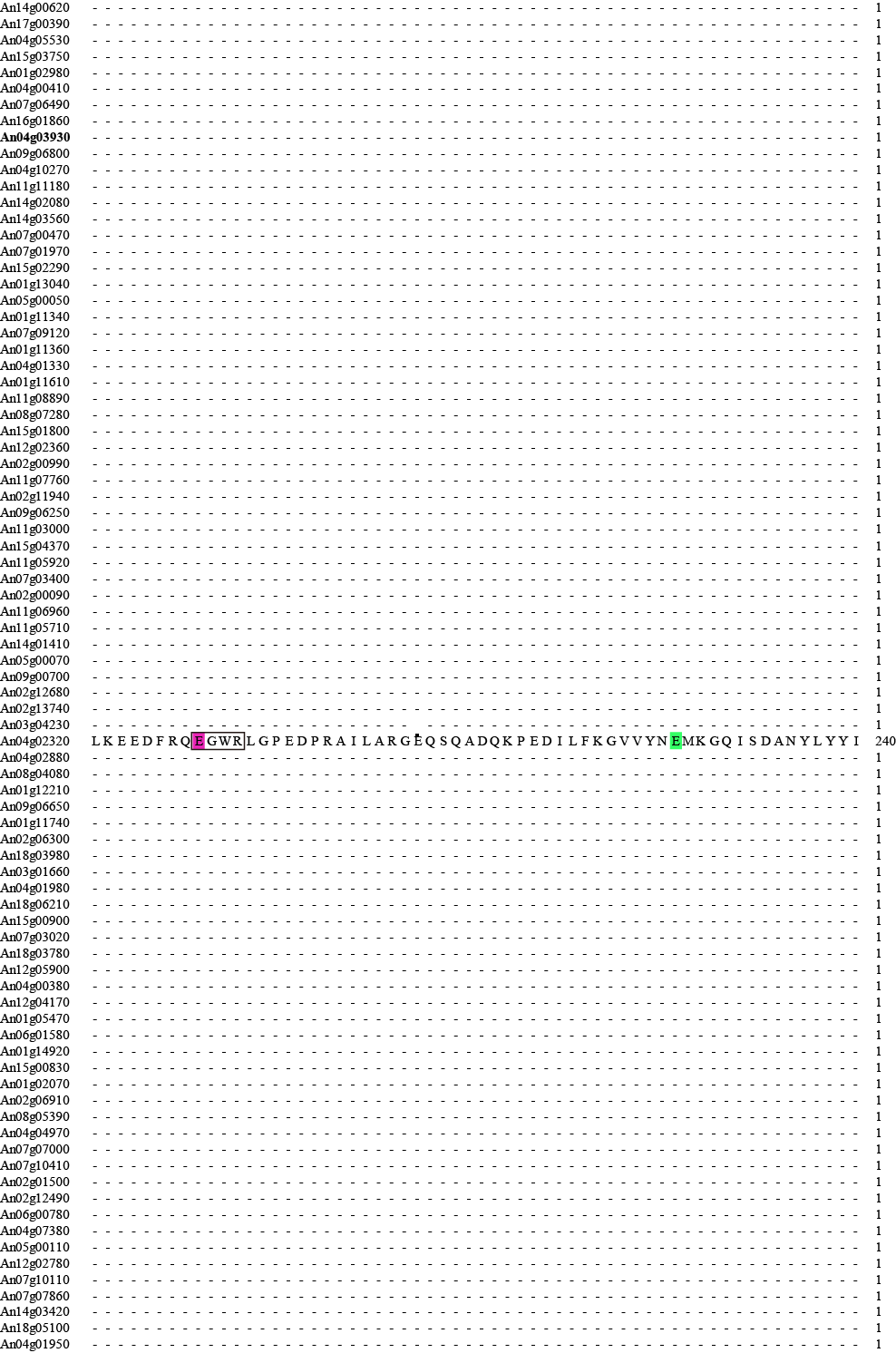


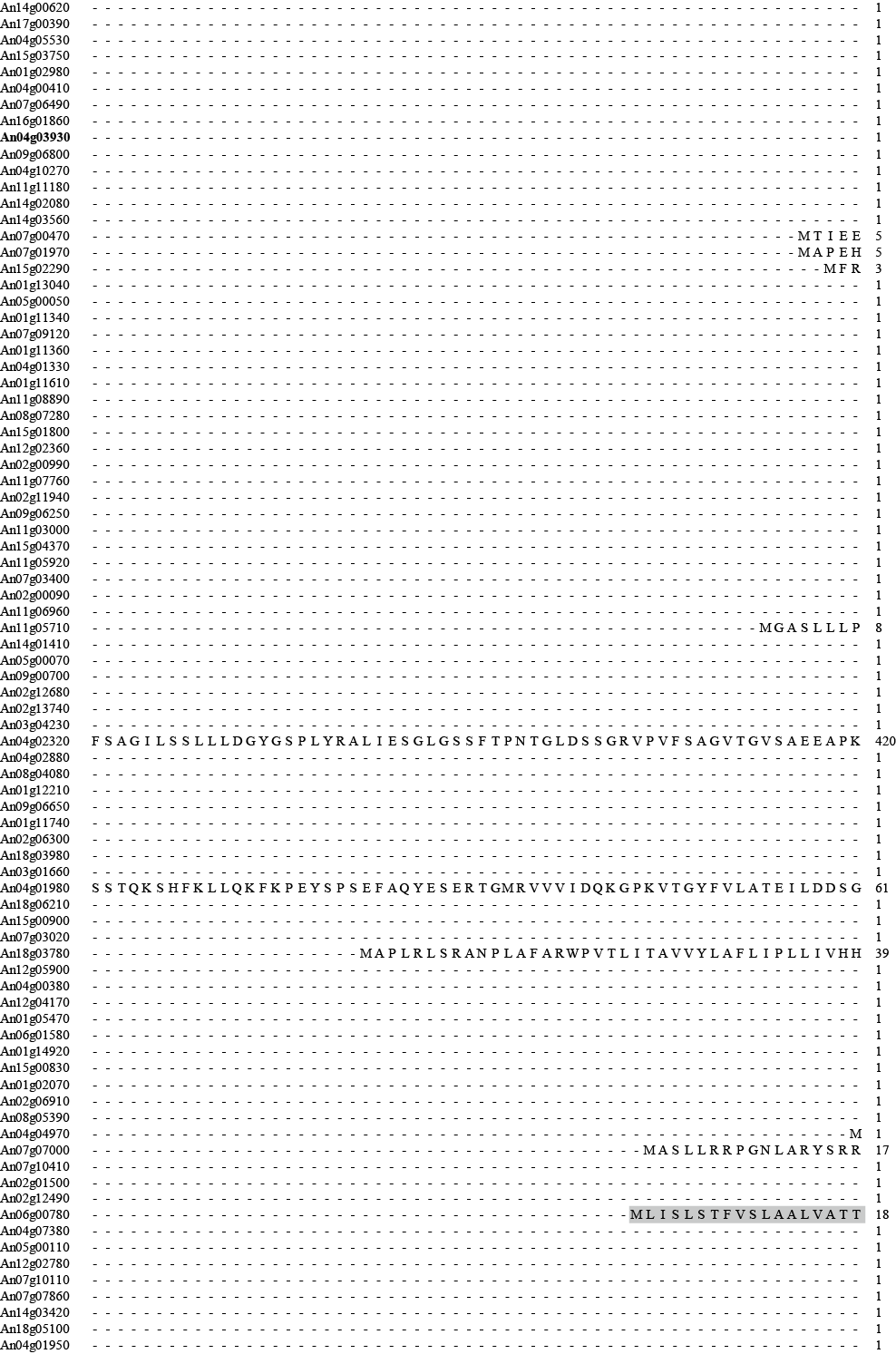


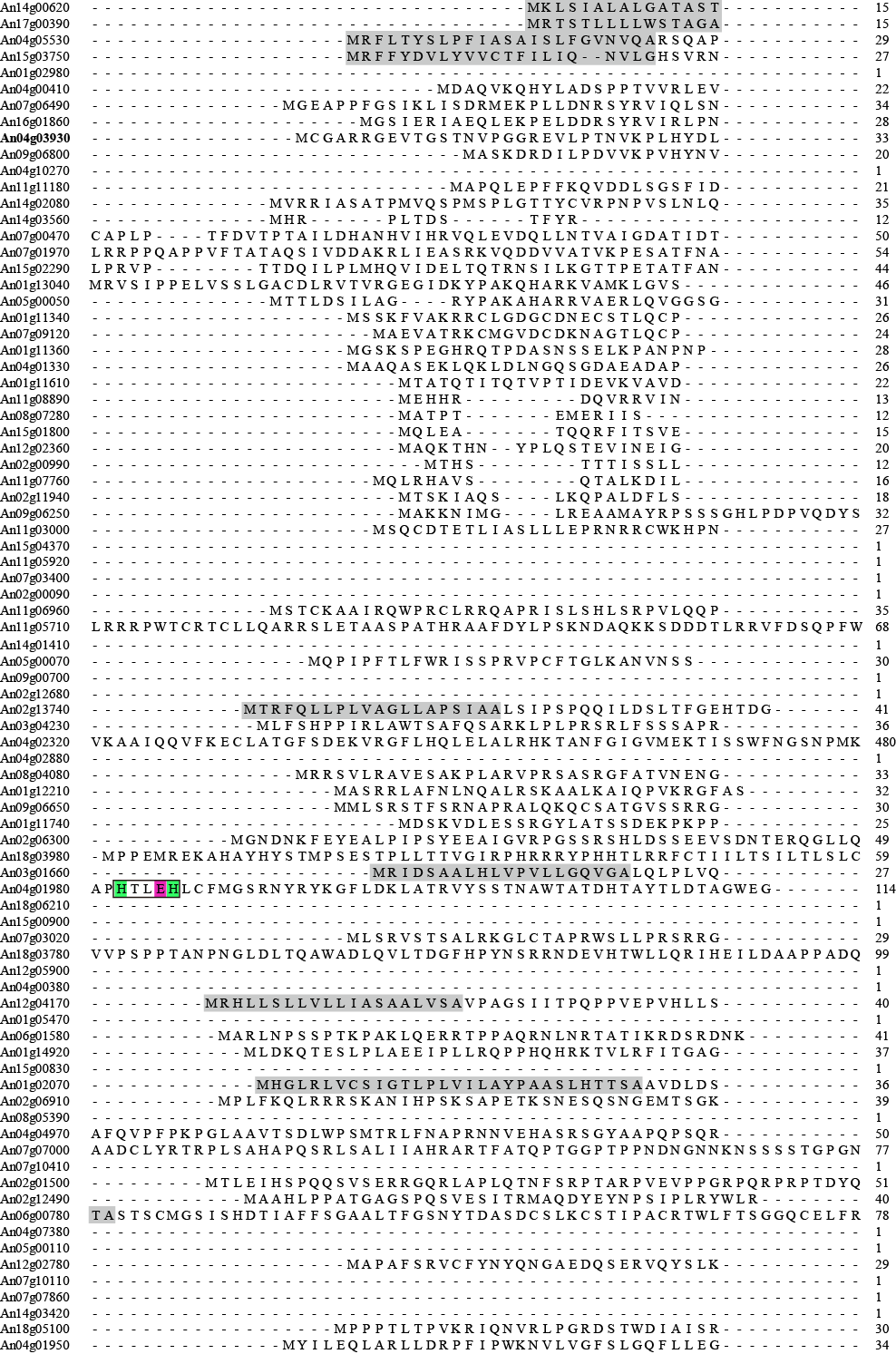


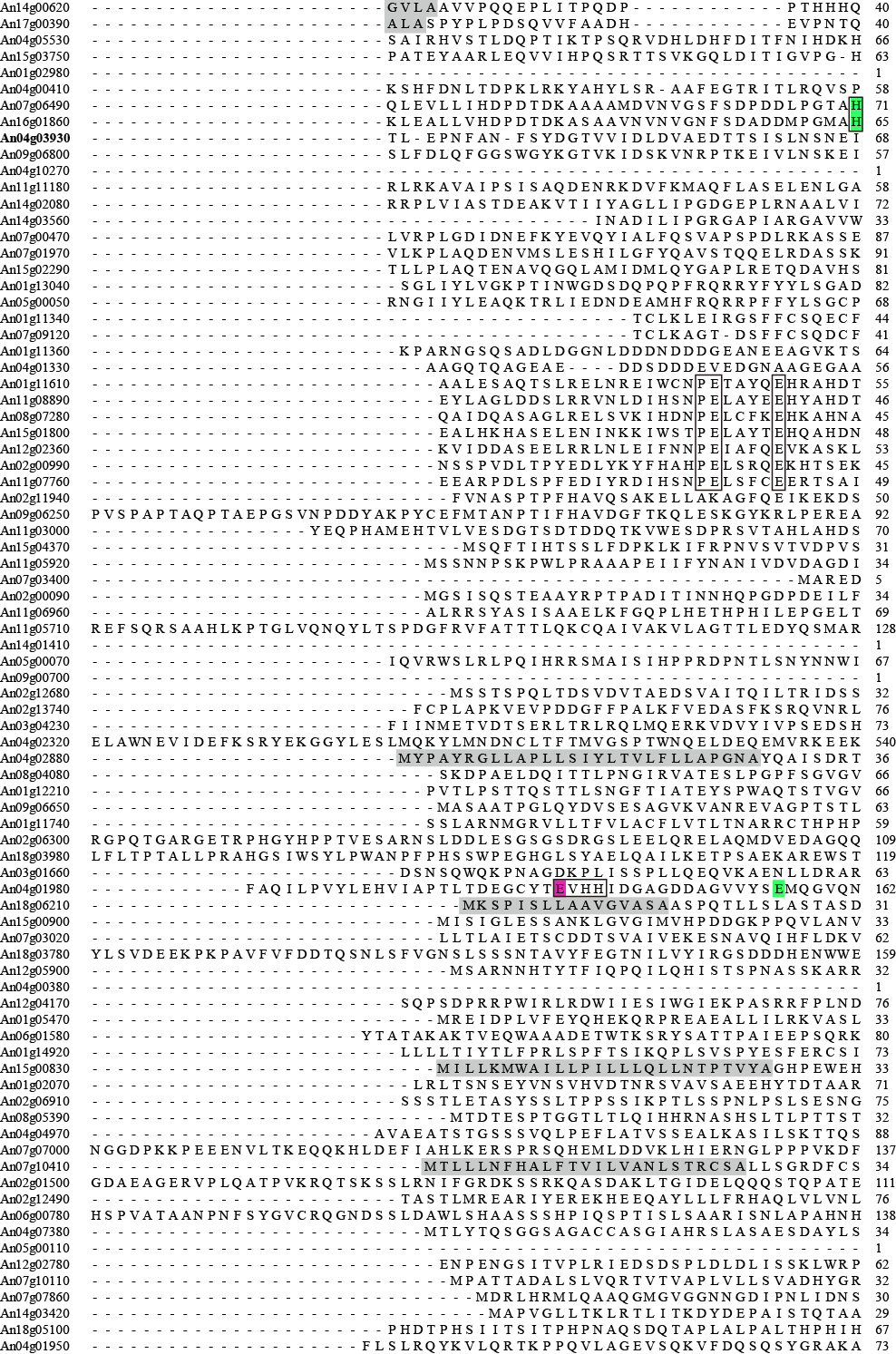


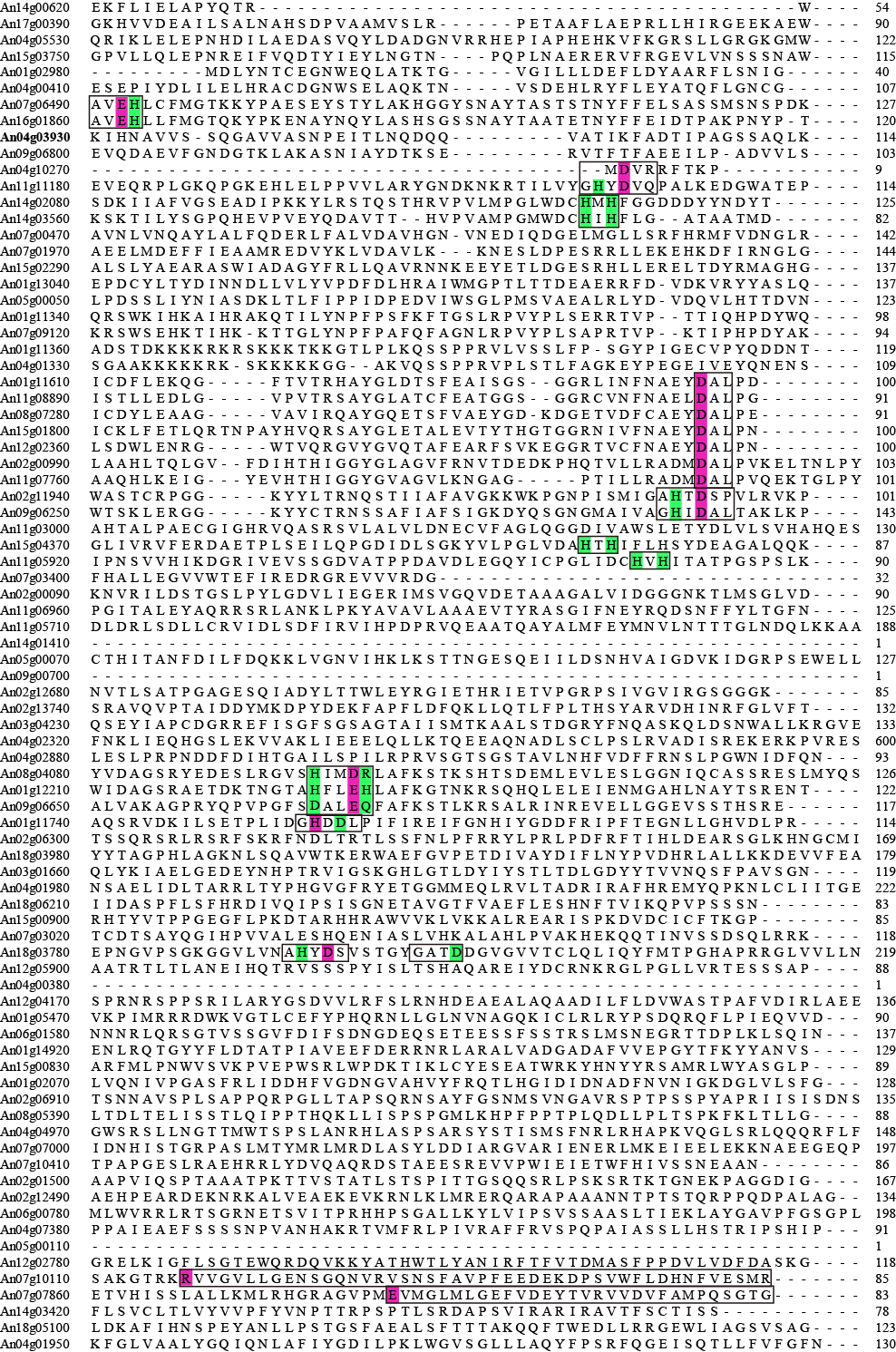


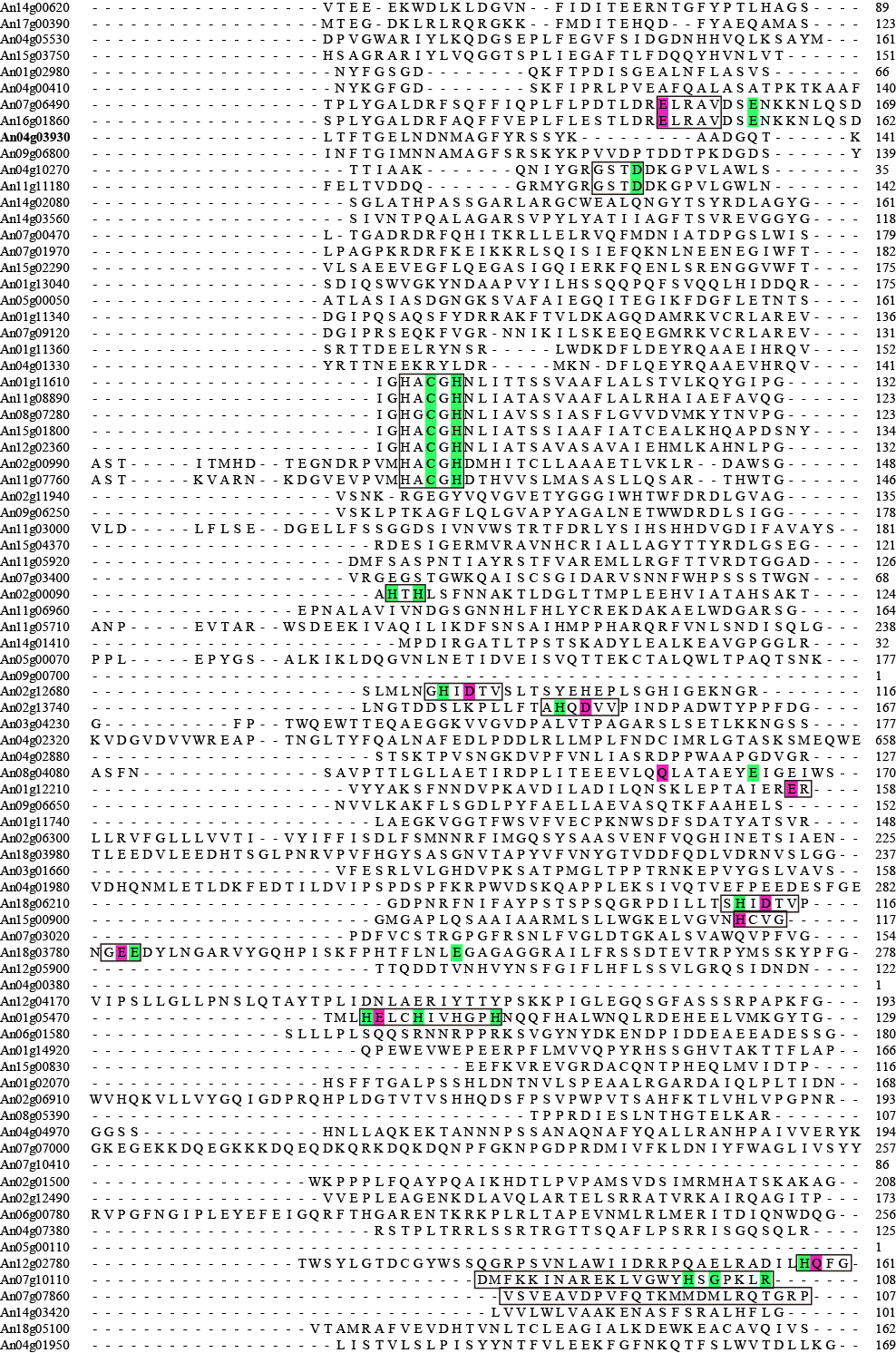


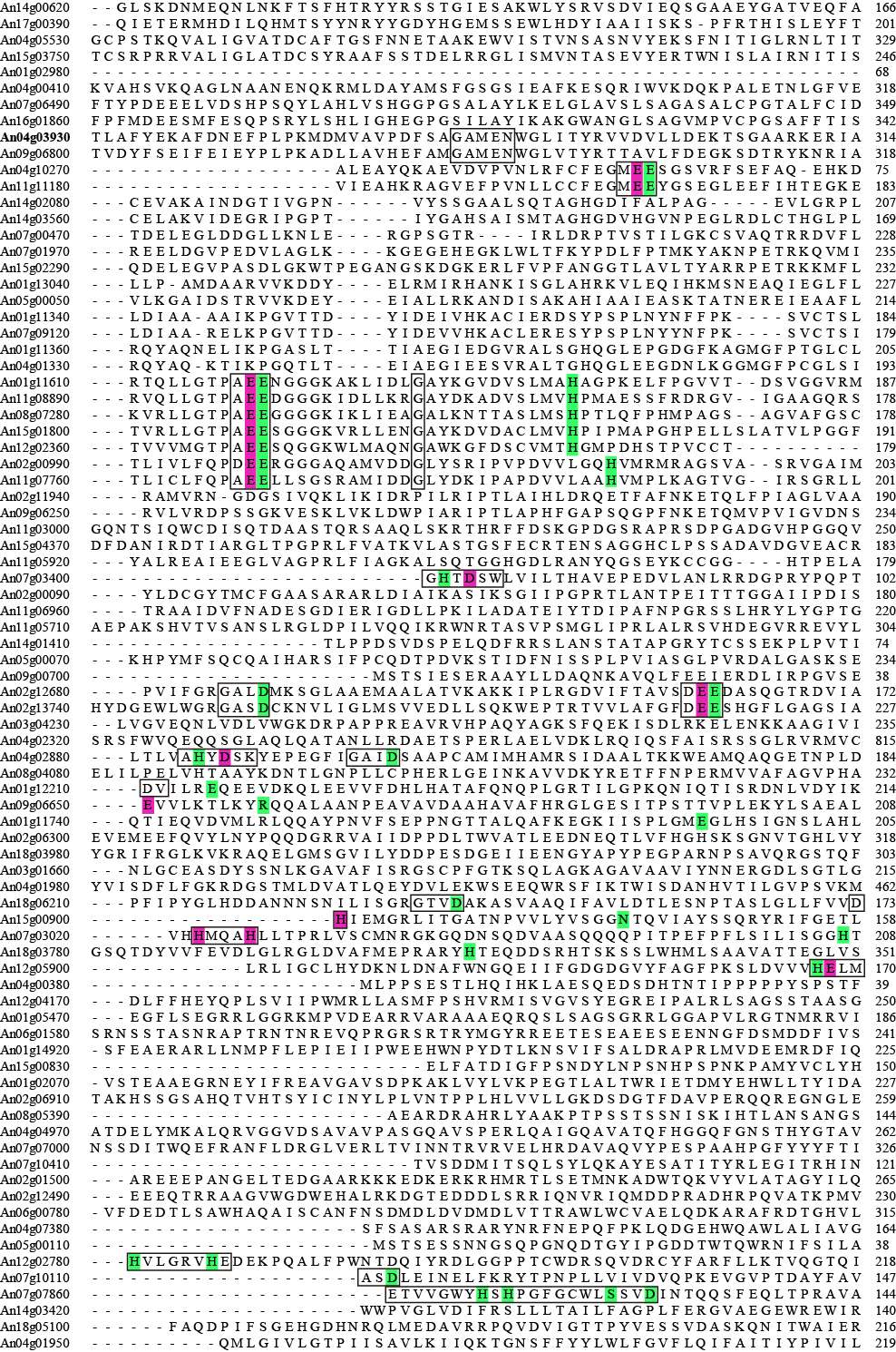


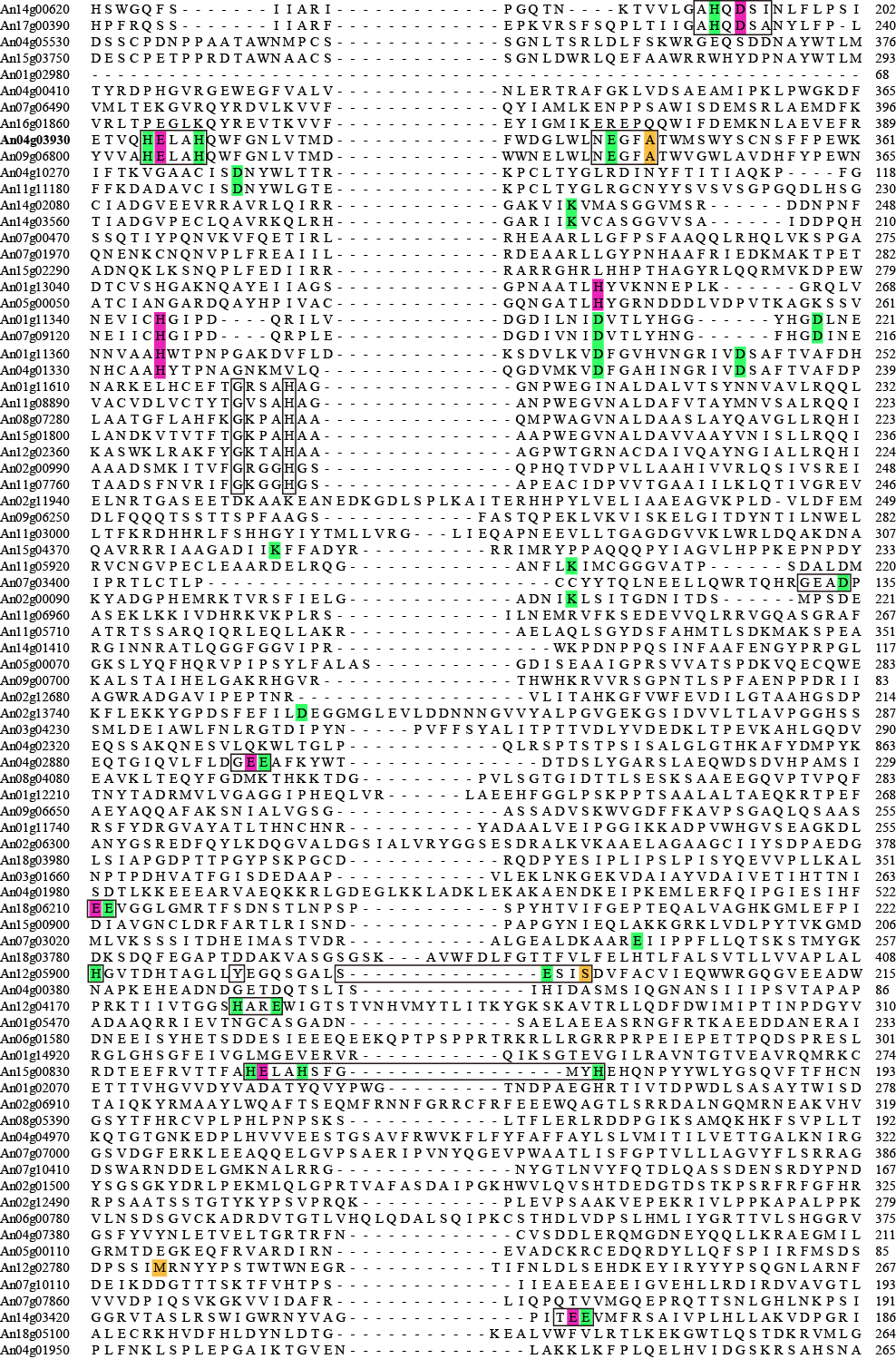


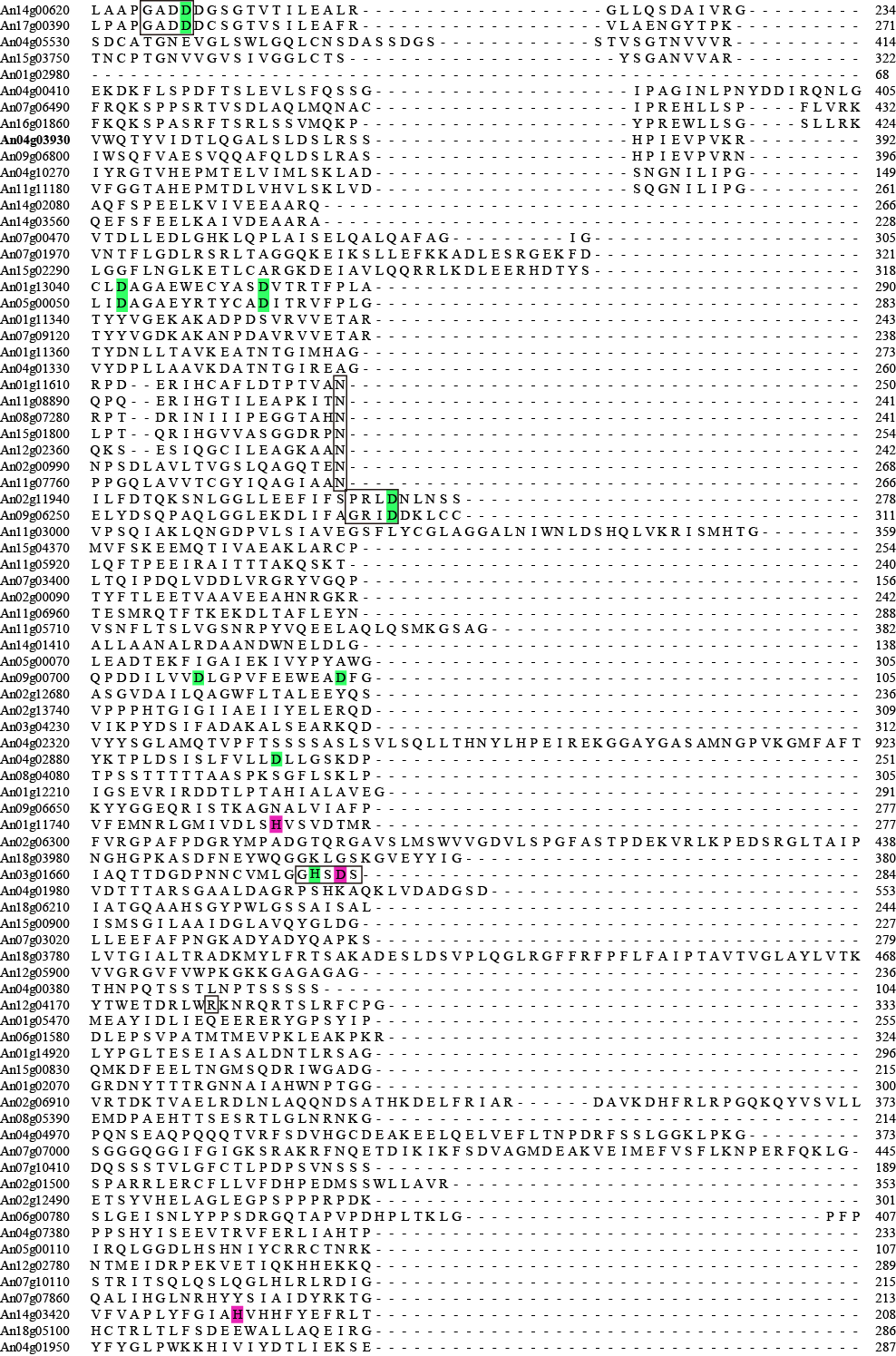


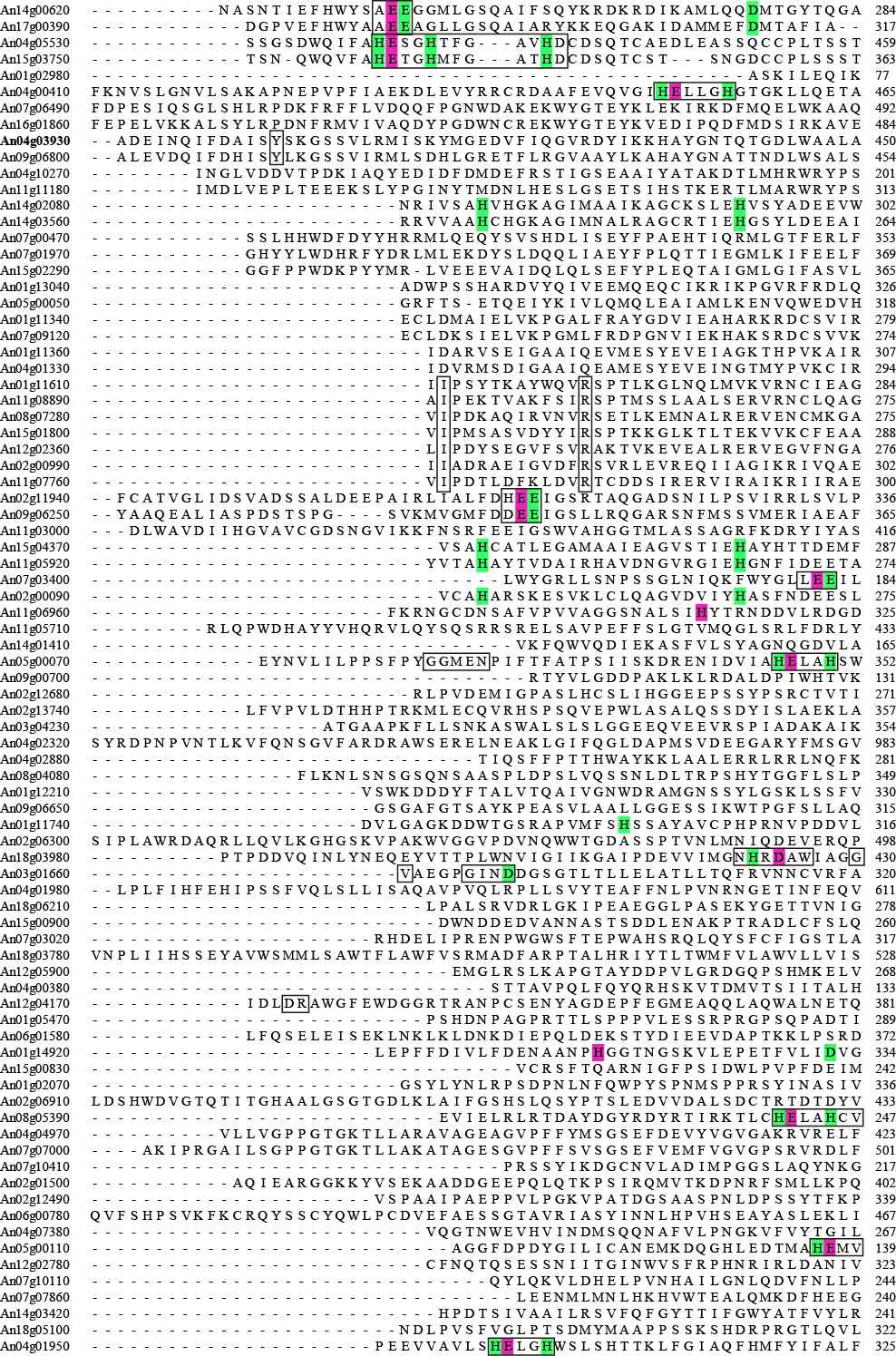


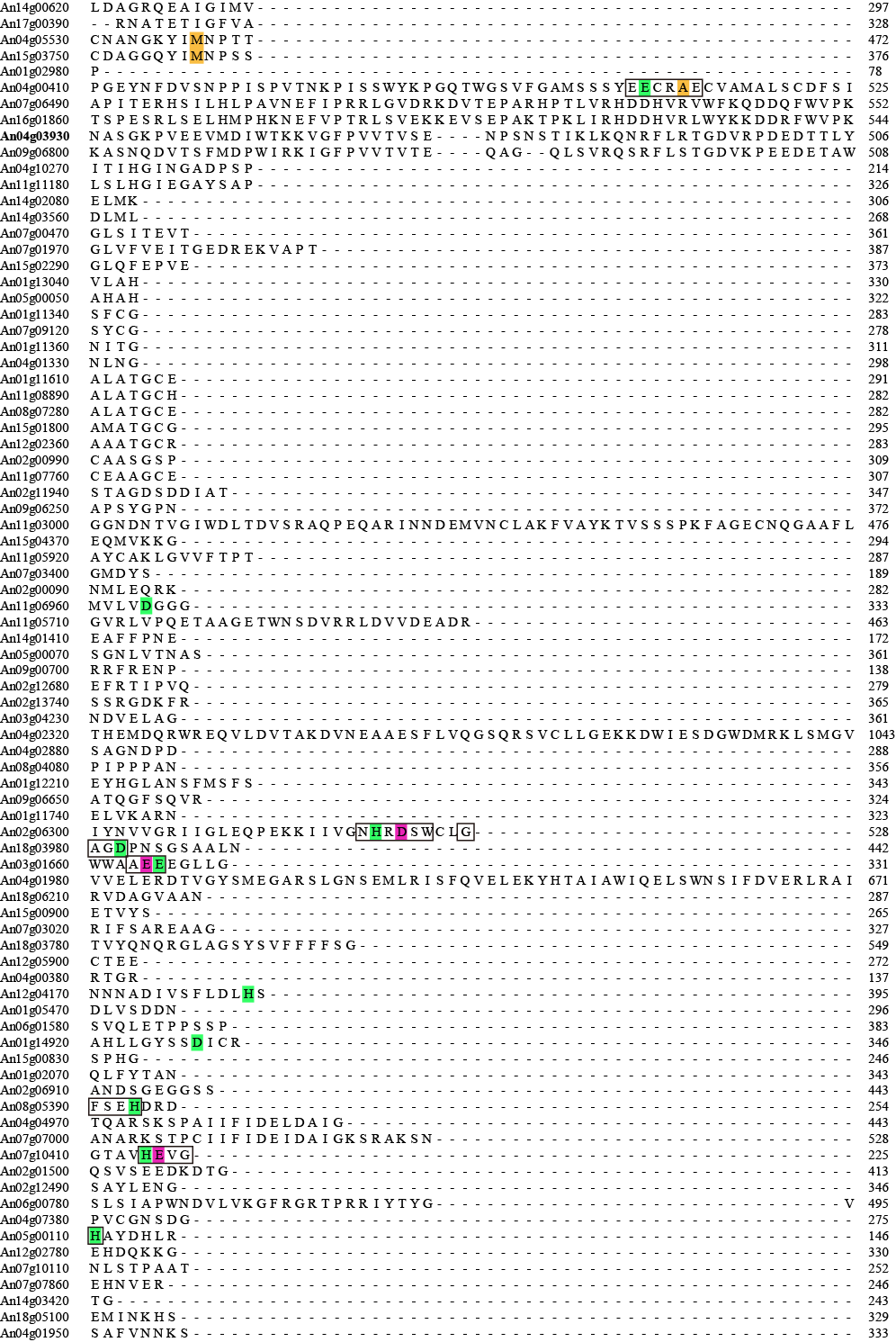


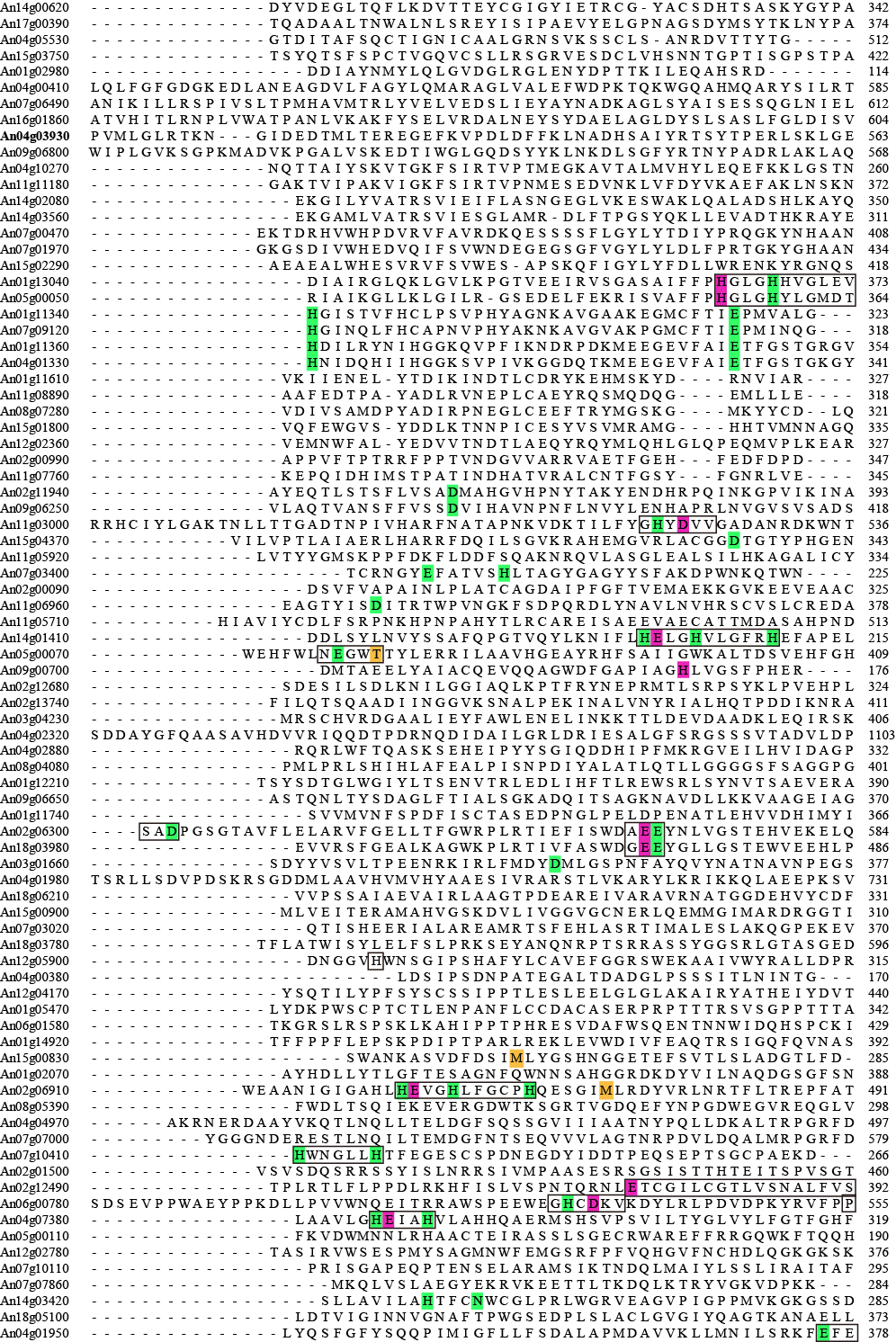


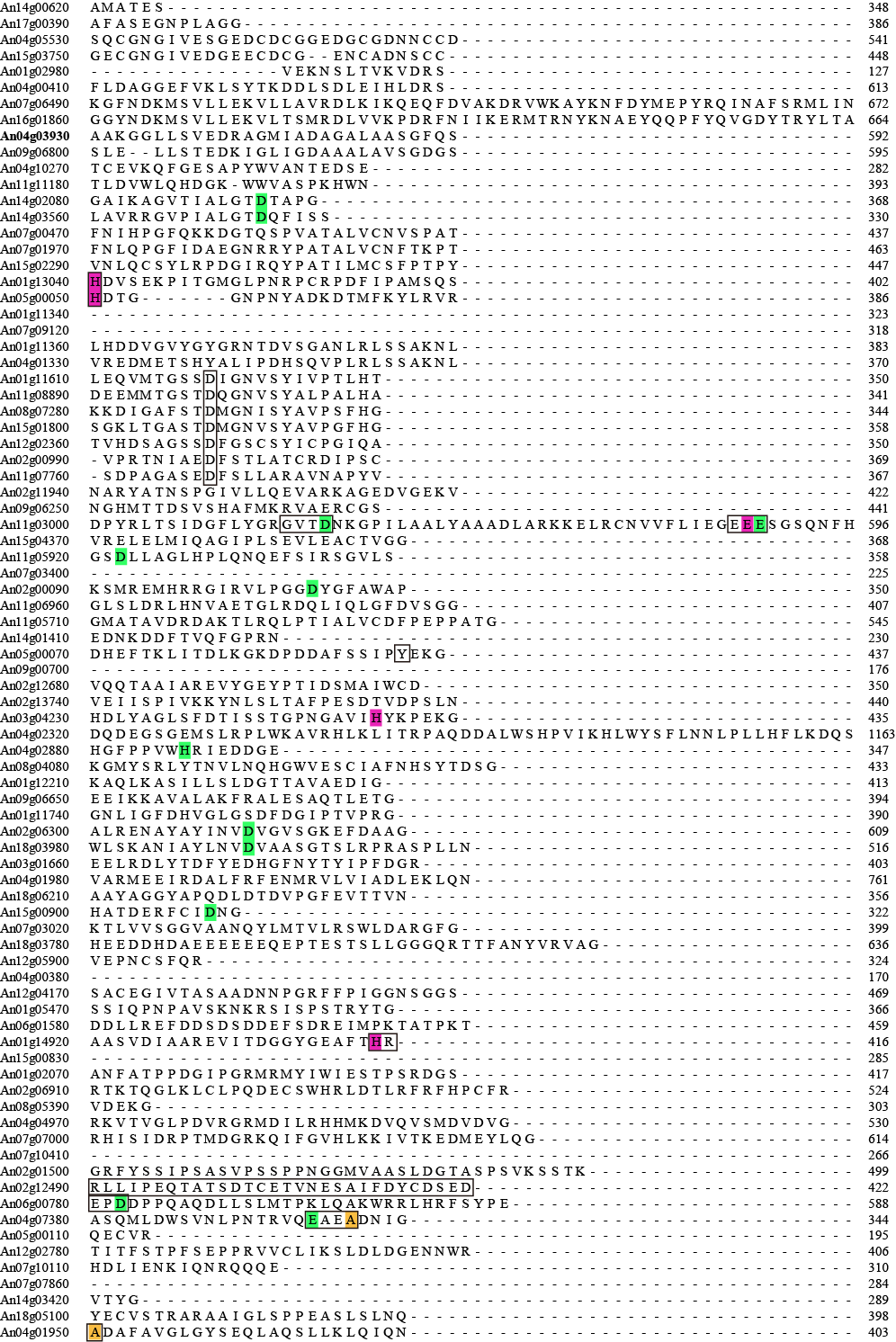


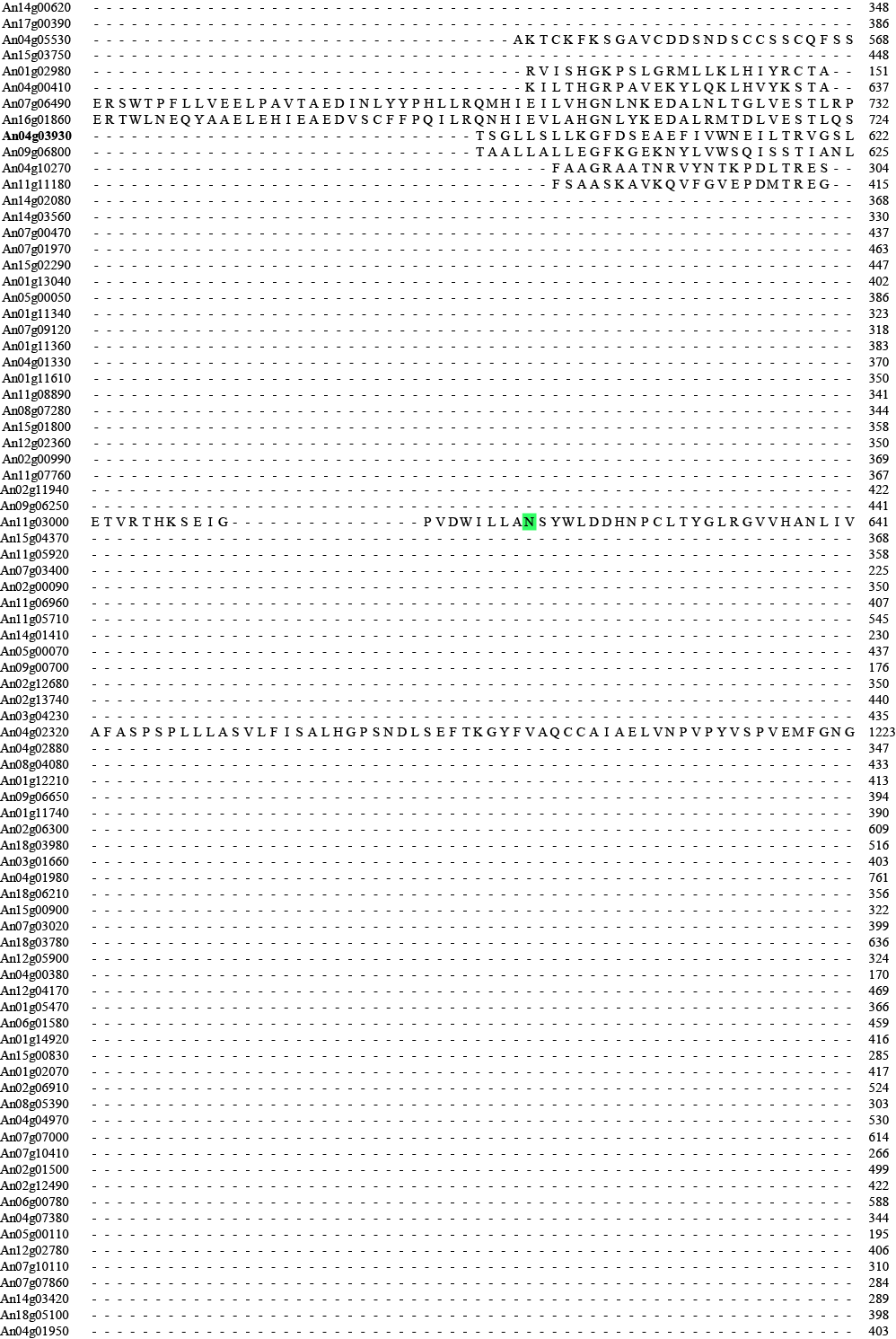


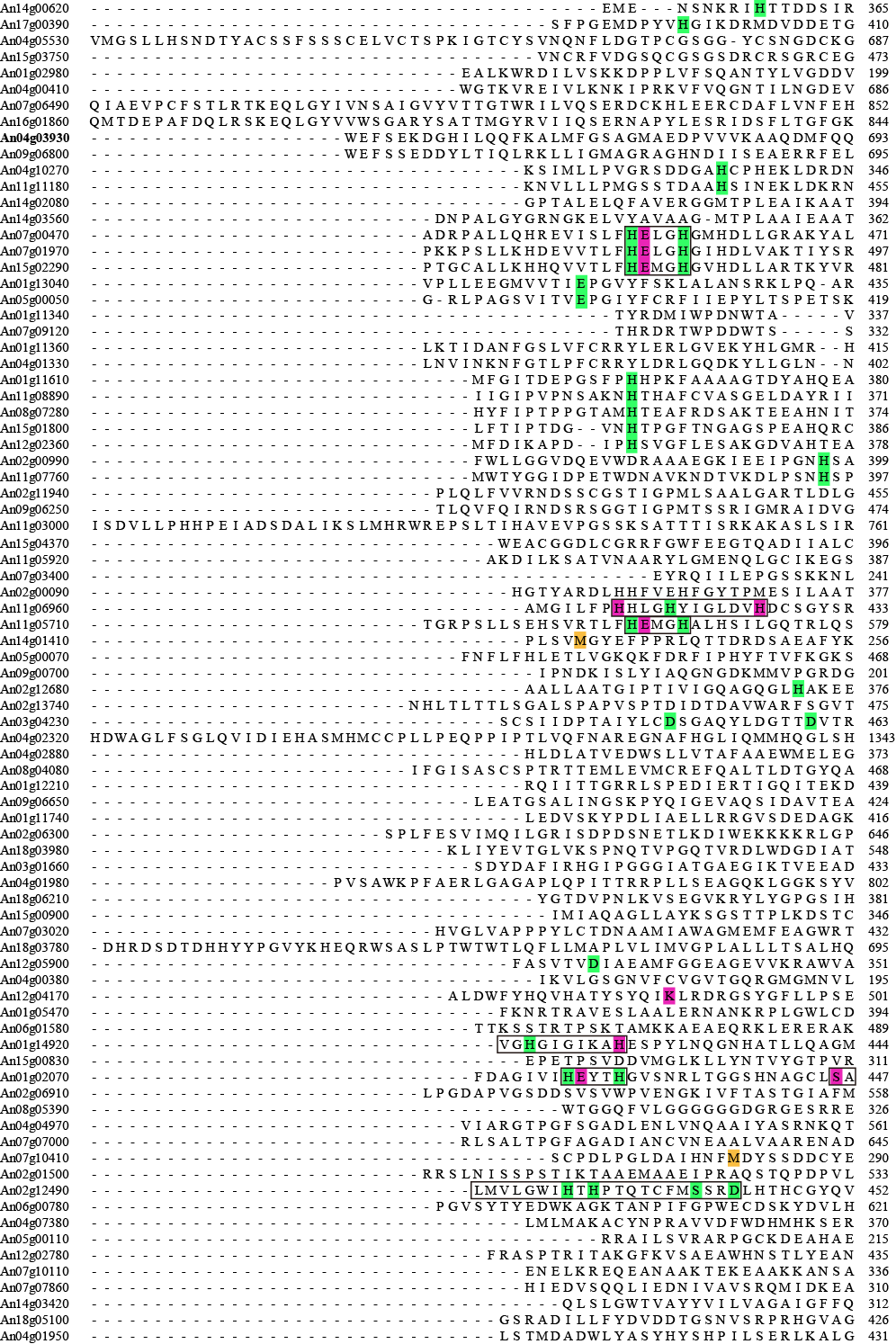


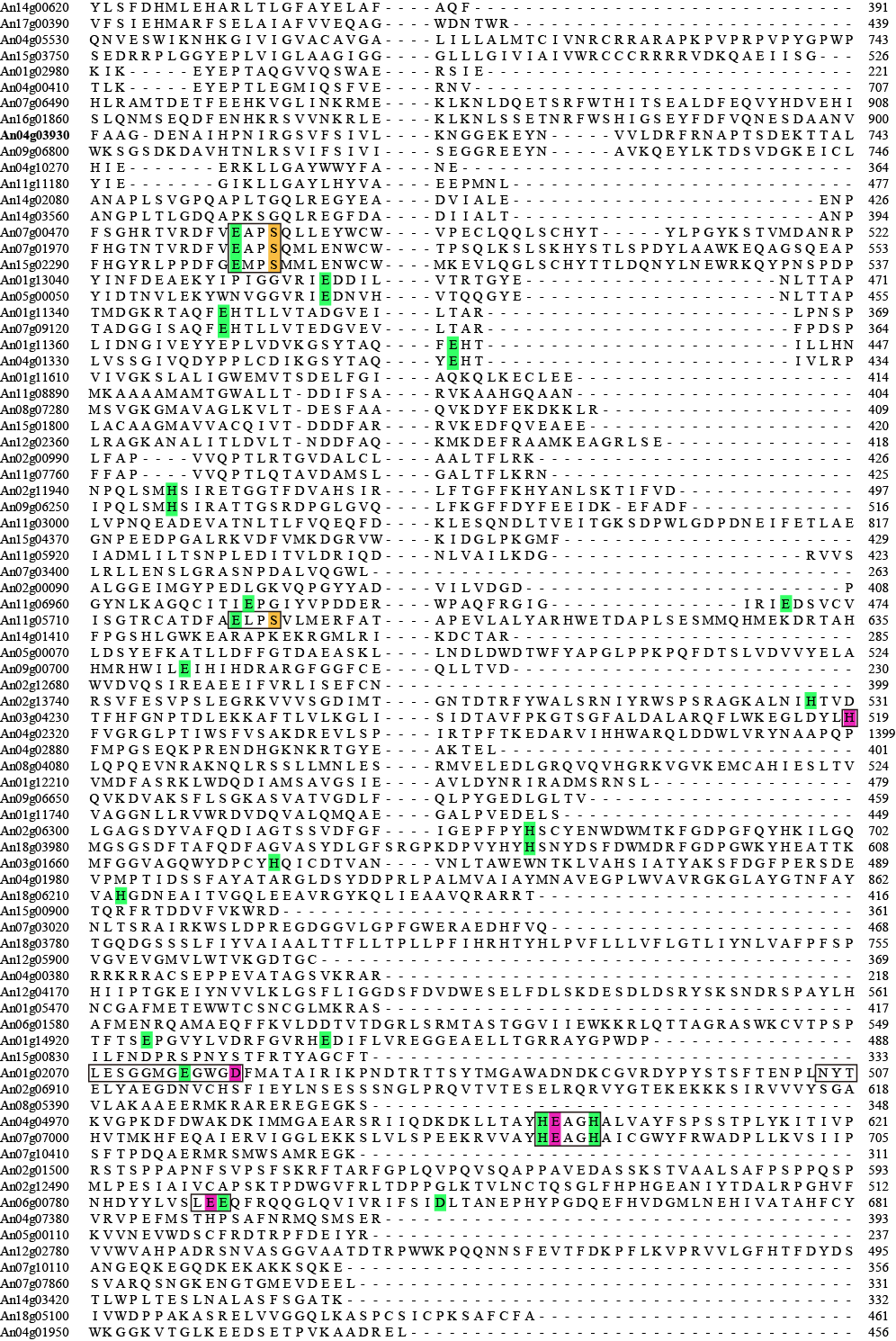


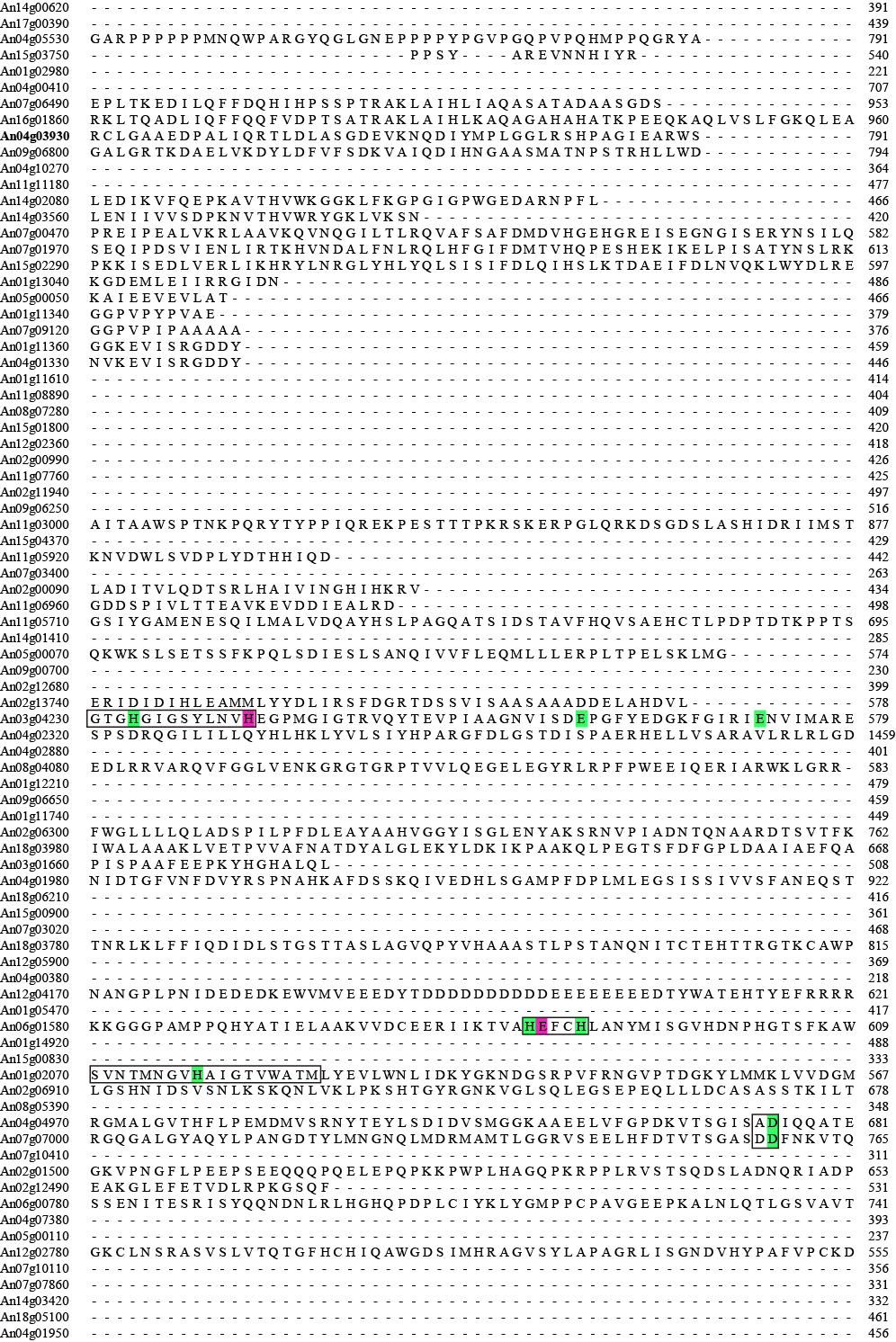


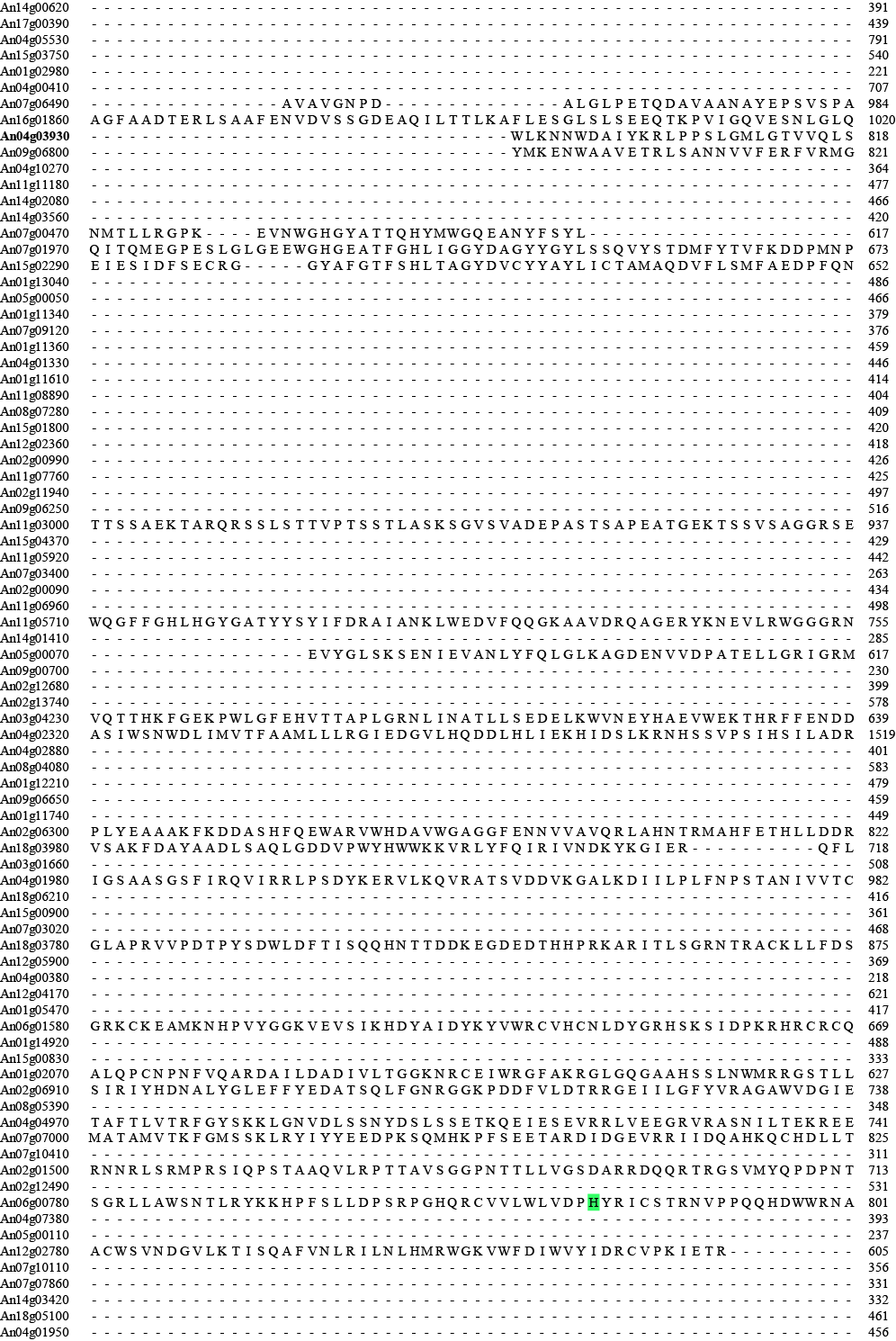


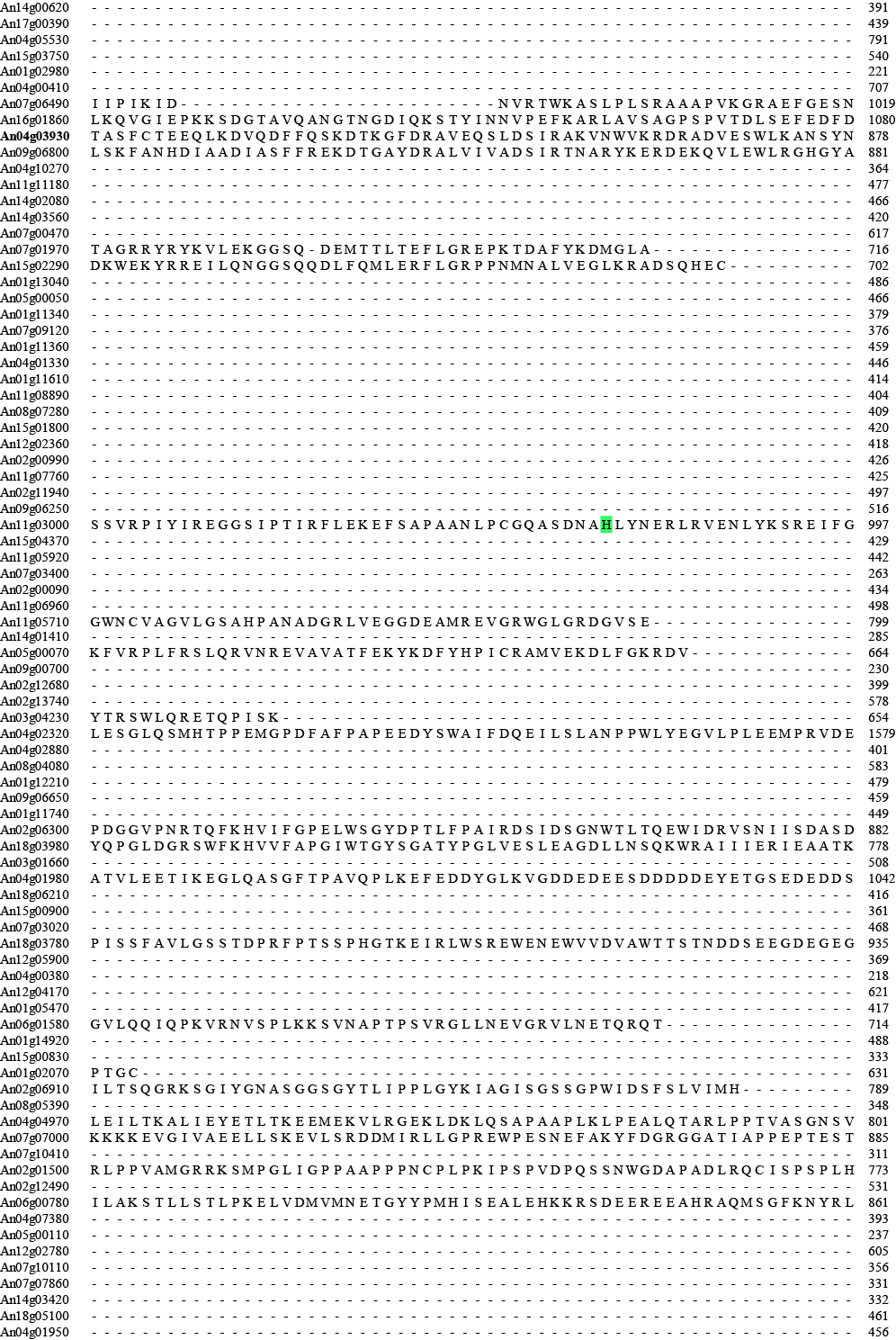

Supplement: Supplementary file 8 — Supplementary Figure S2. [file 41598_2020_80028_MOESM8_ESM.doc]
